# Persistent homology reveals strong phylogenetic signal in 3D protein structures

**DOI:** 10.1093/pnasnexus/pgae158

**Published:** 2024-04-17

**Authors:** Léa Bou Dagher, Dominique Madern, Philippe Malbos, Céline Brochier-Armanet

**Affiliations:** Université Claude Bernard Lyon 1, CNRS, VetAgro Sup, Laboratoire de Biométrie et BiologieÉvolutive, UMR5558, F-69622 Villeurbanne, France; Université Claude Bernard Lyon 1, CNRS, Institut Camille Jordan, UMR5208, F-69622 Villeurbanne, France; Université Libanaise, Laboratoire de Mathématiques, École Doctorale en Science et Technologie, PO BOX 5 Hadath, Liban; University Grenoble Alpes, CEA, CNRS, IBS, 38000 Grenoble, France; Université Claude Bernard Lyon 1, CNRS, Institut Camille Jordan, UMR5208, F-69622 Villeurbanne, France; Université Claude Bernard Lyon 1, CNRS, VetAgro Sup, Laboratoire de Biométrie et BiologieÉvolutive, UMR5558, F-69622 Villeurbanne, France

**Keywords:** topological data analysis, phylogenetics, persistent homology, protein 3D structure

## Abstract

Changes that occur in proteins over time provide a phylogenetic signal that can be used to decipher their evolutionary history and the relationships between organisms. Sequence comparison is the most common way to access this phylogenetic signal, while those based on 3D structure comparisons are still in their infancy. In this study, we propose an effective approach based on Persistent Homology Theory (PH) to extract the phylogenetic information contained in protein structures. PH provides efficient and robust algorithms for extracting and comparing geometric features from noisy datasets at different spatial resolutions. PH has a growing number of applications in the life sciences, including the study of proteins (e.g. classification, folding). However, it has never been used to study the phylogenetic signal they may contain. Here, using 518 protein families, representing 22,940 protein sequences and structures, from 10 major taxonomic groups, we show that distances calculated with PH from protein structures correlate strongly with phylogenetic distances calculated from protein sequences, at both small and large evolutionary scales. We test several methods for calculating PH distances and propose some refinements to improve their relevance for addressing evolutionary questions. This work opens up new perspectives in evolutionary biology by proposing an efficient way to access the phylogenetic signal contained in protein structures, as well as future developments of topological analysis in the life sciences.

**Significance Statement**

Determining the extent to which the 3D structures of proteins contain a phylogenetic signal is both a challenge and a major issue in evolutionary biology. Access to this information is key to studying very ancient evolutionary events, as protein structures are thought to evolve more slowly than sequences. However, the lack of reliable and efficient methods limits the use of structures. Here we propose an original approach based on persistent homology, an algorithmic method of topological data analysis. Analysis of 22,940 sequences and structures representing 763,648 homologous protein pairs shows that structures contain a strong phylogenetic signal that is efficiently captured by this approach, paving the way for the use of structures to study protein evolution.

## Introduction

Proteins are composed of one or more linear chains of amino acids, whose 3D fold (hereafter referred to as structure) is determined by the order and the physicochemical properties of the amino acids. In addition to their essential role in cells, proteins carry a phylogenetic (i.e. historical) signal. During evolution, amino acid substitutions, insertions, and deletions occur in protein sequences, which can affect their structure and function. These changes are transmitted over time vertically from parent to offspring or horizontally by gene transfer between unrelated organisms. Analyzing changes in protein sequences therefore provides information about the protein evolutionary history and the relationships between organisms. Phylogenetic inference by sequence comparison is the classic way to analyze the historical signal contained in protein sequences. Although very effective, this approach has some limitations (1–3). In particular, it is known that amino acids interact tightly, both functionally and spatially within protein structures, affecting their evolutionary trajectories. However, to reduce algorithmic complexity, the simplifying assumption that amino acids are independent of each other is often made, even though taking this information into account could improve the accuracy of evolutionary models, sequence alignments, and thus phylogenetic inference (4–8). In addition, most evolutionary models only consider substitutions and ignore the signal carried by insertions and deletions (indels), which could lead to an underestimation of the true evolutionary divergence between sequences (9, 10). Finally, the construction of reliable multiple alignments and phylogenetic trees is challenging, especially when sequences are highly divergent, because multiple substitutions (i.e. the occurrence of more than one substitution at a given amino acid site over time) lead to the progressive erosion of the phylogenetic signal (3, 11).

In this context, the analysis of protein structures has been proposed as an interesting alternative, as they are assumed to evolve more slowly than protein sequences (12). In fact, homologous proteins could share the same fold and other structural features even when their sequences have diverged beyond recognition (13–16). Furthermore, studying structures allows the spatial proximity of amino acids and their interactions to be considered. However, the use of structures to address evolutionary questions is still in its infancy due to the limited number of structures available in databases and methodological issues such as the lack of models accounting for structural evolution (see (17, 18) and references therein). The recent development of efficient structure prediction methods, such as Alphafold2 (19), has removed the first barrier by providing virtually unlimited access to structural information (20, 21). Methodologically, most studies are based on pairwise comparisons of structures, either by direct comparison of structures via structural alignment, or by comparison of vectors encoding their structural features (e.g. secondary structures, local features, atom density) (22–37). In most cases only the Cα atoms of the protein backbone are considered; the other atoms being ignored. These scores are then converted into distances and used to construct phylogenetic trees (38). However, as with protein sequence alignment, structural alignment is often difficult. The main consequence is that different alignments can be obtained depending on the algorithms used (16, 39). Alternative approaches to build phylogenies from structures are based on the taxonomic distribution of protein folds. Specifically, the presence or the absence of known folds in the organisms under study is encoded in a character matrix, which is then used to reconstruct phylogenetic trees by maximum parsimony or maximum likelihood methods (40–42).

In this work, we explore the potential of persistent homology (PH) to capture the phylogenetic signal from protein structures. PH is one of the most notable topological data analysis method (43) and a rapidly growing area of research with applications in a wide range of fields, including life sciences and biomedicine (44–51). PH provides robust and efficient algorithms for geometrically characterizing datasets represented by noisy finite point clouds (PCs). PH algorithms track the topological features (e.g. connected components, cycles, cavities, tunnels) of a given PC at different spatial scales. The principle is to examine the persistence of these features through multiscale filtration, turning the PC into a combinatorial object describing topological feature changes at increasing scales. This filtration thus captures the main and most robust topological features within the PC amidst the noise. These features are described by combinatorial signatures called PH-descriptors (e.g. barcodes, persistence landscapes), which represent the topological signatures of the PCs. For more details on PH see the Supplementary material text and Figs. S1–S7. The stability properties of PH ensure its robustness to noise by indicating how stable the information retained in the PH-descriptors is under small variations in the data (52) (see also Supplementary material text). PCs can be compared by computing distances (hereafter referred to as PH-distances) between their PH-descriptors, of which the most used are the Bottleneck (Btk), Wasserstein (Ws), and Landscape (Ls) distances (see Supplementary material text). PH is therefore particularly well suited to analyzing and comparing protein structures, which can be represented by noisy 3D PCs, whose points represent the spatial coordinates of the amino acid atoms. The noise in PCs can be due to, for example, the experimental precision with which structures are resolved, the uncertainty of bioinformatic predictions, or natural slight variations in protein shape. To date, PH has been mainly used to study protein folding, to classify protein structures, for protein engineering and directed protein evolution, and to study the effect of mutations on protein binding (53–61), but it has never been used to study the phylogenetic signal that may be contained in protein structures.

In this study, we performed a large-scale analysis of 518 protein families, comprising 22,940 protein sequences and structures, from 10 major prokaryotic groups. By comparing the corresponding 763,648 pairs of homologous proteins, we show that PH captures a strong signal in protein structures and that this signal is predominantly phylogenetic. This suggests that PH is an efficient way to extract phylogenetic information from protein structures and opens up a promising new area of application for PH in the life sciences.

## Results

### Reliability of predicted protein structures Alphafold2

The reliability of structure predictions for the 22,940 proteins considered was assessed by comparing the predictions of Alphafold2 with experimentally resolved structures from the RCSB PDB. All root-mean-square deviation (RMSD) values are between 0.05 to 3.53 Å (Fig. S8). In addition, 94% of the Cα have a confidence index (CI) greater than or equal to 70%, and 83% greater than or equal to 90%. Furthermore, 99% of the predicted structures have an average Cα CI greater than or equal to 70%, and 80% greater than or equal to 90% (Figs. S9 and S10). Overall, this suggests that protein structures predicted by Alphafold2 are reliable and can be used for further investigation.

### Persistent homology distances computation

**Fig. 1.**
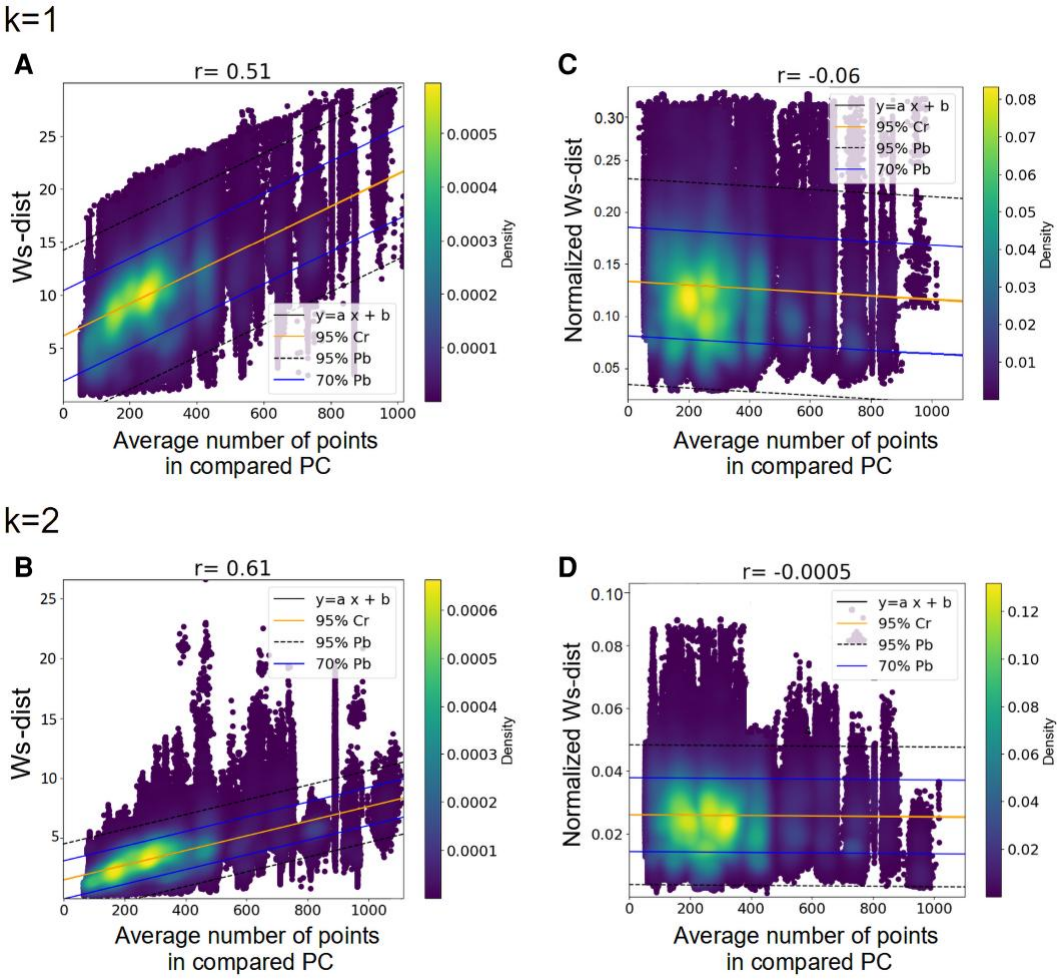
Correlation plots between the average number of points in PC(Cα) and Ws-distances calculated by AC filtration on PC(Cα) of the Alphafold2 predictions. Each plot contains 763,648 points, each corresponding to one pair of homologous proteins for which the average number of points of the two PC(Cα) (x-axis) is compared with the Ws-distance (y-axis). Results obtained with other PH-distances are shown as Table S1. A) Ws-distances (*k* = 1), *r* = 0.51, *P*-value = 2 × 10^−5^, B) Ws-distances (*k* = 2), *r* = 0.61, *P*-value = 1.7 × 10^−5^, C) Normalized Ws-distances (*k* = 1), *r* = −0.06, *P*-value = 0.1, D) normalized Ws-distances (*k* = 2), *r* = −0.0005, *P*-value = 0.2. For each panel, the central line is the 95% confidence regions in which the true regression line should belong. The two intermediate lines are the 70% interval within which future individual data points or observations should fall. The two most external lines are the 95% interval within which a future individual data point or observation should fall. The black line corresponds to the regression line (*y* = *ax* + *b*). Point colors correspond to the density values according to the density scale.

For each of the 763,648 pairs of homologous proteins, we calculated PH-distances (i.e. Btk-distances, Ws-distances, and Ls-distances) in homological dimension 1 and 2 by applying alpha complex (AC) and Vietoris-Rips (VR) filtrations to the corresponding structures. Here, PCs corresponding to Cα atoms (PC(Cα)) were used, as they are assumed to be representative of the whole structure and require less computation time than considering all atoms (55). At this stage, we find strong and significant correlations between PH-distances and the number of points in PC(Cα) (*r* = 0.39–0.61, all *P*-values <3.8 × 10^−3^, Fig. 1A and B and Table S1). This means that the number of Cα contained in structures has a significant impact on PH-distances. In order to limit this effect, we normalized PH-distances by the average number of Cα of the two compared PCs (Figs. S11 and S12). This normalization appears to be efficient because previously observed correlations become nonsignificant (*r* = −0.11 – −5 × 10^−4^, all *P*-values >0.1, Fig. 1C and D and Table S1). Accordingly, we used normalized PH-distances in all analyses.

Since the shape of proteins is constrained by various physicochemical considerations, such as covalent or hydrogen bonds, we investigated whether the PH-distances calculated between pairs of homologous protein structures contained any biological information or whether they could have been observed by chance alone. We therefore compared the ranges of Btk-distances, Ws-distances, and Ls-distances calculated between randomly selected 7,500 pairs of homologous protein structures and 7,500 pairs of nonhomologous protein structures. We find that the latter are significantly larger than the former, in both homological dimensions 1 and 2 (Fig. S13). This means that the PH-distances calculated from homologous proteins cannot have been obtained by chance and contain biological information.

We also observe that the normalized Ls-distances and Ws-distances are close to each other, but larger than the Btk-distances (Figs. S11 and S12). This difference is expected because the Btk-distance corresponds to the largest distance between pairs of barcode intervals matched in the most efficient way, whereas the other two are (i) the sum of all distances between barcode intervals matched in the most efficient way (Ws-distance) and (ii) pairs of persistent landscape functions matched from largest to smallest (Ls-distance) (see Supplementary material text). The Btk-distance is therefore coarser and probably less efficient at capturing subtle information in protein structures. We also find that PH-distances in dimension 2 (*k* = 2) are systematically smaller than those in dimension 1 (*k* = 1) (Figs. S11 and S12), suggesting that cavities may be more conserved than cycles in protein structures.

### Persistent homology captures strong phylogenetic signal in protein structures

**Fig. 2.**
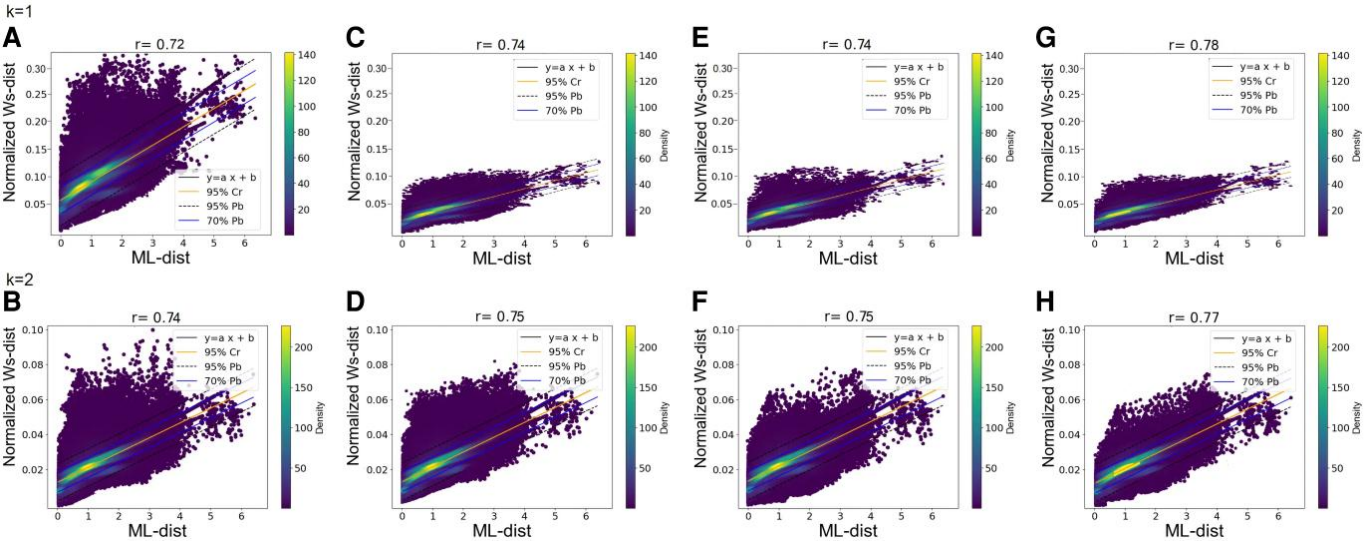
Correlation plots between ML-distances and Ws-distances calculated by AC filtration on PC(Cα) of the Alphafold2 predictions. Each plot contains 763,648 points, each corresponding to a pair of homologous proteins for which the ML-distance (x-axis) is compared with the normalized Ws-distance (y-axis). A) Normalized Ws-distances (*k* = 1) calculated on all Cα (*r* = 0.72, *P*-value = 5 × 10^−4^), B) normalized Ws-distances (*k* = 2) calculated on all Cα (*r* = 0.74, *P*-value = 2 × 10^−4^), C) as in A) except that the Cα corresponding to amino acid indels were omitted (*r* = 0.74, *P*-value = 3 × 10^−5^), D) as in (B) except that the Cα corresponding to amino acid indels were omitted (*r* = 0.75, *P*-value = 7 × 10^−4^), E) as in A) except that the Cα with CI < 70% were omitted (*r* = 0.74, *P*-value = 3 × 10^−5^), F) as in B) except that the Cα with CI < 70% were omitted (*r* = 0.75, *P*-value = 6 × 10^−4^), G) as in A) except that the Cα corresponding to amino acid indels and those with CI < 70% were omitted (*r* = 0.76, *P*-value = 9 × 10^−4^), H) as in B) except that the Cα corresponding to amino acid indels and those with CI < 70% were omitted (*r* = 0.77, *P*-value = 6 × 10^−4^). For details regarding confidence and prediction bands, see Fig. 1.

To unravel the nature of the signal captured by PH, and in particular to determine the extent to which it is phylogenetic, we compared PH-distances (i.e. Btk-distances, Ws-distances, and Ls-distances) computed on structures for each of the 763,648 pairs of homologous proteins with evolutionary (EV)-distances calculated from Maximum likelihood trees (ML-distances) and multiple alignments (*p*-distances). The results show significant correlations ranging from 0.43 to 0.74 (all *P*-values <0.008, Table S2). The strongest correlations are observed with Ws-distances by applying AC filtrations (Fig. 2A and B), while the weakest correspond to Btk-distances computed from the VR filtration (Table S2).

Closer examination reveals five notable trends (Table S2). First, slightly higher correlations are observed with ML-distances than with p-distances (*r* = 0.44–0.74 and *r* = 0.43–0.72, respectively), the former being a better estimate of true evolutionary distances as it considers multiple substitutions in sequences. Second, the AC filtration provides stronger correlations than VR (*r* = 0.45–0.74 and *r* = 0.43–0.67, respectively). This may be because AC filtration is more appropriated to protein structures, as it considers local density within PCs to decide whether or not to connect two nearby points at each filtration step, whereas VR connects all pairs of points that come into contact, creating all corresponding simplexes (see Supplementary material text). Third, the Ws-distance provides stronger correlations (*r* = 0.63–0.74) than the other two PH-distances (*r* = 0.43–0.66), whatever the homological dimension considered. This may reflect the fact that the Ws-distance considers all pairwise matched intervals, not just the most distant. It is therefore based on a more complete quantification of the information contained in barcodes than the Btk-distance and is better able to capture subtle differences in the geometry of the protein structures. Furthermore, while interval matching between barcodes is optimal for Ws-distances, the matching between persistent landscapes is not for Ls-distances. Fourth, PH-distances in dimensions 1 and 2 show similar correlations (*r* = 0.44–0.72 [*k* = 1] and *r* = 0.43–0.74 [*k* = 2]), suggesting that both cycles and cavities contain phylogenetic signal. Finally, we find significant correlations between the ML-distances and the PH-distances for the 518 protein familes (ranging from 0.30 to 0.90) that cannot be observed by chance (Fig. S14), again demonstrating that PH allows a clear phylogenetic signal to be captured in structures.

Overall, our analysis reveals a strong correlation between PH-distances and EV-distances. This suggests that geometrical variations observed in structures correlate well with substitutions occurring in sequences and that the signal captured by PH in structures is mainly phylogenetic. We also show that PH-distances, particularly the Ws-distance computed from AC filtration, is most efficient at capturing this signal. Accordingly, in the following, we focused on Ws-distances calculated using the AC filtration.

### Information carried by indels

Comparisons between PH-distances and EV-distances show a number of outliers corresponding to pairs of homologous proteins with unexpectedly high PH-distances (Fig. 2A and B). Close examination of sequence alignments shows that the incriminated sequences contain large indels (see examples shown as Figs. S15–S20). In fact, we find that the correlation between PH-distances and EV-distances became weaker as the number of gaps in sequences being compared increased (Fig. S21). As expected, this is more pronounced when Ws-distances are compared with ML-distances, as these are derived from phylogenetic trees calculated after the alignment trimming step, which removes most of the indels in the sequences (Fig. S21A and B). Weaker but still significant correlations are also observed with p-distances, despite they are calculated on untrimmed multiple alignments (Fig. S21C and D).

To measure the contribution of indels, we computed Ws-distances by omitting Cα corresponding to amino acid indels. As expected, the correlation between PH-distances and EV-distances and the number of gaps in sequences disappears (Fig. S22). Overall, we observe a reduction in the dispersion around the regression line and a slight increase in the correlation coefficients with both ML-distances (from 0.72 to 0.74 [*k* = 1] and from 0.74 to 0.75 [*k* = 2], Fig. 2C and D) and p-distances (from 0.70 to 0.72 [*k* = 1] and from 0.72 to 0.74 [*k* = 2], not shown). This increase may seem modest, but it should be remembered that most of protein sequences studied here contain no or very few indels. If we consider only protein families whose sequences contain indels, the increase in correlations is much greater (from 0.70 to 0.74 [*k* = 1] and 0.69 to 0.76 [*k* = 2] for ML-distances and from 0.68 to 0.73 [*k* = 1] and 0.68 to 0.74 [*k* = 2] for p-distances). This suggests that indels in sequences generate a strong signal in protein structures that is efficiently captured by PH.

### Effect of the quality of 3D structure predictions

We then asked to what extent the quality of structure prediction affects PH-distances. If protein structures do indeed contain a phylogenetic signal, we expect that the more realistic the predicted structures, the stronger the phylogenetic signal captured. This is indeed the case as correlations between Ws-distances and ML-distances are stronger the higher the average CαCI of the structure predictions (*r* = 0.50, *P*-value = 8 × 10^−5^ [*k* = 1] and *r* = 0.45, *P*-value = 2 × 10^−4^ [*k* = 2]). Therefore, it would be interesting to investigate in more details whether correlations between PH-distances and EV-distances could be an indicator of the quality and the reliability of protein structure predictions. To go further, we calculated Ws-distances by considering only Cα with a CI greater than or equal to 70%. We observe a slight increase in correlations with ML-distances from 0.72 to 0.74 (*k* = 1) and from 0.74 to 0.75 (*k* = 2) (Fig. 2A–E and B–F, respectively), meaning that amino acids with low CI, although few in numbers (6%), have an impact on the correlations between Ws-distances and ML-distances. These correlations reach 0.78 (*k* = 1) and 0.77 (*k* = 2) when Cα corresponding to indels are also omitted (Fig. 2G and H).

### Effect of genetic divergence within taxa on the intensity of the phylogenetic signal contained in protein structures

**Fig. 3.**
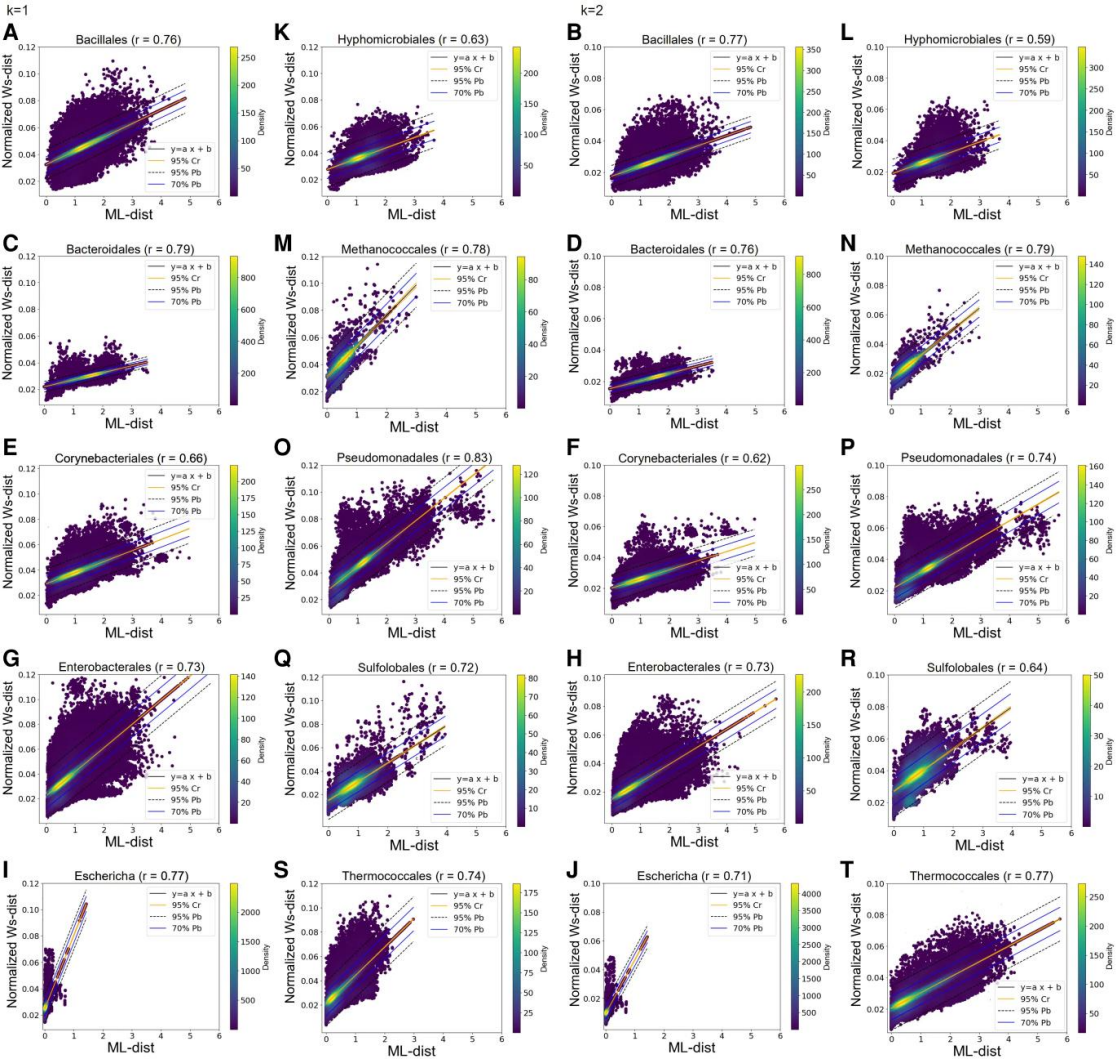
Correlation plots between ML-distances and Ws-distances computed for each of the 10 taxa. On a given plot, each dot corresponds to a pair of homologous proteins for which the ML-distance (x-axis) is compared to the normalized Ws-distance computed on PC(Cα with CI > 70%) when indels are omitted (y-axis). All *P*-values are less than 4 × 10^−5^. A) *Bacillales*: 170,354 pairs of homologous proteins (*k* = 1), B) *Bacillales*: 170,354 pairs of homologous proteins (*k* = 2), C) *Bacteroidales*: 40,402 pairs of homologous proteins (*k* = 1), D) *Bacteroidales*: 40,402 pairs of homologous proteins (*k* = 2), E) *Corynebacterales*: 154,513 pairs of homologous proteins (*k* = 1), F) *Corynebacterales*: 40,402 pairs of homologous proteins (*k* = 2), G) *Enterobacterales*: 196,915 pairs of homologous proteins (*k* = 1), H) *Enterobacterales*: 196,915 pairs of homologous proteins (*k* = 2), I) *Escherichia*: 23,879 pairs of homologous (*k* = 1), J) *Escherichia*: 23,879 pairs of homologous proteins (*k* = 2), K) *Hyphomicrobiales*: 38,679 pairs of homologous proteins (*k* = 1), L) *Hyphomicrobiales*: 38,679 pairs of homologous proteins (*k* = 2), M) *Methanococcales*: 2,711 pairs of homologous proteins (*k* = 1), N) *Methanococcales*: 2,711 pairs of homologous proteins (*k* = 2), O) *Pseudomonadales*: 75,834 pairs of homologous proteins (*k* = 1), P) *Pseudomonadales*: 75,834 pairs of homologous proteins (*k* = 2), Q) *Sulfolobales*: 11,453 pairs of homologous proteins (*k* = 1), R) *Sulfolobales*: 11,453 pairs of homologous (*k* = 2), S) *Thermococcales*: 48,908 pairs of homologous proteins (*k* = 1), T) *Thermococcales*: 48,908 pairs of homologous proteins (*k* = 2). For details regarding confidence and prediction bands, see Fig. 1.

The 10 taxonomic groups studied cover a wide range of genetic divergence. This is illustrated by comparing the distribution of ML-distances between pairs of homologous protein sequences within each taxon (Fig. S23). In particular, medians of these distributions range from 0.02 for *Escherichia* to 1.53 for the *Bacteroidales*. As expected, due to the strong correlation between PH-distances and EV-distances highlighted above, Ws-distances are smaller for taxonomic groups with lower genetic divergence, whatever the homological dimension considered (Figs. S24 and S25). However, as structures are assumed to be more conserved than sequences, they are expected to contain a weaker phylogenetic signal, or even no signal when proteins are very similar at the sequence level. According to this hypothesis, we would expect correlations between Ws-distances and ML-distances to be weak for taxonomic groups with the lowest genetic diversity (e.g. *Escherichia*, *Thermococcales*, *Methanococcales*, *Enterobacterales*). We find that this is not the case (Fig. 3), implying that although structures evolve less rapidly than sequences, they contain a strong phylogenetic signal even at small evolutionary scale, which is efficiently captured by PH.

### Comparison of the information carried by the different types of atoms in amino acids

**Fig. 4.**
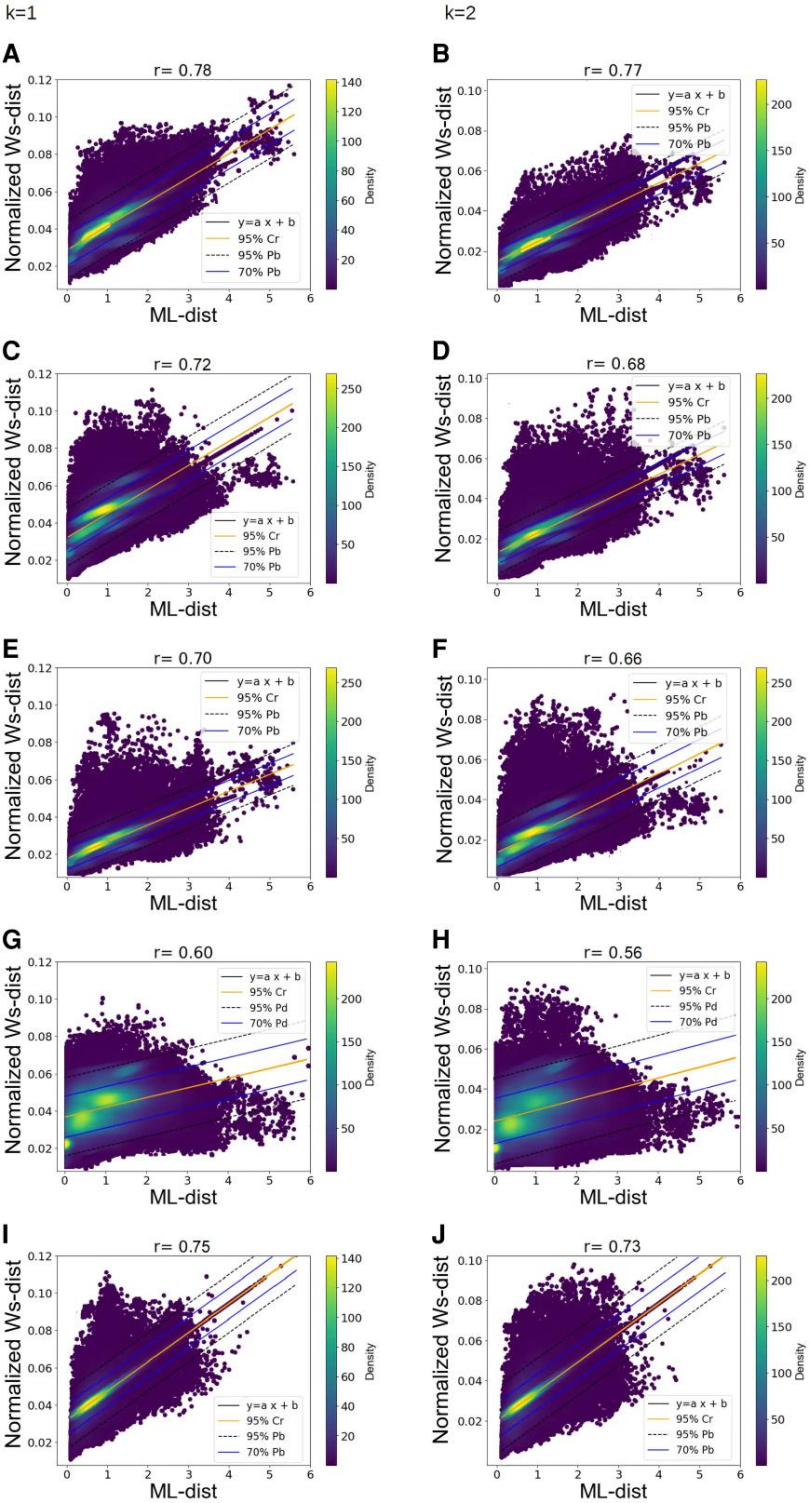
Correlation plots between ML-distances and Ws-distances according to the type of considered atoms. Each plot contains 763,648 points, each corresponding to a pair of homologous proteins for which the ML-distance (x-axis) is compared to the normalized Ws-distance (y-axis). To calculate distances, atoms corresponding to amino acids whose Cα CI > 70% or corresponding to indel were omitted. All *P*-values are less than 3 × 10^−4^ A) PC(Cα) (*k* = 1), B) PC(Cα) (*k* = 2), C) PC(All-N) (*k* = 1), D) PC(All-N) (*k* = 2), E) PC(All-O) (*k* = 1), F) PC(All-O) (*k* = 2), G) PC(All-C) (*k* = 1), H) PC(All-C) (*k* = 2), I) PC(All-Atoms) (*k* = 1), J) PC(All-Atoms) (*k* = 2). For details regarding confidence and prediction bands, see Fig. 1.

So far, we focus on PC(Cα), as it is expected to provide a good compromise between information and computation time (55). However, amino acids differ in the number, type and spatial organization of their side chain atoms (Figs. S26–S28). We therefore wonder to what extent focusing on Cα might lead to a loss of information or introduce biases. We thus compared ML-distances with Ws-distances computed on PC(Cα), PC(All-Atoms), PC(All-C), PC(All-N), and PC(All-O). We find that the correlations obtained with PC(Cα) are higher (*r* = 0.78 [*k* = 1] and *r* = 0.77 [*k* = 2], Fig. 4A and B) than with PC(All-N), PC(All-O) and PC(All-C) (*r* = 0.72, 0.70, and 0.60 [*k* = 1] and *r* = 0.68, 0.66, and 0.56 [*k* = 2], respectively, Fig. 4C–H). This could suggest that PC(All-N), PC(All-O), and PC(All-C) contain a weaker phylogenetic signal, or more probably, reflects variations in the number of points in PCs. Indeed, the number of N, O, and C atoms in side chains varies depending on the amino acids considered (Figs. S26–S28). In the case of Cα, variations in their number within PCs(Cα) lead to a slight decrease in the correlation between PH-distances and ML-distances (see above). Accordingly, when considering O, N, or C atoms of side chains, similar effects are expected. In support of this hypothesis, the weakest correlation is observed for carbon atoms whose number varies the most in side chains. This suggests that the signal due to variations in the number of O, N, and C atoms in side chains, is strong enough to partially obscure the phylogenetic signal contained in protein structures. Surprisingly, the correlations calculated using PC(All-Atoms) and PC(Cα) are close (*r* = 0.75 and 0.78 [*k* = 1], and *r* = 0.73 and 0.77 [*k* = 2], Fig. 4A, B, I, and J). One hypothesis would be that the combination of the strong phylogenetic signal contained in PC(Cα), and to a lesser extent in PC(All-N) and PC(All-O), compensates for the noise associated with high variations in the number of C atoms in the side chains.

## Discussion and conclusion

Studying the historical information contained in protein sequences has limitations especially when sequences are highly divergent (11, 62). In that context, structures have been proposed as an alternative to sequences, as they are assumed to evolve slower (6, 12, 17). Until now, their use for phylogenetic inference has been limited by the absence or the insufficient number of structures available for most protein families. This is about to change, thanks to the development of methods based on artificial intelligence that allow virtually unlimited access to protein structures (63). This is opening up a new era in structural biology and paves the way for using structures in all fields of life sciences (64, 65), including the study of protein evolution (15). However, translating structural distances into evolutionary information remain a challenge by the lack of models allowing to bridge the gap between protein structure and sequence evolution (18, 66, 67).

This work, which aims to explore new ways of capturing the signal contained in structures, is part of that dynamic. Here, we investigate the potential of PH, an original approach from the topological data analysis, to capture a phylogenetic signal from protein structures. PH allows protein structures, represented as PCs, to be compared according to their intrinsic geometric characteristics, in particular the presence of cycles (in homological dimension 1) and cavities (homological dimension 2) formed by the position of their amino acid atoms in 3D space. The originality of PH is that it does not rely on direct structures comparisons. Indeed, PH proposes an algebraic formalism for measuring the geometric features of 3D representations by abstracting their multiscale organization through a structure filtration algorithm. In this way, the entire structure of the protein is considered and, unlike most approaches, PH avoids the need to align structures, a critical step in most analyses (16, 39). One of the major advantages of PH is that PH-descriptors are invariant to small variations in the coordinates of points in PCs. Therefore, PH allows the study of dynamical entities, such as protein structures, from one of their static representations. This good stability of the descriptors results from the fact that the geometric characteristics of a structure are calculated from a filtration of its PC representation along a gradient of spatial scales. This filtration progressively links points within the PC. Intrinsic geometric features of the PC will persist significantly along this filtration, while those resulting from small variations will be ephemeral and will not be considered to compute PH-distances. This property allows to limit the effect of microvariations in the coordinates of the atoms due to the dynamical nature of protein structures (i.e. slight topological oscillations and variations) and atomic uncertainties in the coordinates of atoms in bioinformatically predicted or experimentally solved protein structures.

Examination of a large dataset consisting of 518 protein families, representing more than 700,000 pairs of homologous proteins, reveals a strong and significant linear relationship between distances computed on protein sequences and structures in both homological dimensions 1 and 2. This means that variations in the geometry of structures measured by the persistence of cycles and cavities constitute a strong phylogenetic signal that is efficiently captured by PH. Furthermore, PH is a very sensitive method, as it detects the phylogenetic signal even when the proteins studied are very similar at the sequence level (i.e. differing by only a few amino acids), as well as when they are very divergent (i.e. differing in average by more than two to four substitutions per site). However, we also observe that the strength of this correlation varies among the 518 families of homologous proteins analyzed. This agrees with previous reports (12, 68). The next step will be to determine which factors may be responsible for the discrepancies between PH-distances and evolutionary distances. For example, it has been proposed that (i) sequences may follow a structurally constrained neutral evolution, which allow sequences to evolve freely as long as the structure is preserved (67, 69–72), (ii) that protein interactions may constrain the evolution of structures (73), or (iii) that duplication, transfer and recombination of short protein segments may have played an important role in the evolution of structures, leading to local areas of similarity within proteins or even between distant proteins (74–77).

Previous studies have shown that amino acid insertions and deletions in protein sequences could impact deeply protein structure (78–80). For example, although they are rarer than amino acids substitutions, they could account for more than 45% of structural variation (81). Although we confirm the effect of deletions and insertions on the differences observed between the distances calculated from sequences and from structures, this effect remains relatively weak. This may reflect the fact that the different studies are not based on the same sets of proteins and/or that PH is intrinsically less sensitive to the effect of amino acid insertions and deletions in proteins than other methods because it measures variations in the geometric properties of proteins rather than overall changes in protein shapes. This issue requires further investigation. To limit the biases, it has been proposed to remove the amino acids corresponding to indels from structures before calculating distances (17) or to normalize distances (82, 83). A key question is whether to include indels or leave them out, as most studies based on sequence comparison do. There is no easy answer to this question. If indels result from sequencing, assembly, or annotation errors, including them will lead to an overestimation of evolutionary distances. In this case, omitting the atoms of the incriminating amino acids can easily overcome this problem. On the other hand, if they are the result of true evolutionary events, ignoring them will lead to an underestimation of the evolutionary distances between proteins.

We observe also that not all the atoms in the structures carry the same information. While the use of Cα atoms was sufficient to capture a strong phylogenetic signal in structures, using N, O, and C atoms of side chains atoms turned out to be less efficient. The extent to which the signal carried by the atoms in the amino acid side chains is noisy or informative will require further investigations. For example, it has been shown that environmental factors, such as temperature or salinity, exert very strong constraints on proteins, resulting in variations in the frequency of each type of amino acid in protein sequences (84–88). We expect these constraints to be associated with specific signatures in protein structures that can be revealed by PH.

Refining the algorithms underlying the PH method represents our next goal. Although filtration methods and PH-distances used in this study can capture the phylogenetic information contained in protein structures, they were not specifically designed to analyze this type of information. In particular, they do not incorporate information about how proteins evolve, as do models used to calculate evolutionary distances between sequences. Filling this gap will require filtration algorithms that consider additional information such as the nature and the physicochemical features of amino acids, which represents a promising direction for the future development of topological analysis in phylogenetics. Finally, another line of research is exploring the potential contribution of approaches combining PH with machine learning to study protein structure evolution, as PH has been shown to significantly improve classical machine learning (89, 90). Indeed, PH-descriptors provide vectorizations of the complex geometry of protein structures, facilitating the application of learning methods. The application of such approaches has proven to be very efficient for protein classification (60) and protein engineering (91). We show here that PH-descriptors can be used to capture the phylogenetic signal, paving the way for the development of new approaches to study the evolution of protein based on their geometric structure.

## Materials and methods

**Fig. 5.**
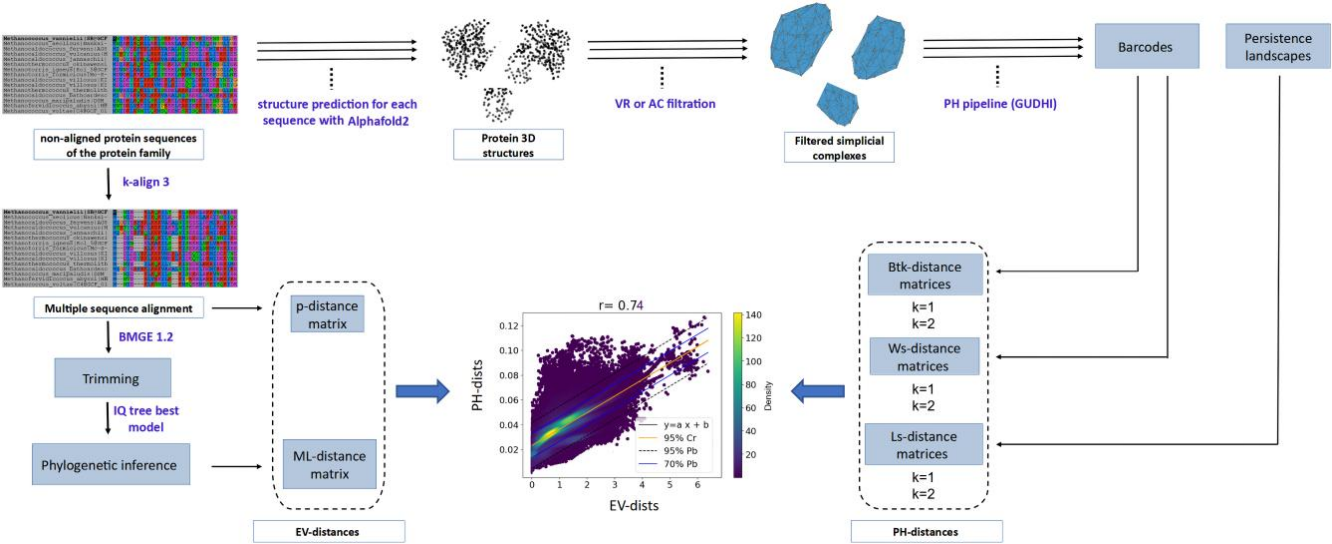
Pipeline describing the steps for the comparison of EV- and PH-distances on each of the 518 protein families. We start by predicting the structure of each sequence in the protein family using Alphafold2. Atoms coordinates constitute the PC on which we apply VR or AC filtration to obtain filtered simplicial complexes. By computing the PH of each obtained filtered simplicial complexes, we generate four PH-descriptors (two barcodes and two persistence landscapes) in homological dimension 1 and 2 (*k* = 1 and *k* = 2, respectively). For each pair of structures, the barcodes are compared using Btk-distances and Ws-distances in each dimension, and the persistence landscapes are compared using Ls-distances. This generated six PH-distance matrices. On the other hand, we align the protein family and compute the p-distance matrix between each pair of sequences. After trimming the multiple sequence alignment, we infer the phylogenetic tree and compute from it the ML-distance matrix. Each EV-distance matrix is then compared to each PH-distance matrix.

A detail scheme of the pipeline used is shown as Fig. 5.

### Dataset assembly

The study has been conducted on seven bacterial and three archaeal major taxa: *Bacillales* (Firmicutes), *Bacteroidales* (CFB group bacteria), *Corynebacterales* (*Actinomycetota*), *Enterobacterales* and *Pseudomonadales* (*Gammaproteobacteria*), *Hyphomicrobiales* (*Alphaproteobacteria*), *Escherichia* (*Enterobacterales*), *Methanococcales* (*Euryarchaeotes*), *Thermococcales* (*Euryarchaeotes*), and *Sulfolobales* (*Crenarchaeotes*). Reference and representative proteomes of these groups were retrieved from the RefSeq database at the National Center for Biotechnology Information (92). When the number of available proteomes was high for a given taxon, a subsampling of 100 proteomes representative of its diversity has been performed (Table S3).

For each taxon, experimentally resolved structures of monomeric proteins were extracted from the RCSB PDB (93). To ensure the quality of structural data, we considered protein with structures resolution <1.5 Å for bacteria and <2 Å for archaea (see Supplementary material text). We did not perform an exhaustive analysis of all structures available in the PDB, as our aim was to analyze a significant but reasonable number of protein families with at least one experimentally well resolved protein 3D structure in the PDB. For Archaea, due to their limited number, we considered all structures with a resolution below 2 Å. Applying such a criterion to the seven studied bacterial groups would have resulted in a huge number of proteins, and thus protein families, requiring a huge (and perhaps unnecessary) number of 3D structure predictions. That's why we only considered the structures with the best resolution. To avoid redundancy, when several structures were available for a given protein, the one with the best resolution was kept.

For each protein, the corresponding family of homologous proteins was assembled as follows. Homologs were identified in members of the taxon to which the protein belongs using the BLASTP program (94). Alignments with e-value <10^−3^, amino acid identity >0.3, query and subject coverages >0.8, and gap content <0.1 were considered as significant, and the corresponding sequences were retrieved. Protein families containing <7 homologous sequences were discarded.

The final dataset represents 518 protein families, corresponding to 22,940 protein sequences and 763,648 pairs of homologous sequences (Tables S4–S13).

### Protein 3D structure modeling

The structures of the 22,940 proteins were computed with Alphafold2 using the NMRbox (19). We retained predicted models with the highest quality score.

### Point cloud filtration

For each modeled structure, we defined five PCs corresponding to the 3D coordinates of amino acid atoms: alpha carbon (PC(Cα)), all atoms (PC(All-Atoms)), all carbon (PC(All-C)), all nitrogen (PC(All-N)), and all oxygen (PC(All-O)). The Vietoris-Rips (VR) and Alpha Complex (AC) filtration algorithms, and PH-descriptors (i.e. barcodes and persistent landscapes) in homological dimensions 1 and 2 (*k* = 1 and *k* = 2, respectively), were computed using the GUDHI library (95). We have not considered PH-descriptors in homological dimension 0, as they reflect mainly the distances between points and not the intrinsic geometrical properties of PCs.

### Phylogenetic inference and computation of evolutionary distances

For each protein family, an accurate multiple alignment was built using MAFFT v7.453 with the L-INSI option (96) and trimmed using BMGE v2 with the BLOSUM45 substitution matrix (97). A maximum likelihood (ML) phylogenetic tree was inferred using IQ-TREE v2.1.2 (98) and the best fitted-model according to ModelFinder (99). The tree branch robustness was estimated using the ultrafast bootstrap procedure implemented in IQ-TREE (1,000 replicates). For each pair of sequences, EV-distances were computed using SEAVIEW v5.0.5 (100). More precisely, observed distances (*p*-distances) were computed from the untrimmed multiple alignments, while ML-distances correspond to patristic distances computed from the ML-trees.

### PH-distance definitions and computation

For each protein family, we calculate a matrix of PH-distances between pairs of homologous protein structures. The PH-distances between two structures are calculated from the associated barcodes in homological dimensions 1 and 2 (*k* = 1 and *k* = 2) (see Supplementary material text for more details). These distances are defined as follows:

The bottleneck distance (Btk1, Btk2) between two barcodes *P* and *Q* is defined by


$$\mathrm{Btk}(P,Q)=\underset{\varphi :P\to Q}{\inf}\;\underset{a\in P}{\sup}\;\|a-\varphi (a){\|}_{\infty },\;\;\;\;$$

where φ ranges over all bijections between *P* and *Q* and *a* is an interval in *P*.

The Wasserstein distance (Ws1, Ws2) between two barcodes *P* and *Q* is defined by


$$\mathrm{Ws}(P,Q)=\underset{\varphi :P\to Q}{\inf}\;\sqrt{\underset{a\;\in \;P}{\sum }\;\|a-\varphi (a){\|}_{2}^{2}},$$

where the infimum is taken over all bijections φ between *P* and *Q*.

The Landscape distance (Ls1, Ls2) between two persistence landscapes $\lambda ,\lambda^{\prime }$ is given by the $L^{2}$ -norm:


$$\mathrm{Ls}(\lambda ,\lambda^{\prime })=\sqrt{\overset{\infty }{\underset{\ell =1}{\sum }}\;\int {\left|\lambda_{\ell }(t)-{\lambda \prime }_{\ell }(t)\right|}^{2}dt}.\;\;\;\;$$

The Btk1, Btk2, Ws1, and Ws2 distances were computed using the GUDHI library (95), while Landscape distances (Ls1, Ls2) were computed using Pysistence (61). We used *p* = 2 for Ls1 and Ls2 and p = q = 2 for Ws1 and Ws2 (see Supplementary material text). The Pearson's coefficient correlation was used to calculate the correlation between EV-distances and PH-distances.

To go further, we tested the impact of three protein features on PH-distances: (i) the length of the proteins, (ii) the amount of indels in protein sequences, and (iii) and the quality of predicted structures. Regarding protein length, to enable comparisons between different datasets, phylogenetic distances computed from molecular sequences are usually normalized by the number of compared residues (i.e. amino acid or nucleotide sites) and expressed in unit of expected number of substitutions per site. This allows to compare distances computed from protein of different lengths. In contrast, PH-distances are not normalized, which precludes comparisons of PH-distances computed from PCs with different number of points, and thus of structures with different number of amino acids/atoms. To overcome this issue, we normalized PH-distances by the average number of points of the two compared PCs (see Supplementary material text). Regarding indels, starting from the multiple sequences alignments, we identified the amino acids involved in indels for each pair of proteins. Their atoms were omitted for the calculation of PH-distances from PCs. Therefore, for each structure, the number of atoms considered varies depending on the structure it is compared to (see Supplementary material text). Finally, to measure the impact of structure prediction quality, we masked points corresponding to amino acids having an index lower than 70% in PCs (see Supplementary material text).

## Supplementary Material

pgae158_Supplementary_Data

## Data Availability

Protein sequences, multiple alignments, ML trees, and 3D protein structure predictions were deposited in the permanent Mendeley data repository [https://data.mendeley.com/datasets/mhpcr6c827/1] (DOI 10.17632/mhpcr6c827.1) (101).
