# Supplementary material for: Persistent homology reveals strong phylogenetic signal in 3D protein structures
Source: PNAS Nexus. 2024 Apr 17;3(4):pgae158. doi: 10.1093/pnasnexus/pgae158 (PMC11058471; doi:10.1093/pnasnexus/pgae158)
Supplement: pgae158_Supplementary_Data [file pgae158_supplementary_data.pdf]

Supporting Information for

Persistent homology reveals strong phylogenetic signal in three-dimensional protein structures

Léa Bou Dagher[1,2,3], Dominique Madern[4], Philippe Malbos[2*], Céline Brochier-Armanet[1*]

[1]Université Claude Bernard Lyon 1, CNRS, VetAgro Sup, Laboratoire de Biométrie et Biologie Évolutive, UMR5558, Villeurbanne, France

[2] Université Claude Bernard Lyon 1, CNRS, Institut Camille Jordan, UMR5208, Villeurbanne, France

[3] Université Libanaise, Laboratoire de mathématiques, École Doctorale en Science et Technologie, PO BOX 5, Campus Rafik Hariri, Hadath, Liban

[4] Univ. Grenoble Alpes, CEA, CNRS, IBS, 38000, Grenoble, France

***Corresponding authors**: celine.brochier-armanet@univ-lyon1.fr, malbos@math.univ-lyon1.fr

Email: celine.brochier-armanet@univ-lyon1.fr, malbos@math.univ-lyon1.fr

This PDF file includes:

Supporting Information text

Figures S1 to S28

Tables S1 to S13

SI References

Table of contents

Supporting Information Text 4
1. An overview on Persistent Homology 4
Persistent Homology for Topological Data Analysis 4
PH pipeline for topological analysis of a point cloud 4
Simplices and filtered complexes 5
Filtrations of a point cloud 5
Vietoris-Rips filtration 5
Voronoi diagram. 6
Alpha Complex 6
Persistence landscapes 6
PH programs 7
2. Persistent homology distances and normalization 8
Norms on barcodes 8
PH-distances 8
Normalization of PH-distances 9
PH-distances and indels 9
PH-distances and confidence indices 9
Stability of PH-distances 9
Supplementary Figures 11
Figure S1. Example of a barcode and its persistence landscape. 12
Figure S2. The three main steps of the PH pipeline for topological analysis of a point cloud. 13
Figure S3. Examples of simplices and simplicial complexes 14
Figure S4. Example of Vietoris-Rips (VR) filtration and construction of its persistence barcode. 15
Figure S5. Example of Alpha Complex (AC) filtration and construction of its persistence barcode. 16
Figure S6. The Voronoi diagram of 20 points in the plane. 17
Figure S7. Barcode, peak function, and persistence landscape. 18
Figure S8. Boxplot showing the distribution of RMSD between experimentally resolved protein structures from the RCSB PDB database and Alphafold2 predicted structures. 19
Figure S9. Histogram showing the distribution of 5,666,844 Cα confidence indices (CI) of the 22,940 protein structures predicted with Alphafold2 20
Figure S10. Histogram showing the distribution of average Cα confidence indices (CI) of protein structures predicted with Alphafold2 21
Figure S11. Boxplots showing the distribution of normalized PH-distances calculated between pairs of homologous proteins using PC(Cα) from Alphafold2 structure predictions and the VR filtration 22
Figure S12. Boxplots showing the distribution of normalized PH-distances calculated between pairs of homologous proteins using PC(Cα) from Alphafold2 structure predictions and the AC filtration 23
Figure S13. Boxplots showing the distribution of normalized PH-distances calculated between pairs of homologous proteins and pairs of non-homologous proteins using PC(Cα) from Alphafold2 structure predictions and the AC filtration 24
Figure S14. Boxplots showing the distribution of correlation coefficients on the 518 protein families between ML- and Ws-distance in homological dimensions 1 (Ws1) and 2 (Ws2) 25
Figure S15. Correlation plots between ML-distances and normalized Ws-distances in homological dimensions 1 and 2 for the P9WNX1 protein family 26

Figure S16. Trimmed multiple alignment of the P9WNX1 protein family 28
Figure S17. Correlation plots between ML-distances and normalized Ws-distances in homological dimensions 1 and 2 for the P58502 protein family 29
Figure S18. Trimmed multiple alignment of the P58502 protein family 31
Figure S19. Correlation plots between ML-distances and normalized Ws-distances in homological dimensions 1 and 2 for the Q9HXX5 protein family 32
Figure S20. Trimmed multiple alignment of the Q9HXX5 protein family 34
Figure S21. Effect of indels on correlations between ML-distances and normalized WS-distances 35
Figure S22. Effect of the removal indels on correlations between ML-distances and normalized WS-distances 36
Figure S23. Boxplots showing the distribution of ML-distances for each of the ten taxa analyzed 37
Figure S24. Boxplots showing the distribution of normalized Ws-distances in homological dimension 1 for each of the ten taxa analyzed 38
Figure S25. Boxplots showing the distribution of normalized Ws-distances in homological dimension 2 for each of the ten taxa analyzed 39
Figure S26. Histogram showing the variation in the number of nitrogen atoms in the side chain of the 20 amino acids 40
Figure S27. Histogram showing the variation in the number of oxygen atoms in the side chain of the 20 amino acids 41
Figure S28. Histogram showing the variation in the number of carbon atoms in the side chain of 20 amino acids 42
Supplementary Tables 43
Table S1. Table showing the correlation coefficients between PH-distances and the average number of points in PC(Cα) being compared 44
Table S2. Table showing the correlation coefficients between EV- and normalized PH-distances computed from PC(Cα) 45
Table S3. List of the ten prokaryote taxonomic groups used in this work 46
Table S4. List of the 63 protein families from Bacillales 73
Table S5. List of the 31 protein families from Bacteroidales 75
Table S6. List of the 56 protein families from Corynebacteriales 76
Table S7. List of the 102 protein families from Enterobacterales 78
Table S8. List of the 85 protein families from Escherichia 81
Table S9. List of the 21 protein families from Hyphomicrobiales 84
Table S10. List of the 27 protein families from Methanococcales 85
Table S11. List of the 39 protein families from Pseudomonadales 86
Table S12. List of the 29 protein families from Sulfolobales 87
Table S13. List of the 65 protein families from Thermococcales 88
Bibliography 90

Supporting Information Text

1. An overview on Persistent Homology

We recall here the main concepts on persistent homology. For comprehensive introductory presentations see also (1-4). We refer the reader who would like to know more about the notions of simplicial complexes, homological constructions, and general notions of algebraic and computational topology to (5, 6).

Persistent Homology for Topological Data Analysis

Topological data analysis (TDA) aims to analyze the shape of datasets by providing mathematical, statistical, and algorithmic methods, based on the principles of algebraic topology to infer geometric structures underlying these sets. Data, often represented as point clouds in Euclidean metric spaces, are of very different natures, voluminous, high-dimensional, and noisy with missing or incorrect information. TDA provides robust methods that preserve isometry and scale invariance for identifying structures in such data.

Persistent homology (PH) is a homological theory that provides efficient algorithms for TDA. This approach emerged simultaneously in several works in the late 1990s (7-11).

Consider a point cloud (PC) in a Euclidean space from which we would like to extract geometric properties, such as the presence of cycles, cavities or other higher-dimensional properties. The aim is to describe these features by homological invariants in each dimension: connected components (homological dimension k=0), cycles (homological dimension k=1), cavities (homological dimension k=2) … The data are discrete, so we do not have direct access to the geometric information with classical homological methods (e.g., simplicial homology) based on the topology of the ambient space.

The main idea of PH is to construct an intermediate combinatorial object from the PC, namely an increasing sequence of simplicial complexes, that encodes the geometric structure of the PC through a filtration. Filtration is calculated from the evolution of certain parameters in order to generate the persistence of geometric features, such as the number of connected components, cycles, cavities… The persistence of these features is characterized by their birth and death throughout the filtration process. These changes are encoded in homological invariants, that we call PH-descriptors, such as barcodes and persistence landscapes.

A barcode is a multi-set of intervals $[b, d]$, where b is the birth value of a topological feature and d its death value. A persistence landscape is a vectorization of a barcode, see Figure S1.

An interval in the barcode corresponds in the persistence landscape to a peak of height $(d - b) / 2$ supported on the interval $[b, d]$. The life duration of a topological characteristic is measured by $d - b$. Topological features with small life duration are referred to as noise. Noise appears in barcode as short intervals and in persistence landscapes as very low peaks. Moreover, PH makes it possible to compare data sets, by comparing their generated topological summaries with different distances.

PH methods have found many fields of application, in shape recognition (12, 13), physical science (14-17), molecular biology (18, 19), biomedicine (20-23), life science (24, 25). Regarding structural biology, PH has been used to classify protein structures, recognize conformations (26, 27), analyze folding dynamics (28), study physical and chemical protein properties (29) and (26 , 27, 28, 29 , 30).

PH pipeline for topological analysis of a point cloud

The pipeline takes as input the PC representing the data under study. An increasing sequence Filt(PC) of simplicial complexes whose vertices are the points in PC, is constructed using a filtration method, see Figure S2. The filtration process generates a persistence module PersMod(PC), i.e., a family of finite dimensional vector spaces generated by the simplicial complexes of Filt(PC) together with a family of linear maps that encode inclusions of simplicial complexes. A persistence module has an algebraic signature, called a

barcode, which characterizes it up to isomorphism. We show that the barcode of the module PersMod(PC) describes the evolution of the multidimensional topological characteristics of the point cloud PC throughout the filtration Filt(PC). All these steps form the PH pipeline. For detailed account of the mathematical background to this method see (1, 5).

Simplices and filtered complexes

Simplicial complexes can be seen as higher-dimensional generalization of graphs. A simplicial complex is a geometric object determined by combinatorial data, describing certain topological spaces by generalizing the notion of surface triangulation. The building blocks of a simplicial complex are simplices. Geometrically, a 0-simplex is a vertex, a 1-simplex is an edge, a 2-simplex is a triangle, a 3-simplex is a tetrahedron and so on, as pictured on Figure S3.

We can define a simplicial complex roughly as a union of simplices, but these simplices are glued under certain conditions. Explicitly, we define a simplicial complex K as a finite collection of simplices such that:

i) Every face of a simplex in K also belongs to K;

ii) The intersection of any two simplices in K is either an empty or common face of the two simplices.

Filtrations of a point cloud

A subset $L \subset K$ of simplices is called a subcomplex of K if, for each simplex σ in L, any face τ of σ in K is also in L. A filtered simplicial complex K build on top of a dataset X is a nested sequence $(K_i)_{0\leq i\leq n}$ of subcomplexes of K:

$$X = K_0 \subseteq K_1 \subseteq \cdots \subseteq K_n = K,$$

with vertex set X.

In this work, we consider two filtration methods: the Vietoris-Rips (VR) and the Alpha Complex (AC) filtrations. The aim is to build a family of simplicial complexes from a PC in a Euclidean space. For each point, we consider a ball centered at that point, whose radius is gradually increased. We then study the configuration of all these balls. Simplexes are then added as the balls intersect with the growth of their radius. This creates a filtration process where the earlier simplicial complex is included in the latter one. This process continues until the balls fill the entire structure when the value of the radius becomes large enough. During the filtration, as the radius increases, the structure of the overlapping balls changes, and larger holes are expected to persist for a more extended period than smaller ones. Therefore, more noticeable features are expected to persist for longer periods as the radius increases, while noisy features disappear rapidly. These persistence durations are encoded in barcodes as illustrated in Figures S4 and S5.

Vietoris-Rips filtration

Recall that a metric space is a set of points together with a metric on the set. The metric is a function that defines a concept of distance between any two points of the set. Explicitly, a metric space (M, d) is a space M with a distance function $d: M \times M \to \mathbb{R}$ satisfying the three following properties, for all $x, y, z \in M$,

i) Identity: $d(x, y) = 0$ if, and only if, $x = y$,

ii) Symmetry: $d(x, y) = d(y, x)$,

iii) Triangular inequality: $d(x, z) \leq d(x, y) + d(y, z)$.

The metric space is finite when it has a finite number of points. The VR filtration of a finite metric space M is an increasing family of simplicial complexes $\left(VR_{\epsilon_i}(M)\right)_{0\leq i\leq n}$ indexed by real numbers $\epsilon_i \geq 0$, and defined as follows. A simplex σ belongs to $VR_{\epsilon_i}(M)$ if the diameter of σ, *i.e.*, the maximum distance between the vertices of σ, is less than or equal to ϵ_i.

For every $\epsilon_i \in \mathbb{R}$, $VR_{\epsilon_i}(M)$ is a simplicial complex whose vertices are the elements of M. Moreover, for $\epsilon_i \leq \epsilon_j$, $VR_{\epsilon_i}(M)$ is a subcomplex of $VR_{\epsilon_j}(M)$.

The VR filtration algorithm is defined as follows:

- ***i)*** For a given PC in a metric space, set a maximum radius value, denoted by ϵ_{max},
- ***ii)*** Initialize the VR filtration at radius value $\epsilon = 0$ which consists of the points of the PC,
- ***iii)*** Incrementally increase the radius value ϵ from 0 to ϵ_{max} and the VR filtration is obtained by adding a new simplex σ if the pairwise distance between its vertices is less than or equal ϵ,
- ***iv)*** Continue incrementing the radius value and adding simplices until $\epsilon = \epsilon_{max}$.

The VR filtration is constructed in this way. An example is shown on Figure S4. Its vertices are the points of the PC, and its edges and higher-dimensional simplices correspond to the subsets of points that are connected by edges or higher-dimensional simplices that satisfy the pairwise distance condition described above.

Voronoi diagram.

Given a set X of points of a metric space (M, d). The Voronoi diagram of X is a geometric structure that divides the space into regions, called Voronoi cells (Figure S6). The Voronoi cell of a point p in X is the set of points for which p is the closest:

$$V_p = \{x \in X \mid d(x,p) \leq d(x,q), \text{for all } q \in X\}.$$

Alpha Complex

The alpha complex filtration (AC filtration) is a generalization of the concepts of convex hull and Delaunay triangulation. On a finite metric space (M, d) it is defined as an increasing family of simplicial complexes $\left(A_{\alpha_i}(M)\right)_{0 \leq i \leq n}$, indexed by real numbers $\alpha_i \geq 0$, and constructed as follows. Denote by $B(p, \alpha_i)$ the closed ball centered at a point p with radius α_i. A simplex σ belongs to $A_{\alpha_i}(M)$ if the intersection between $B(p, \alpha_i)$ and the Voronoi cell of p, for every p in σ, is non-empty, that is

$$\bigcap_{p \in \sigma} B(p, \alpha_i) \cap V_p \neq \emptyset.$$

For every $\alpha_i \in \mathbb{R}$, $A_{\alpha_i}(M)$ is a simplicial complex whose vertices are the elements of M. Moreover, for $\alpha_i \leq \alpha_j$, $A_{\alpha_i}(M)$ is a subcomplex of $A_{\alpha_j}(M)$.

Note that, not all the simplices are included in the final AC filtration, contrary to the VR complex. Only the ones that are included within a specified alpha radius are included in the final complex.

The AC filtration algorithm is defined as follows:

- ***i)*** For a given PC in a metric space, compute its Voronoi diagram,
- ***ii)*** Initialize the AC filtration at radius value $\alpha = 0$ which consists of the points of the PC,
- ***iii)*** Incrementally increase the radius value α and the AC filtration at a given radius value α is obtained by adding a new simplex σ when the intersection between the closed ball centered at p with radius α_i and the Voronoi cell of p, for every p in σ is non-empty,
- ***iv)*** Continue by adding simplices in order of increasing alpha radii until all possible simplices have been added and the final AC filtration is reached.

The resulting AC filtration is a subset of the Delaunay triangulation. An example is shown on Figure S5. Its vertices are the points in the PC, and its edges and higher-dimensional simplices correspond to the edges and higher-dimensional simplices of the Delaunay triangulation that satisfy the circumradius condition described above.

Persistence landscapes

Introduced by Bubenik in 2015, persistence landscapes are vectorizations of barcodes which enable the use of statistical tools (11), see also Figure S7. The persistence landscape of a barcode is built by assigning to each interval $I = [b, d]$ of the barcode the following peak function:

$$f_I(t) = \begin{cases} 0 & \text{if } t \notin [b, d]; \\ x - b & \text{if } t \in \left[b, \dfrac{b+d}{2}\right]; \\ -x + d & \text{if } t \in \left[\dfrac{b+d}{2}, d\right]. \end{cases}$$

We then define the persistence landscape as the function $\lambda: \mathbb{N} \times \mathbb{R} \to \mathbb{R}$ where $\lambda(\ell, t)$ is the ℓ-th largest value of $f_I(t)$ over each interval I in the barcode. Equivalently, a persistence landscape is defined by a sequence of functions $\lambda_\ell: \mathbb{R} \to \mathbb{R}$ given by $\lambda_\ell(t) = \lambda(\ell, t)$.

PH programs

To efficiently analyze and visualize PH, various software tools have been developed (31). These software packages offer a range of functionalities, from pre-processing and data import to barcode visualization and distance calculation. Some popular software for PH includes Ripser (32), Dionysus (33), and GUDHI (34). This latter, used in this work, is an open-source software library designed for TDA and computational geometry, available in C++ and Python interfaces. PH methods in this software take as input point clouds or distance matrices. VR, AC and other filtrations methods are available in GUDHI.

2. Persistent homology distances and normalization

We explain here the notions of persistent homology distance, PH-distance for short. These distances are constructed from barcodes and persistence landscapes using different norms. First, we recall the definitions of the used norms. We then present the definitions of bottleneck distance, Wasserstein distance and landscape distance, which are the three distances considered in this study.

Norms on barcodes

Recall that a barcode is a multi-set of intervals $[b, d]$, where $b,\ d \in (\mathbb{R} \cup +\infty)$ and b is the birth value of a topological feature and d its death value. Let $[b_1, d_1], [b_2, d_2]$ where $b_1, b_2, d_1, d_2 \in (\mathbb{R} \cup +\infty)$, be two intervals in a barcode.

Infinity norm. The infinity norm on the intervals is given by:

$$\|[b_1, d_1] - [b_2, d_2]\|_\infty \coloneqq \max\{|b_1 - b_2|, |d_1 - d_2|\},$$

where $|\cdot|$ denotes the absolute value with the convention that:

- ***i)*** If $d_1 = d_2 = +\infty$, then $|d_1 - d_2| \coloneqq 0$.
- ***ii)*** If one, and only one of d_1, d_2 is $+\infty$, then $|d_1 - d_2| \coloneqq +\infty$.

P-norm. The p-norm on the intervals is given by

$$\|[b_1, d_1] - [b_2, d_2]\|_p \ = \ (|b_1 - b_2|^p + |d_1 - d_2|^p)^{1/p}.$$

PH-distances

To compute the distance between barcodes, one can compare the pairwise matched intervals. There are multiple ways to accomplish this depending on the considered norms to measure the difference between barcodes.

<u>Bottleneck distance</u>.

The notion of bottleneck distance was introduced in (35). Let P and Q be two barcodes. Let $a = [b, d]$ be an interval in P. The bottleneck distance between the barcodes P and Q is defined to be

$$\mathrm{Btk}(P, Q) = \inf_{\varphi: P \to Q} \sup_{a \in P} \big\| a - \varphi(a) \big\|_\infty ,$$

where φ ranges over all bijections between P and Q. We say that intervals $[b, d]$ and $\varphi([b, d])$ are paired. Note that if the barcodes P and Q do not contain the same number of intervals, a non-paired interval $[b, d]$ is paired with the interval $\left[\frac{b+d}{2}, \frac{b+d}{2}\right]$.

<u>Wasserstein distance</u>.

Let $q \in [0, +\infty[$, the qth Wasserstein distance between two barcodes P and Q is defined to be

$$Ws_q(P, Q) = \inf_{\varphi: P \to Q} \left(\sum_{a \in P} \big\| a - \varphi(a) \big\|_p^q \right)^{\frac{1}{q}},$$

where the infimum is taken over all bijections between P and Q. Similarly, to the Btk distance, if the barcodes P and Q do not contain the same number of intervals, a non-paired interval $[b, d]$ is paired with the interval $\left[\frac{b+d}{2}, \frac{b+d}{2}\right]$. Note that at the limit when q tends to infinity, the Wasserstein distance is equal to the Bottleneck distance.

A drawback of the bottleneck distance is that it considers only the furthest pair of corresponding intervals. So, the bottleneck distance is the cruder of the two distances, and the Wasserstein distance is more sensitive to details in the barcodes as it takes into consideration all the matched intervals. Indeed, the bottleneck distance takes the maximum of the distances between matched intervals (with the most efficient matching),

while the Wasserstein distance takes the sum of the distances between matched intervals over the best matching.

Landscape distance.

Distance between two persistence landscapes λ, λ' is given by the L^p-norm for $p \geq 1$,

$$Ls_p(\lambda, \lambda') = \left\|\lambda - \lambda'\right\|_p = \left[\sum_{\ell=1}^{\infty} \int |\lambda_\ell(t) - \lambda'_\ell(t)|^p dt\right]^{\frac{1}{p}}.$$

The Landscape distance does not search for the best matching between the sequences of functions. This distance matches the functions in order and calculates the distance between them. Contrary to the Wasserstein distance that searches to the most efficient matching between the barcodes.

Normalization of PH-distances

Let PC_1 and PC_2 be two point clouds containing n and m number of points respectively. Define the average number of PC_1 and PC_2 as $p = (n+m)/2$. The normalized PH-distances between the barcodes P and Q and persistence landscapes λ and λ' of PC_1 and PC_2 respectively are defined by

$$\overline{\mathrm{Btk}}(P,Q) := \frac{\mathrm{Btk}(P,Q)}{p}, \qquad \overline{Ws_q}(P,Q) := \frac{Ws_q(P,Q)}{p}, \qquad \overline{Ls_p}(\lambda,\lambda') := \frac{Ls_p(\lambda,\lambda')}{p}.$$

PH-distances and indels

We consider a protein family multiple alignment A of n homologous protein sequences $S_1, \cdots, S_n$, and we denote PC(S_1), ⋯, PC(S_n) their corresponding point clouds. For all $i < j$, we compare the point clouds PC(S_i) to PC(S_j) by considering indels in the alignment A. Then we remove

- ***i)*** the points in PC(S_i) of amino acids in S_i aligned with gaps in S_j,
- ***ii)*** the points in PC(S_j) of amino acids in S_j aligned with gaps in S_i.

The two resulting point clouds associated to S_i and S_j are denoted by PC_{indel} (S_i; S_j; A) and PC_{indel} (S_j; S_i; A) respectively. By definition, these two point clouds depend on the alignment A.

In this work the calculation of PH-distances involves both normalization by the number of points in PC and the presence of gaps. For example, the Wasserstein distance between two protein sequences S_1, and S_2 with respect to an alignment A is defined as $Ws_q(P,Q)$ where P and Q are barcodes associated to PC_{indel} (S_1; S_2; A) and PC_{indel} (S_2; S_1; A) respectively.

PH-distances and confidence indices

Alphafold2 predicted structures contain atomic coordinates and per-residue confidence indices on a scale from 0 to 100, with higher scores corresponding to higher confidence. This confidence index (CI) corresponds to the model's predicted per-residue scores on the Local Distance Difference Test (lDDT)-Cα metric.

Let A be a protein family multiple alignment of n homologous protein sequences $S_1, S_2, \cdots, S_n$ and PC(S_1), PC(S_2), ⋯, PC(S_n) their corresponding point clouds. For every $i < j$, we compare PC(S_i) to PC(S_j) considering the CI < 70%. This means that for two aligned amino acids in S_i and S_j, one of which has a CI of less than 70%, we remove the two corresponding points from PC(S_i) and PC(S_j). Consequently, the resulting point cloud of S_i, denoted by $PC_{CI>70\%}$ (S_i; S_j; A) depends on the alignment and on S_j. This approach is local, because it considers the PC(S_i) for $1 \leq i \leq n$, with dependency relationships between them.

Note that when we consider the point clouds PC(All-N), PC(All-O), PC(All-C), or PC(All-Atom) we only work on the atoms of amino acids whose Cα has a CI > 70%.

Stability of PH-distances

PH-distances are stable with respect to small variations (noise, dynamic nature of data…) on the coordinates of points in the PC. The PH stability theorem ensures the robustness of PH to noise by stating how the

information retained in PH-descriptors is stable under small variations of the data (35). This result is crucial in practical applications of PH because it guarantees that the geometric features captured by PH are reliable and meaningful even in the presence of noise or small variations in the input.

Supplementary Figures

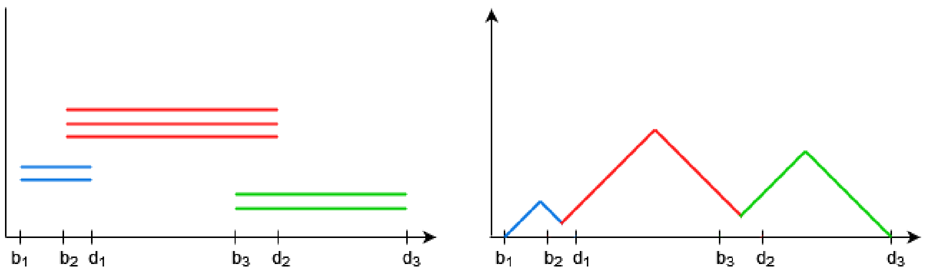

Figure S1. Example of a barcode and its persistence landscape. A barcode containing 7 intervals (left) and its persistence landscape containing one function (right). Here, the barcode consists of (2, $[b_1, d_1]$), (3, $[b_2, d_2]$), and (2, $[b_3, d_3]$) and its persistence landscapes consists of one function.

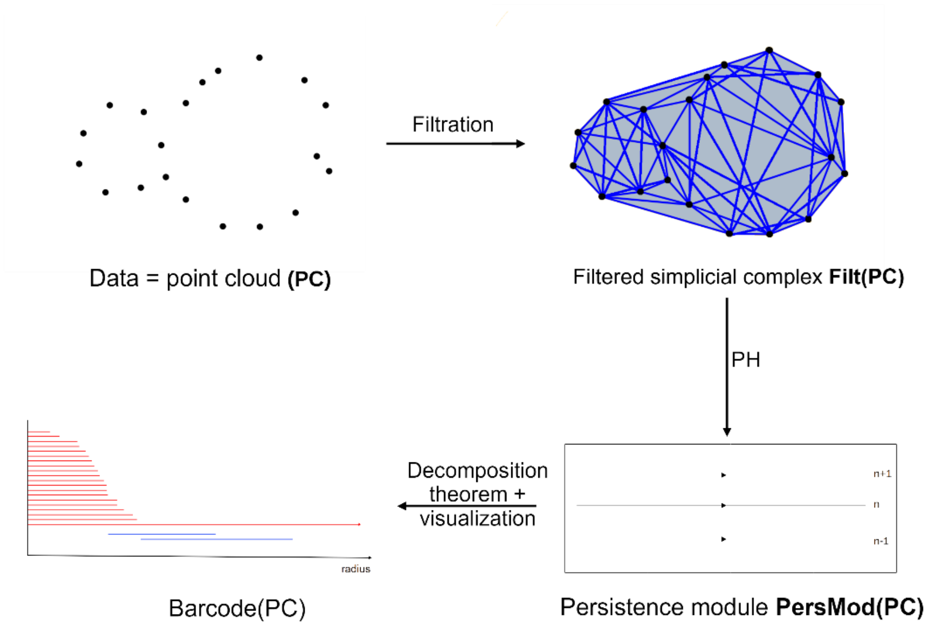

Figure S2. The three main steps of the PH pipeline for topological analysis of a point cloud. For more explanation, see Supporting information text.

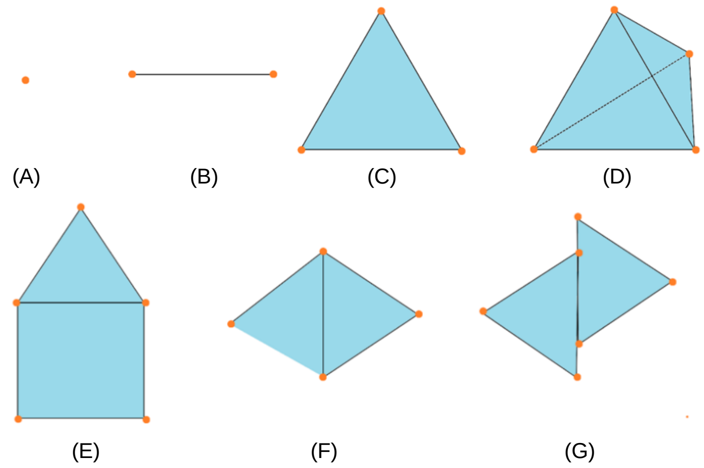

Figure S3. Examples of simplices and simplicial complexes. **(A)** to **(D)** are respectively 0- 1- 2-, and 3-simplices. **(E)** is a simplicial complex. **(F)** and **(G)** are not simplicial complexes since there is a missing edge in (F) and two triangles meet along an edge which is not an edge of either triangle in (G).

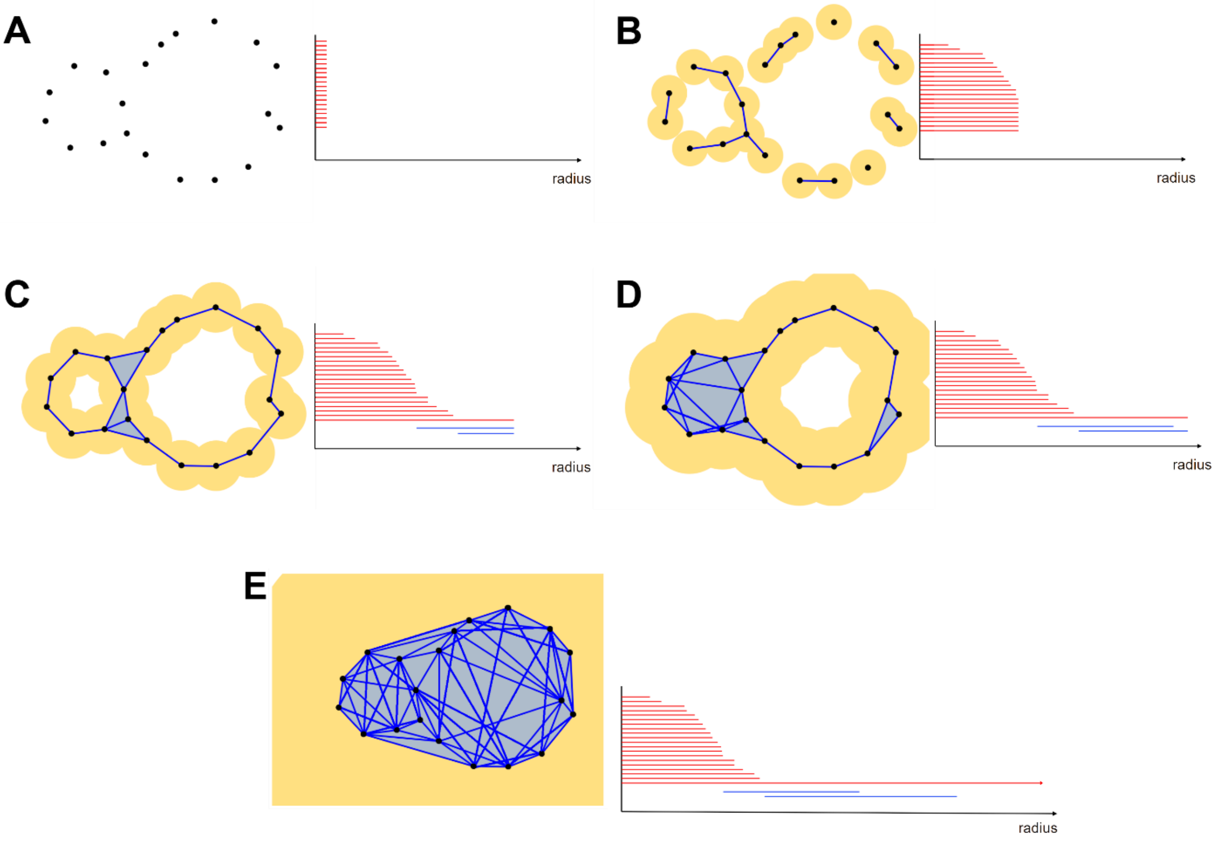

Figure S4. Example of Vietoris-Rips (VR) filtration and construction of its persistence barcode. Red bars (resp. blue bars) represent 0-dimensional (resp.1-dimensional) PH. On X we construct the VR filtration and generate the barcode, by taking a ball centered at each ball with radius ϵ. **(A)** For $\epsilon = 0$, the union of balls corresponds to the points of X, each of them is a 0-dimensional feature known as connected component. Each connected component is represented by the birth of an interval in the barcode at $\epsilon = 0$. The PC contains 20 points so there are 20 connected components represented by 20 intervals at $\epsilon = 0$ **(B)** As the radius increases, some balls overlap, new simplices are added to the simplicial complex resulting in the death of come connected components. The barcode records these deaths by assigning end points to the corresponding intervals. At this stage there are 8 connected components represented by 8 persistent intervals. **(C)** Additional components are connected, contributing to a single connected component and, so, all the intervals corresponding to a 0-dimensional feature have been ended, except the one associated with the never-ending connected component. Furthermore, two 1-dimensional features known as cycles have appeared, resulting in the birth of two new intervals (in blue) starting at their birth scale. **(D)** The smaller of the two cycles has been filled, resulting in its death in the filtration and the end of the corresponding blue interval. **(E)** The second cycle also dies as the radius increases; only the long (and never dying) red interval remains. So, the barcode is seen as a multiscale topological signature encoding the homology of the union of balls for all radii, revealing the 8-shape underlying the PC: one persistent connected component, two cycles one bigger with greater persistence duration than the other.

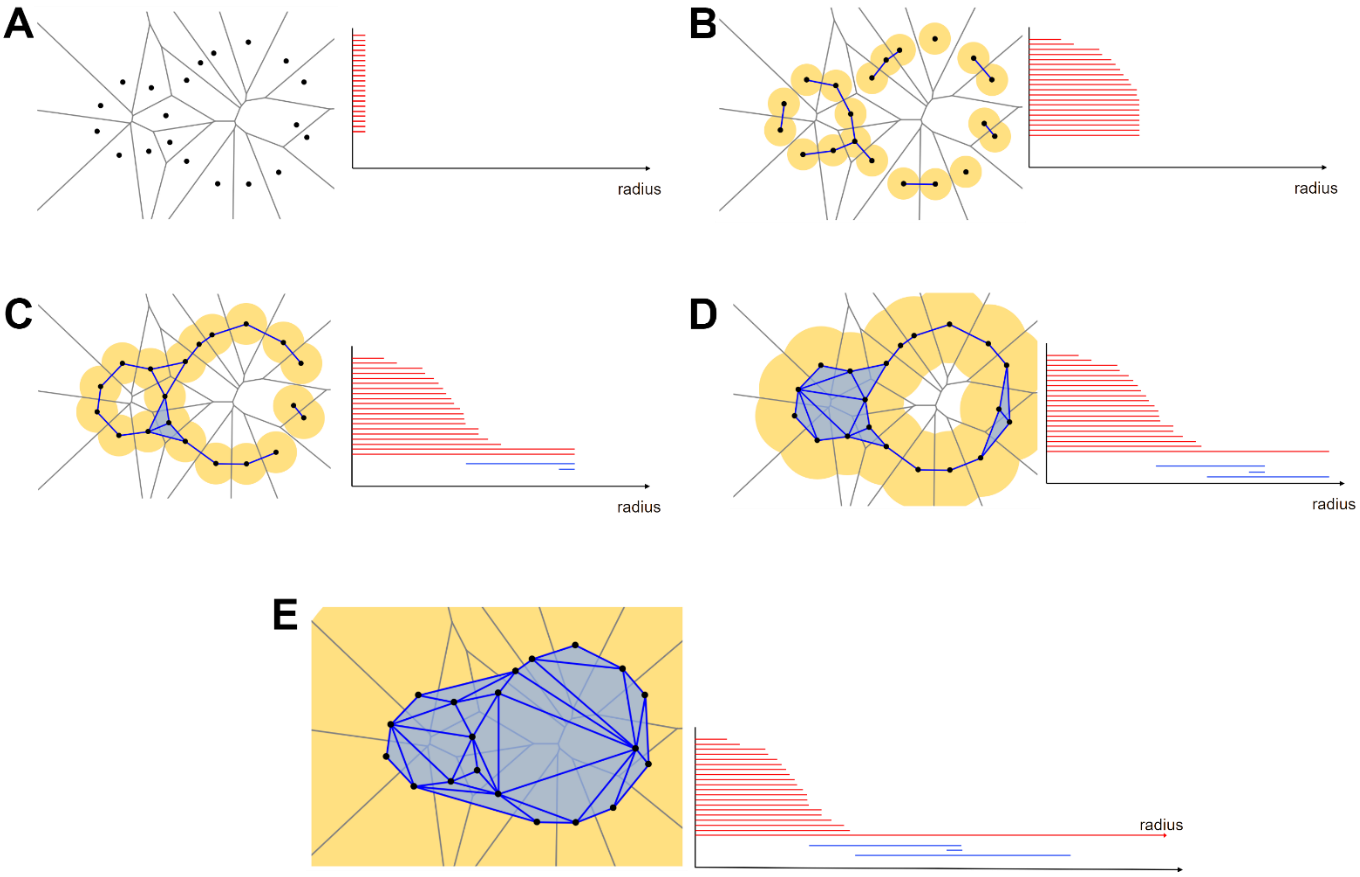

Figure S5. Example of Alpha Complex (AC) filtration and construction of its persistence barcode. Red bars (resp. blue bars) represent 0-dimensional (resp.1-dimensional) persistent homology. On the PC X, we construct the Delaunay triangulation and the AC filtration and generate the barcode, by taking a ball centered at each ball. We add a simplex when the intersection between the ball and a Delaunay cell is non-empty **(A)** For the radius $\epsilon = 0$, the union of balls is reduced to the initial finite set of points, each of them corresponding to connected components; an interval is created for the birth for each of these features at $r = 0$. **(B)** Some balls start to overlap, resulting in the death of some connected components that get merged; the barcode keeps track of these deaths, putting an end point to the corresponding intervals as they disappear. **(C)** New components have merged, giving rise to a single connected component and, so, all the intervals associated with a 0-dimensional feature have been ended, except the one corresponding to the remaining components; two new 1-dimensional features have appeared, resulting in two new intervals (in blue) starting on their birth scale. **(D)** One of the two 1-dimensional cycles has been filled, resulting in its death in the filtration and the end of the corresponding blue interval. **(E)** All the 1-dimensional features have died; only the long (and never dying) red interval remains. So, the barcode is seen as a multiscale topological signature encoding the homology of the union of balls for all radii as well as its evolution across the values of ϵ.

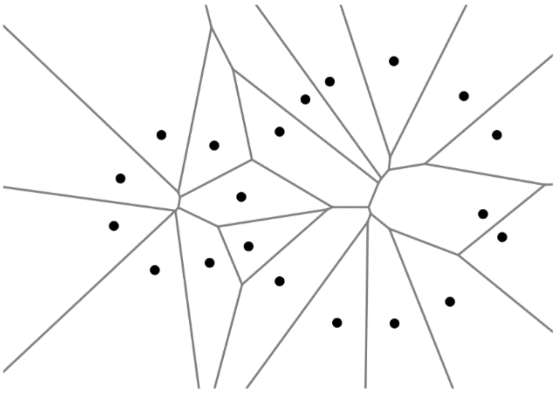

Figure S6. The Voronoi diagram of 20 points in the plane. Each vertex in the diagram is equally far from the points that constitute the Voronoi cells and further from all other points in X.

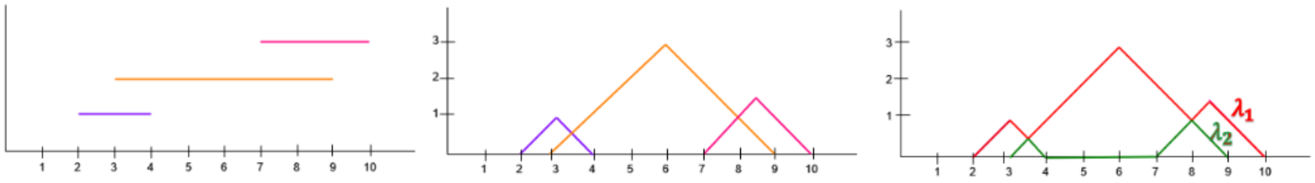

Figure S7. Barcode, peak function, and persistence landscape. A barcode (left), the peak function associated to each interval (middle) and the corresponding persistence landscape $\lambda = (\lambda_1, \lambda_2)$ (right).

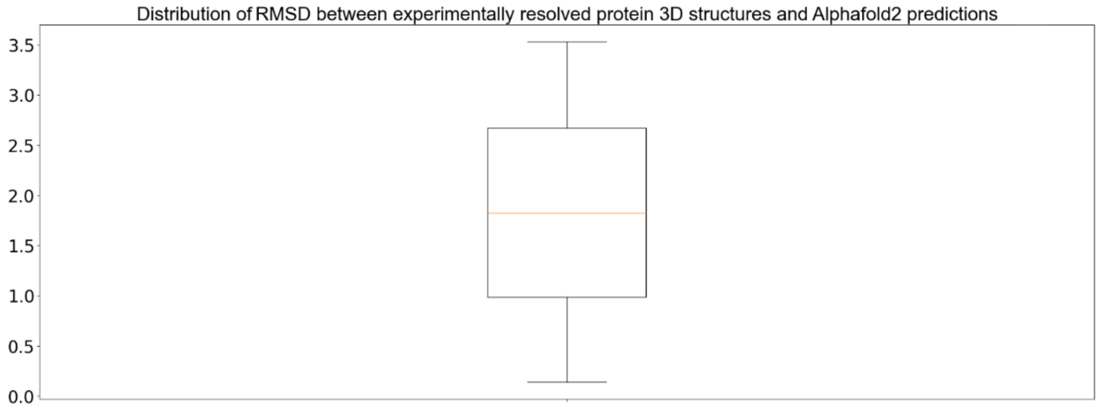

Boxplot	Lower whisker	Lower quartile	Median	Upper quartile	Upper whisker
Alphafold2	0.14	0.99	1.83	2.67	3.53

Figure S8. Boxplot showing the distribution of RMSD between experimentally resolved protein structures from the RCSB PDB database and Alphafold2 predicted structures. The y-axis corresponds to RMSD in Å (22,940 values). Metrics for the distribution are summarized in the table.

Remember that in structural biology, the Root Mean Square Deviation (RMSD) is a quantitative measure of the distance between atoms of superimposed protein structures. RMSD values are presented in Å. Given two sets of n matched points $v = v_1, v_2, \cdots, v_n$ and $w = w_1, w_2, \cdots, w_n$ where $v_i = \left(v_{ix}, v_{iy}, v_{iz}\right)$ and $w_i = \left(w_{ix}, w_{iy}, w_{iz}\right)$ the $RMSD(v, w) = \left(\frac{1}{n}\sum_{i=1}^{n}\left((v_{ix} - w_{ix})^2 + \left(v_{iy} - w_{iy}\right)^2 + (v_{iz} - w_{iz})^2\right)\right)^{\frac{1}{2}}$

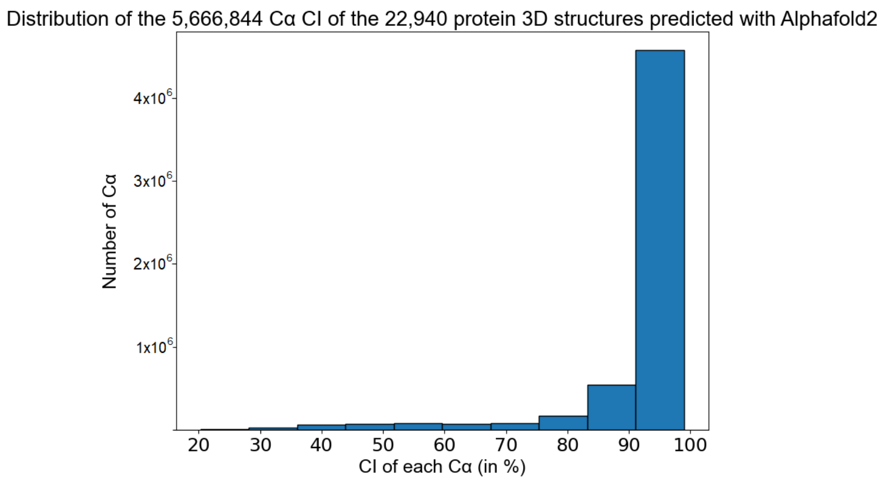

Figure S9. Histogram showing the distribution of 5,666,844 Cα confidence indices (CI) of the 22,940 protein structures predicted with Alphafold2. The x-axis corresponds to Cα CI, and the y-axis to the number of Cα. A percentage of 94% of Cα have CI ≥ 70%, and 83% of Cα have CI ≥ 90%.

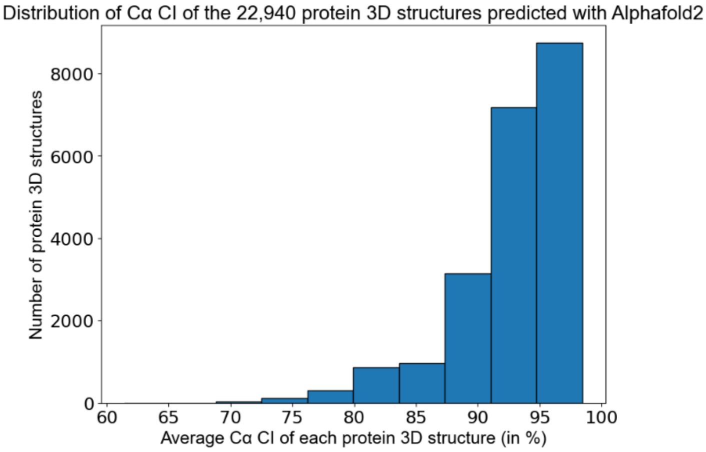

Figure S10. Histogram showing the distribution of average Cα confidence indices (CI) of protein structures predicted with Alphafold2. The x-axis corresponds to the average Cα CI of protein structure predictions, and the y-axis to the number of structures. A percentage of 99% of the structures predicted by Alphafold2 have an average Cα CI ≥ 70%, and 80% have an average Cα CI ≥ 90%.

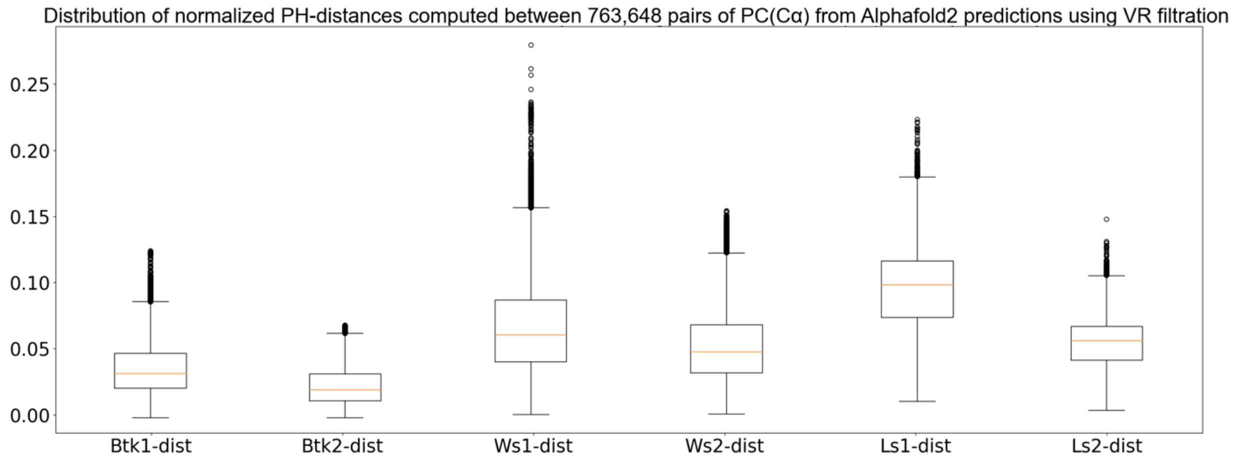

Boxplot	Lower whisker	Lower quartile	Median	Upper quartile	Upper whisker
Btk-dist / k=1	0.000	0.023	0.037	0.057	0.109
Btk-dist / k=2	0.000	0.011	0.022	0.037	0.077
Ws-dist / k=1	0.001	0.038	0.060	0.093	0.174
Ws-dist / k=2	0.000	0.025	0.035	0.057	0.125
Ls-dist / k=1	0.000	0.033	0.061	0.011	0.200
Ls-dist / k=2	0.006	0.002	0.037	0.059	0.127

Figure S11. Boxplots showing the distribution of normalized PH-distances calculated between pairs of homologous proteins using PC(Cα) from Alphafold2 structure predictions and the VR filtration. Btk1-distances, Ws1-distances, and Ls1-distances correspond to the homological dimension 1 (k=1), while Btk2-distances, Ws2-distances, and Ls2-distances correspond to the homological dimension 2 (k=2). The x-axis corresponds to the PH-distance types, the y-axis to normalized PH-distance values for the 763,648 pairs of homologous proteins. All p-values between pairs of boxplots are significant (< $5x10^{-3}$). Metrics for each distribution are summarized in the table.

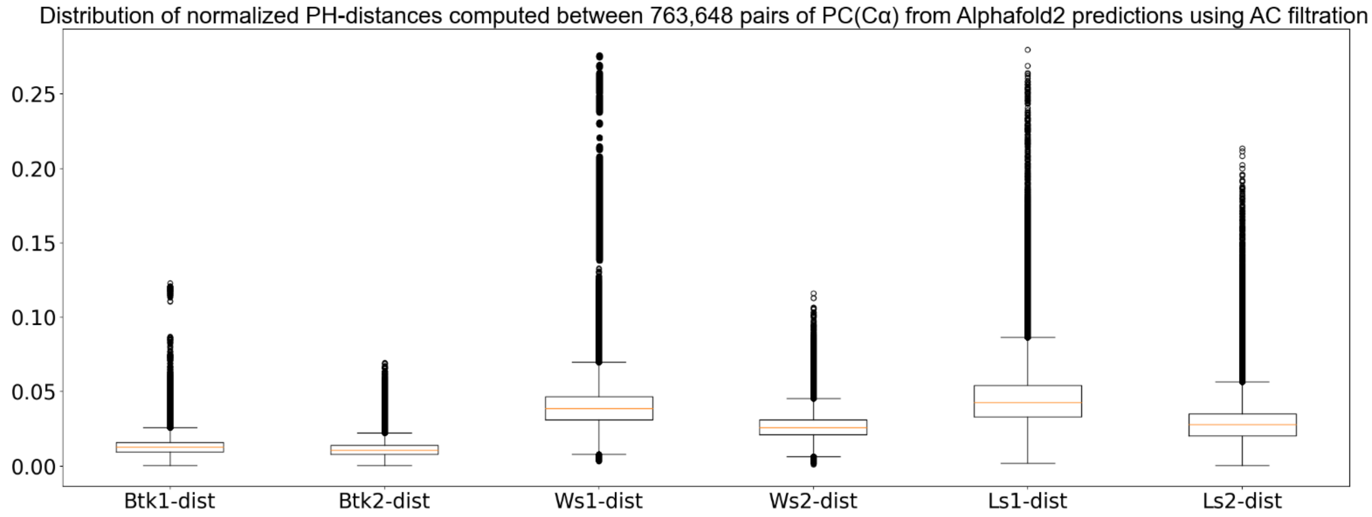

Boxplot	Lower whisker	Lower quartile	Median	Upper quartile	Upper whisker
Btk1-dist	0.000	0.009	0.013	0.016	0.026
Btk2-dist	0.000	0.008	0.011	0.014	0.022
Ws1-dist	0.008	0.031	0.038	0.047	0.070
Ws2-dist	0.006	0.021	0.026	0.031	0.046
Ls1-dist	0.002	0.033	0.043	0.054	0.086
Ls2-dist	0.000	0.020	0.028	0.035	0.057

Figure S12. Boxplots showing the distribution of normalized PH-distances calculated between pairs of homologous proteins using PC(Cα) from Alphafold2 structure predictions and the AC filtration. Btk1, Ws1, and Ls1-distances correspond to the homological dimension 1 (k=1), while Btk2, Ws2, and Ls2-distances correspond to the homological dimension 2 (k=2). The x-axis corresponds to the PH-distance types, the y-axis to normalized PH-distance values for the 763,648 pairs of homologous proteins. All p-values between pairs of boxplots are significant (2×10^{-3}). Metrics for each distribution are summarized in the table.

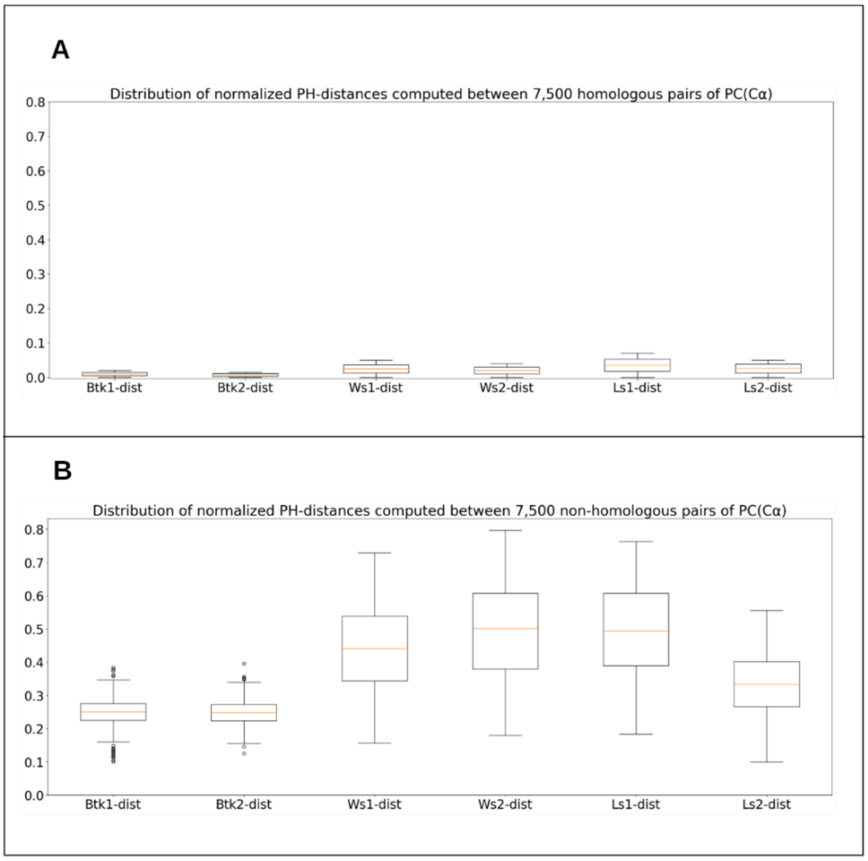

Figure S13. Boxplots showing the distribution of normalized PH-distances calculated between pairs of homologous proteins and pairs of non-homologous proteins using PC(Cα) from Alphafold2 structure predictions and the AC filtration. Btk1, Ws1, and Ls1-distances correspond to the homological dimension 1 (k=1), while Btk2, Ws2, and Ls2-distances correspond to the homological dimension 2 (k=2). The x-axis corresponds to the PH-distance types, the y-axis to normalized PH-distances. **(A)** PH-distances calculated for 7,500 pairs of homologous proteins randomly selected from the 763,648 pairs of homologous proteins and **(B)** 7,500 pairs non-homologous proteins randomly selected. As expected boxplots corresponding to the PH distances calculated from the 7,500 pairs of homologous proteins are very close to those calculated from all 763,648 pairs of homologous proteins (compare Figures S12 and S13A). All p-values between pairs of boxplots in (A) and (B) are significant ($<1x10^{-2}$). This means that the distances calculated between pairs of structures corresponding to homologous proteins are significantly different from those calculated for non-homologous proteins. The metrics for each distribution are summarized in the tables.

Boxplot (A)	Lower whisker	Lower quartile	Median	Upper quartile	Upper whisker
Btk1-dist	0.000	0.005	0.011	0.015	0.020
Btk2-dist	0.000	0.003	0.007	0.011	0.015
Ws1-dist	0.008	0.013	0.025	0.036	0.050
Ws2-dist	0.006	0.010	0.020	0.030	0.040
Ls1-dist	0.002	0.018	0.036	0.053	0.070
Ls2-dist	0.000	0.012	0.026	0.039	0.050

Boxplot (B)	Lower whisker	Lower quartile	Median	Upper quartile	Upper whisker
Btk1-dist	0.160	0.225	0.251	0.275	0.347
Btk2-dist	0.156	0.223	0.248	0.272	0.340
Ws1-dist	0.157	0.344	0.441	0.538	0.728
Ws2-dist	0.180	0.379	0.501	0.608	0.797
Ls1-dist	0.185	0.389	0.494	0.607	0.763
Ls2-dist	0.100	0.266	0.333	0.402	0.555

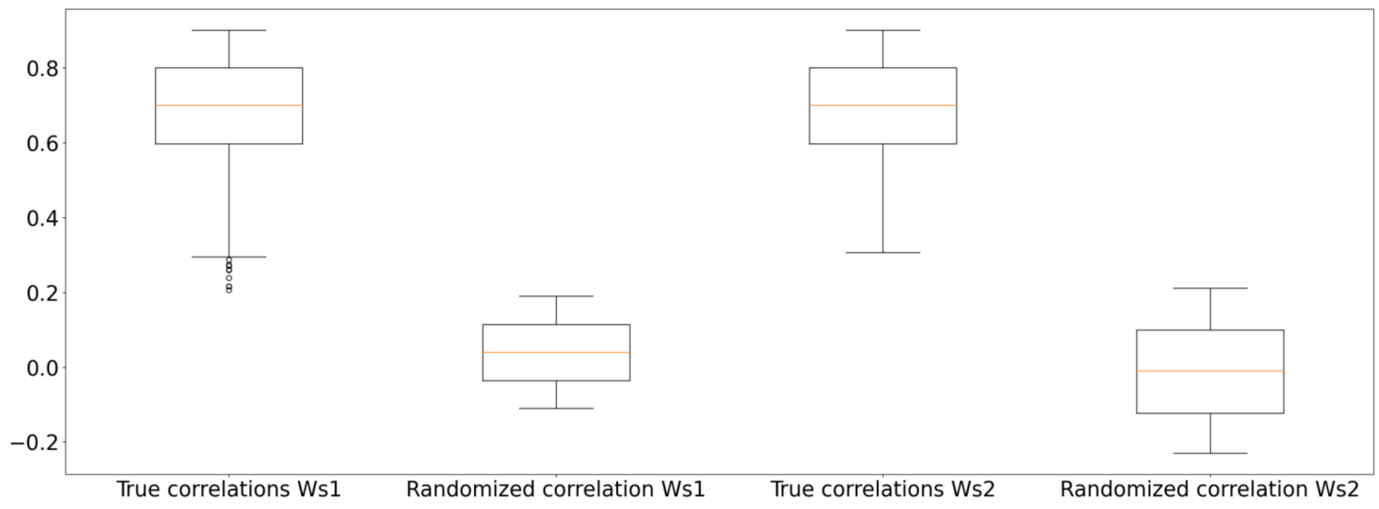

Figure S14. Boxplots showing the distribution of correlation coefficients on the 518 protein families between ML- and Ws-distance in homological dimensions 1 (Ws1) and 2 (Ws2).

To go further, we determined whether the observed correlations between the ML distances and the PH distances could be obtained by chance. Thus, for each of the 518 families, we calculated the correlation coefficient when the pairs of sequences compared correspond to the pairs of structures compared (e.g. comparing the ML distance calculated between the sequences of proteins A and B with the PH distance calculated between the structures of proteins A and B). More precisely, for each protein family, to calculate this “true” correlation coefficient, we compare the ML distances and PH distances of 1,000 randomly selected A-B proteins pairs. We also calculated the correlation coefficient (randomized correlation) when the pairs of sequences compared did not correspond to the pairs of structures compared (e.g. comparing the ML distance calculated between the sequences of proteins A and B with the PH distance calculated between the structures of proteins C and D). More precisely, for each protein family, to calculate this “randomized“ correlation coefficient, the ML distances of 1,000 randomly selected pairs of A-B proteins were compared with the PH distances of 1,000 randomly selected pairs of C-D structures. All p-values between pairs of boxplots are significant ($<4x10^{-4}$). This means that the Ws1 distributions are significantly different and that the Ws2 distributions are also significantly different. Strong correlations are observed when ML and PH distances are obtained from the same proteins, whereas they are not significant in the other case. This again demonstrates that PHs allow a clear phylogenetic signal to be captured in structures. The metrics for each distribution are summarized in the table.

Boxplot	Lower whisker	Lower quartile	Median	Upper quartile	Upper whisker
True correlations Ws1	0.30	0.60	0.70	0.80	0.90
Randomized correlation Ws1	-0.11	-0.04	0.04	0.11	0.19
True correlations Ws2	0.31	0.60	0.70	0.80	0.90
Randomized correlation Ws2	-0.23	-0.12	-0.01	0.10	0.21

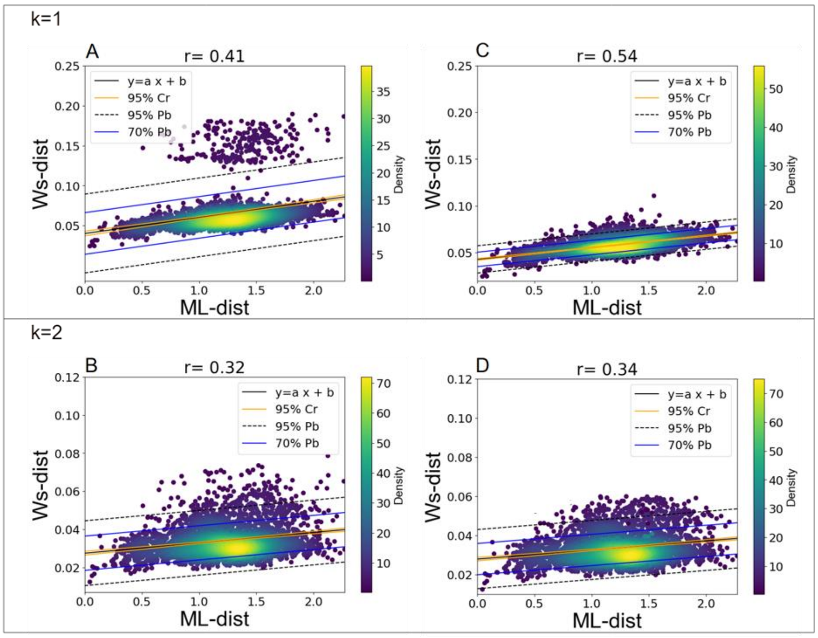

Figure S15. Correlation plots between ML-distances and normalized Ws-distances in homological dimensions 1 and 2 for the P9WNX1 protein family. This *Corynebacteriales* protein family contains 80 homologous protein sequences. Panels **(A)** and **(B)** = correlation plots between ML- and normalized Ws-distances in homological dimensions 1 and 2 when amino acid sites corresponding to indels are considered. Panels **(C)** and **(D)** = correlation plots between ML-distances and normalized Ws-distances in homological dimensions 1 and 2 when amino acid sites corresponding to indels are omitted. Each plot contains 3,160 points, each of them corresponding to a pair of homologous proteins. For each pair of proteins, the ML-distance computed from the ML-tree (x-axis) is compared with the Ws-distance computed between the structures predicted by Alphafold2 using the AC filtration on PC(Cα) (y-axis). For each panel, the orange line is the 95% confidence regions in which the true regression line should belong. The solid light blue lines are the 70% interval within which future individual data points or observations should fall. The dashed dark blue lines are the 95% interval within which a future individual data point or observation should fall. The black line corresponds to the regression line (y=ax+b). Point colors correspond to the density values according to the density scale. The wide dispersion of the points around the regression line observed in both homological dimensions is due to the presence of outliers, i.e. protein pairs with abnormal PH-distances compared to ML-distances. Close examination of the multiple alignment of the P9WNX1 protein family revealed that these outliers correspond to protein pairs involving the sequences of *Corynebacterium vitaeruminis* and *Hoyosella rhizosphaerae*, which contain a large deletion between positions 1 to 17 of the multiple alignment (Figure S16).

```
                                  10        20        30        40        50        60        70        80        90        100       110       120       130
                         ....|....|....|....|....|....|....|....|....|....|....|....|....|....|....|....|....|....|....|....|....|....|....|....|....|....|....|.
Tsukamurella_paurometabola|DSM  IGLIWAQASGVIGDGGIPWHIPEDMKFFRETTAGATVVMGRKTWDSLPARFRPLPGRTNIVVTADGAVVHELVLP--WVIGGGEIYAAALPSADLLAVTEVDADIP-GDTYAPSIPDTGWRESTLRYRHLTYRRT
Segniliparus_rotundus|DSM_4498  VGVLWAQGNGVIGIDGELPWSIPEDMRRFRQLTVGHTVVMGRKTWDSLPARFRPLPERTNVVLSAQGAQVADVVTATAWVIGGGEVYAKAVHLAAIAEITEVDFEVE-GDVYAPQLGEGPWQRSELRYRFTTFRRA
Segniliparus_rugosus|ATCC_BAA-  VGVVWAQGNGVIGIDGELPWSIPEDMRRFRQLTVGHTVVMGRRTWDSLPEQFRPLPKRTNVVLSAEGAQVEDIVSATAWVIGGGEVYAKAVHLASVAEITEVDFEVE-GDAYAPQLAEGPWQYSELRYRFTTFQRA
Gordonia_araii|NBRC_100433@GCF  VQLVWAQADGAIGRDNAIPWRVPEDMARFKELTIGYPVIMGRKTWDSLPPKFRPLPHRRNIVLTAEGAVRATLDEALVSVIGGAEVYRVAMDRATELRVTEIDVEVPDADAFAPPIGTGDWLLSKERYRFVNYRPV
Nocardia_higoensis|NBRC_100133  IGLIWAQSEGVVGFENTIPWRVPEDMANFRTVTWGHPVIMGRKTWDSLPPKFRPLAGRRNIVLTAPGAERASLTEALAWVAGGAEIYATAMDFATELLVTEVQVSVQ-GDAHAPTIGSEPERVSSLRFRIDRYVRR
Corynebacterium_durum|F0235@GC  LRAIWAQTDRVIGDGYDMPWYLPEDLAHFKNTTLDHPIIMGRATWESLPR--RPLPGRDNIILSSTGATVTEPP----WIIGGGQVYASTLPLVDAITMTLIDAHLANRAVFAPDITETDWLTSELRYKYITYVRT
Gordonia_aichiensis|NBRC_10822  ITLIWAQDVGAIGRQNAIPWRVPEDMRRFRELTGSDPVVMGRKTWESLPERFRPLPARRNLVISATGAEVVSLRDALDTVMGGGAIYEAAMEHASRLQVTEIDLLVEGADAFAPEIDTTSWQTSSLRYRFVEYSRH
Gordonia_sihwensis|NBRC_108236  ITLVWAQDAGAIGRAGVIPWRVPEDMRRFKEITGGSTVIMGRKTWESLPTAVRPLPGRRNIVITADGAEIVGLEAALDAVIGGGEIYAAAIDRATHLRVTEIDVLVDGADAFAPVVDVGEWLYSSTLYRFIDYRRR
Gordonia_soli|NBRC_108243@GCF_  IELVWAQDNRAIGKGNTIPWRVPEDMRHFREVTGDSPVIMGRRTWDSLPVRFRPLPGRHNIVVSLDGADVVSVEAALDAVIGGGQIYTAALPFATAVRLTEIDIAVDAPDAFAPVIDAGEWLTSRPRYRFVDHRRA
Corynebacterium_capitovis|DSM_  KCAIWAQSDGVIGDGTSLPWHLPEDLKHFKTITLGSPIVMGRRTWESLTL--RPLPGRENIIISSAGAHVAEFPPL--WIIGGAQLFSATLPLVDRIERTLIDVHLAARAVILPDVPQSRWETSALRYSFQRFERI
Jongsikchunia_kroppenstedtii|N  LALIWAQAGGVIGRDGDIPWHIPEDMKYFRETTSGHPVIMGRRTWDSLPPRFRPLPGRRNIVVTAEGATAVSVTDACAYVIGGGEIYSAAMEQADRLHVTEIDEVID-GDTRAPAIGDEPWTVTAQRVRWLTYRR-
Smaragdicoccus_niigatensis|DSM  IGMIWAQAGRVIGSENSIPWHVPEDFAHFRMVTMGQTVVMGRRTWESLPK--KPLPGRRNVVVTPNGAERASLEEALTWVIGGAEIYRAAMPLADVLEITEVDIYVP-GDVLAPEIPAGEWQVSVPKYRFLRYERR
Tomitella_biformata|AHU_1821@G  LGMIWAWSGGVIGLDDAMPWHLPEDMAHFRATTSGHPVIMGRVTWDSLAPRFRPLPSRRNIVVTAEGAEVATPSAAMEWVMGGAQIYAALLPLADHLEVTEIDADFD-GDTRLPPLGPGPWLFSELRYRFVSYRRL
Corynebacterium_vitaeruminis|D  -----------------MPWHLPEDLAHFKETTLGQPVLMGRATWESIPERFRPLPGRDNVVLSSRGALVVKLPAT--WVIGGGSVYAATLPQVEEVVVTLIDATLADKAVKAPPLTETEWRTSRLRYRFQWYRLR
Williamsia_herbipolensis|ARP1@  VALIWAQGDRAIGRDNTIPWRVPEDARHFRELTRGHRVVMGRRTWDSLPERFRPLPDRVNVVLTSPGAVVADLPEALAMVMGGSQIYDAAMTFADVLHVTEIDVDVDGADAHAPEIDHGEWQESTLRYRFLDYRAR
Mycobacterium_lepromatosis|FJ9  VSLVWAQSTGVIGRGGGIPWRVPEDLTRFKQLTMGHPVVMGRRTWDSLPAGVRPLSGRRNVVLSAEGAEVFSLEETISWVIGGGQIYRLALPLATRCGVTEVDTDLPDDDVLAPVLDTGEWLVSCLRYRLHSYRRS
Corynebacterium_mustelae|DSM_4  LSAIWAQNTGIIGDGTAMPWHVPEDLKHFKEKTLGSDVIMGRKTWESLPK--TPLPGRKNIIISSAGAEVTTIP----WIIGGGQLYAATIADVAVIERTLIDAPLTGAAVYAPDIPRSEWLLSTLYYRFETWVRG
Mycobacterium_heraklionense|Da  ISLIWAQSSGVIGRAGGIPWRVPEDQARYKQLTVGHTVVMGRLTWESLPAKVRPLPDRRNVVLTADGATVVTLEQALDWVVGGAEIYALALPLASRCEVTEVDIDLPDGDTVAPVLDTGEWLTSRLRYRYLTYHRT
Lawsonella_clevelandensis|X103  IGLIWAQSDGWIGRDGTMPWHVPEDLAHFKHITNADPVVMGRVTWDSLNPRFRPLPGRPNIVLTAPGAQVVSPLEALEWVIGGSHIYREFMPIAHRCEVTELDITVAGADAAAPRLDTTQWERSTVRYRFLTFDRI
Millisia_brevis|NBRC_105863@GC  VGLIWAQAGGVIGANGGIPWRVPEDMKRFREITTGGRVVMGRRTWDSLPKRFRPLPDRENVVISLDGARVVDLDAALTWVIGGGQIYAAAIDRADILEVTEVDLDVA-GDTYAPPIGPSDWRESTTGFRFVSYRRI
Dietzia_cinnamea|NBRC_102147@G  --MVWAQAGGVIGDGRDMPWHIPEDLAFFKQATVGRPVVMGRATWDSIPERFRPLPGRRNIVCTADGAERASVDAALAVVMGGGQVYAAALPAADECLVTEIDADAP-GTVLAPTLDVGEWITDPVRHRHTVWRRR
Nocardia_uniformis|NBRC_13702@  IGLIWAQTDGVIGLDNAIPWRIPEDMAHFKDVTRGHPVVMGRRTWDSLPARFRPLDGRRNIVVTADGADRASLDEALRWVMGGGEIYRAALPLATELMVTEVDSDIV-GDCYAPTIGEAPWRESTLRYRFRRYTR-
Rhodococcus_fascians|D188@GCF_  VGLIWAQSNLKIGEAGRIPWEVPEDMARFKAVTMNHPVIMGRKTWESLPERFRPLPGRRNIVVTAPGAETCGVDEALTWIIGGGEIYRSAMPFATTLHVTEIDVEVP-GDTDAPTVETGDWLTSRRQFRYVEYGKP
Mycobacteroides_chelonae|CCUG_  IGLIWAQSDGVIGADGAIPWRLPEDQARFKRITMGHTVIMGRKTWESLPSSVRPLPGRPNIVLTPDGALAVCADAALAWVIGGGEIYRLFLPLAQRCEVTVVDADVP-GDALAPELDGNDWQTSELRYQFLSYRKV
Aldersonia_kunmingensis|DSM_45  VRLVWAQTTGVIGSGNDIPWHLPEDLAHFKRVTMGSAVVMGRKTWDSLPERNRPLPGRRNIVVTGYGAEPASVRAAIAAVIGGAQIYDAALPAATELVVTEVDLDVS-GDAFAPAIGDGEWQRSKTAFRIRRYVRS
Hoyosella_altamirensis|NBRC_10  MRMIWAQSGRVIGNQGAIPWHIPEDMAHFRETTQGCPVIMGRATWDSLKPKFRPLPGRDNIVVTAEGAIVASLPEAFEWVMGGSQIYAEALPYASLAVVTEVDIDVP-GDAFAPELDAEGWHTSKHRYRYLRYLRP
Dietzia_timorensis|ID05-A0528@  LRMVWAQADRVIGTGDGMPWHVPEDMKHFVQVTRGNTVLMGRATWESLTPKFRPLPGRRNIVLSPEGAEVATLDEAVDDVLGGGSIYAALHDMASEAIVTEFDADAI-GAVRAPELSTSEWETSEVRYRFIRYERI
Rhodococcus_maanshanensis|NBRC  LGLIWAQSAGVIGRDGAIPWRIPEDMAYFKDVTSGHPVVMGRKTWDSLPPRFRPLTGRRNIVVTAEGAEAASVESAITWVMGGGEIYSAAMPFADRLVVTEVDVDTE-GDAYAPTIGTGDWLTSELKFRFRTYIRG
Rhodococcus_marinonascens|NBRC  VILVWAQAGGVIGRGNSIPWHVPEDMAFFKKVTYGSPVIMGRKTWDSLPPRFRPLPGRRNIVISAEGADVACIEAALSCVIGGAQIYTAAMPFATDLVVTEIGLTID-GDAWAPPIDTGEWCISATRFRRVSYTRA
Corynebacterium_sphenisci|DSM_  LGMIWAQSDGVIGDGTAMPWHLPEDLAHFKATTLGAPVIMGRRTWDSLNPRFRPLPGRRNIVLSFPGAEHADLDAALAWIIGGGRVYREGLALADECVVTEVDAHPADTPVTAPDLRRGDWRTAAPRYRVTRWRPR
Mycobacterium_bohemicum|DSM_44  VGLVWAQSSGVIGRGGDIPWRVPEDLARFKEITMGHTVVMGRRTWESLPARVRPLPGRRNVVLSAEGAEVVRLDDALSWIIGGAQIYLLGLPLATRCEVTEVDIALPDDDALAPVLDAGDWQVSRLRYRFHTYRRA
Mycobacterium_marseillense|FLA  VGLVWAQSSGVIGRGGGIPWNVPEDLARFKEVTMGHTVVMGRRTWESLPTKVRPLPGRRNVVLSAEGAQVVSLDAALAWVIGGGQVYLAALPYATRCEVTEIELDLRDDDALAPALDTGEWQASRLRYRFHSYRR-
Corynebacterium_glaucum|DSM_30  LGSIWAQSDGVIGDGVGMPWHLPEDLKHFKDTTMGHPIIMGRRTWEALPI--QPLPGRVNHVLSSDGAYVSDLPDL--WIIGGGQLYEATLDEVDVIERTLIDATLE-TPVYAPRIGDGEWQTSTLRYKFQRFERK
Dietzia_natronolimnaea|S-XJ-1@  --MVWAQAGGVIGDGRDMPWHIPEDLAFFKQATVGRPVVMGRATWDSIPERFRPLPGRRNVVCTADGAERASVERALEAVMGGGQIYAAALSAADECLVTEIDAEAP-GVVLAPELDVGEWITDPVRHRHTVWRRR
Mycolicibacterium_brumae|CIP10  LSLIWAQASGVIGRDGDIPWRLPEDLAHFKELTVGHTVVMGRRTWESLPASVRPLPGRRNVVLTADGAEVLSLADALDWVMGGAEIYRLALPLATRCEVTEVDLDLPAGDALAPRLDAGDWRCSALRHRFLSYRR-
Mycobacterium_palauense|CECT_8  LSLIWAQSSGVIGRANGIPWRLPEDQARFKQLTMGHTVVMGRKTWESLPPKVRPLPGRRNVVVTAEGATVVSLDEVPLWVIGGAQIYGEALPLASRCEVTEIAVDLDDEDAMAPVLDAGEWLTSTLRYRFLHYRR-
Corynebacterium_diphtheriae|IS  LRAIWAQSDGVIGDGNDMPWHVPEDLAYFKATTLGAPVIMGRTTWTTIPERFRPLPGRRNFVLSSTGAEVVAIPEL--WIMGGGAVYTSTLPLVDEVVITLIDATLADAAVYAPALDESPWQQSAARYKFQRLRRR
Gordonia_iterans|Co17@GCF_0029  IVLIWAQDAGAIGRRGTIPWTVPEDLAHFREVTGDGAVIMGRKTWDSLPDRFRPLPGRRNIVLTADGAETADLDAALDAVIGGGEIYRAALRHASRLHVTEIGMLVTGADAFAPEIDATDWQHSRTLYRFLEYRRI
Williamsia_marianensis|DSM_449  IALIWAQADGAIGRGGDIPWHLPEDQRYFRQITGGHPVVMGRRTWDSLPARFRPLPGRANLVVTADGAQTHNVRDALDFVMGGGEIYSAAMESAHEAYVTEIDLEVSDADTWAPALDDGGWLESPLRYRFLRYSAS
Williamsia_limnetica|DSM_45521  IALIWAQAGGVIGRDGDMPWHLPEDQKYFRATTGHHPVVMGRRTWDSLPERFRPLPNRANLVITGVGAQRFDVASALAFVMGGGEIYRGAIDLAHVAYVTEIDIDVADGDTIAPELDDEDWLTSALRYRFLRYSAQ
Mycobacterium_leprae|MRHRU-235  VGLIWAQSTGVIGRDGGMPWRVPEDLTRFKQLTMGHPVVMGRRTWDSLPVGVRPLPGRRNIVLSAEGAEVFSLEETISWVIGGEQIYRLALPLATRCGVTEVDTYLLDDDVLAPVLDTGEWLVSCLRYRLHSYHRS
Nocardia_pseudobrasiliensis|DS  IGLIWAQTRGVIGVDNAIPWRVPEDMAHLKEITRGHPVVMGRRTWDSLPPRFRPLPDRRNIVVTAAGAERASVKEALRWVLGGGEIYRAAMDYATDLEVTEIDADID-GDTYAPEIGDSGWRESSLRYRFLHYLR-
Rhodococcus_spongiicola|LHW505  IGLIWGQTAGVIGKDNAIPWRIPEDMSHFKETTLGHPVVMGRLTWDSLPSRFRPLPGRRNIVITAEGALTAGIEEALTWVMGGSQIYTAAMPFADLLSVTEIDADID-GDAFAPKIGEGDWRISDLRYRFLTYVRA
Mycobacteroides_abscessus|GZ00  IGLIWAQTAGVIGADGAIPWRLPEDQARFKRITMGHTVIMGRKTWESLPGSVRPLPGRPNIVLTPDGALAVCADAALAWVIGGGEIYRLFLPLAQRCEVTVVEADVP-GDALAPELGENDWQTSELRYQFLSYRKV
Tsukamurella_conjunctivitidis|  IGLIWAQAGGVIGADGGIPWHIPEDMKFFRETTAGATVLMGRRTWDSLPPRFRPLPGRTNIVVTADGAVVQDLTLP--WVMGGGEIYAAALPHADLLVVTEVDADID-GDTRAPSIPDAAWRESTLRYRHLTYRRN
Mycobacterium_grossiae|DSM_104  LGLIWAQSSGIIGRDGGIPWRLPEDQARFKDLTMGHTVVMGRLTWESLPAKVRPLPGRRNVVLTADGATVVGLEQALTWVIGGAQIYALALPESATRCEVTEIDVDLPDDDAVAPVLDDGEWLTSALRYRFSTYVRS
Nocardia_aurantia|RB56@GCF_009  VGLIWAQTDGVIGAGNTIPWRVPEDSAHFKNLTWGHPVIMGRRTWDSLPDRFRPLPGRRNIVITADGAERASLESALAWVAGGGEIYRAALGRATELHVTEVDADIA-GDAYAPAIGDGPWTTSRLRYRFRRYTR-
Gordonia_crocea|NBRC_107697@GC  VQLVWAQGDGAIGRDNTIPWRVPEDMARFKELTIGYPVIMGRKTWDSLPPKFRPLPDRRNIVLTAEGAVRATLDEALVSVIGGAEVYRAAMDRATELRVTEIDVEVPGADAFAPPIGTGDWLVSRDRYRFVNYRPA
Mycobacterium_bourgelatii|JCM_  VGLVWAQSSGVIGRGGDIPWRLPEDLNHFKQITMGHTVVMGRRTWESLPARFRPLPGRRNVVLSAPGAEVVSLDEALTWVVGGSEIYTLALPYASRCEVTEVDIDLPDEDAIAPALDTGDWLVSKLRYRFHSYRRS
Mycolicibacterium_mageritense|  MALIWAQSSGVIGRDNTIPWRLPEDQARFKELTLGQTVVMGRLTWESLPAKVRPLPGRRNVVVTADGAEVVELGDVFRWVIGGAQIYRQALPFADRCEVTEIDVDLPAGDAMAPVLDSGEWLTSSLRYRFCTYTRQ
Mycolicibacterium_gadium|JCM_1  VGLIWAQSSGVIGRRGGIPWRLPEDQARFKELTMGRTVVMGRLTWESLPAKVRPLPGRRNVVLTADGALVVLLQDALTWVIGGEQIYMLALPLATRCEVTEVEIDLPDGDAVAPTLDEGEWLTSSLRYRFFSYVRP
Mycolicibacterium_phocaicum|JC  MKLIWAQGGGVIGRDNDIPWQLPEDMAHFKEQTLGQTVVMGRLTWESLPPRFRPLPGRRNIVVSAEGAEVVSLPAEIDWVMGGAQLYALALPVATLCEVTEIDVDVPGGDAFAPALDHSEWLTSRLRYRFCTYQR-
Mycolicibacterium_monacense|JC  VGLIWAQSSGVIGRDGGIPWHLPEDLARFKEITLGHPVVMGRLTWESLPPKVRPLPGRKNVVLTAEGAEVVSLDDALGWVIGGERIYTLALGRATRCEVTEVEIELHDADAVAPVLGAGDWLTSVLRYRFHSYQRA
Mycobacterium_koreense|JCM_199  TGLIWAQSTAVIGRDGTIPWHLPEDLAHFKALTTGRTVVMGRRTWESLPEAVRPLPKRRNIVVTAACAQVVDLESVIAWVIGGGEIYALALPRATRCEVTEVDAAVTPGDVLAPVLDVGDWQTSRLRYRFVSYRRA
Mycolicibacterium_anyangense|J  VGLIWAQSSGVIGRDGGIPWRLPEDLARFKGLTMGHTVLMGRRTWESLPAAVRPLPGRKNVVLTADGATVVDLEQALEWVIGGSEIYHLALPMTTRCEVTEVEVDLRDEDALAPMLDSGDWQTSSLRYRFHHYLRA
Fodinicola_acaciae|GKU_173@GCF  IGVEWAQSNGVIGRDGVLPWHLPEDMAHFRTLTAGATVLMGRRTWESLPPRFRPLPGRRNLVLS-DGVETFDLSQALAWVMGGAAVYEAALPYADRIVVTEIQESFD-GDTYAPKIGVGDWQESSLHYRFLTWG--
Corynebacterium_qintianiae|MC1  IGAIWAQSDGVIGDGAGMPWHLPEDLKHFKDITLGHPVIMGRRTWEALPK--RPLPGRPNHILSSDGAFVSDLPDI--WIIGGAQIFEATLDEVDVIELTLIDASYAPEAVIAPRIPQSEWLTSTLRYQFQRLERN
Antrihabitans_stalactiti|YC2-7  VSLIWAQTAGVIGRDGAIPWQLPEDIAHFKAVTMGHPVVMGRKTWDSLPQRFRPLTGRRNIVVTADGAETVSLADALAWVIGGGELYTAAISFAHTLSVTEIDADID-GDAFAPAIGDAPWLTSTLRYRFVKYVRA
Gordonia_asplenii|TBRC_11910@G  VRLVWAQDNGGIGKAARIPWRVPEDMARFKELTIGHPVVMGRKTWESLPPKFRPLPGRRNIVVTADGAEVASVDDAVRSVIGGGEIYRAAMDLATELLVTEVDTAVEGADAHAPRIGVGPWRTSTLGYRFVDYRRR
Tomitella_gaofuii|HY172@GCF_01  LGMIWAQAGGVIGAGGAMPWHLPEDLAHFKAVTAEHPVIMGRATWDSLPPRFRPLPGRRNIVVTADGATAVSTMEALEWVTGGAQIYSALLPHADLCAVTDIDVDVD-GDTLAPALDPAEWQVSALRYRFRTLRRH
Rhodococcus_trifolii|CCM_7905@  VGLIWAQSSGVIGADGGIPWRIPEDMAHFREVTAGGRVVMGRRTWDSLPARFRPLPGRTNLVMTAEGAQRVSLDEVLDWVIGGGEIYRAALPFADVLEVTEVDVDIE-GDTVAPEVPVGDWQTSSIRYRYVRYTSG
Hoyosella_rhizosphaerae|CGMCC_  -----------------MPWHIPEDLKHFKRVTDGSAVLMGRRTWDSLPERFRPLPGRDNIVITAPGAIVVSLEDAIAWVMGGAQIYEALMPHATQLVVTELDIAVP-GDAYAPAIDATGWHTSRMPFRWVWYVRS
```

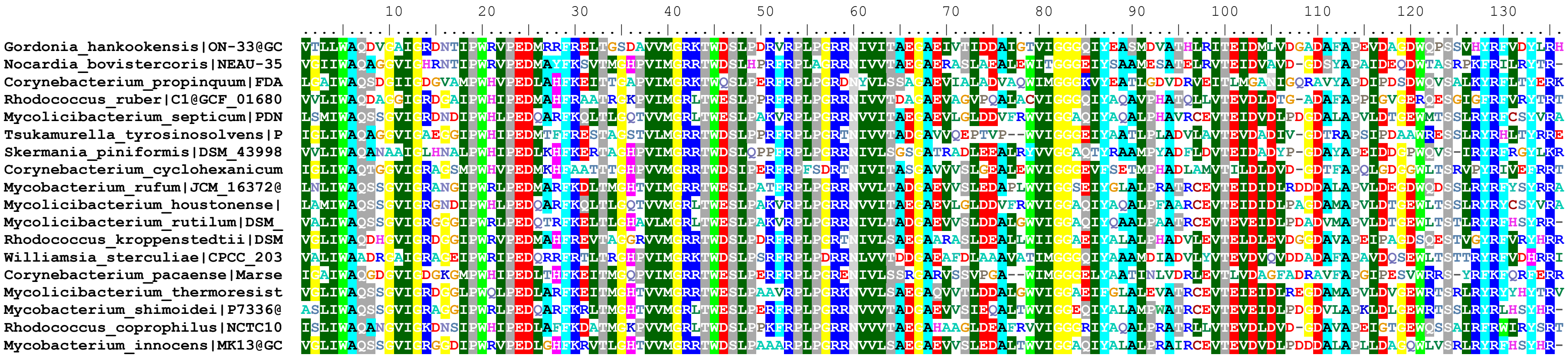

Figure S16. Trimmed multiple alignment of the P9WNX1 protein family. A deletion is present between position 1 and 17 (in black) in the sequences of *Corynebacterium vitaeruminis* and *Hoyosella rhizosphaerae*.

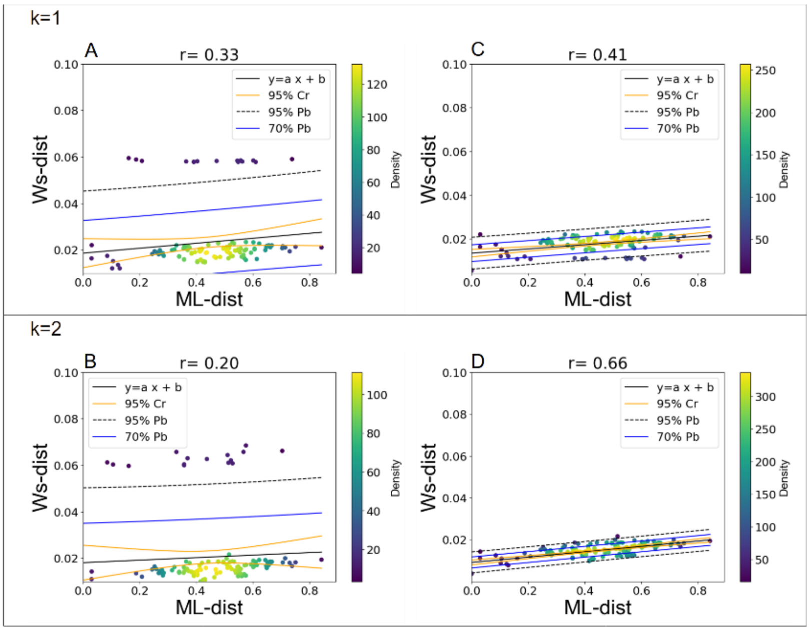

Figure S17. Correlation plots between ML-distances and normalized Ws-distances in homological dimensions 1 and 2 for the P58502 protein family. This *Thermococcales* protein family contains 17 homologous protein sequences. Panels **(A)** and **(B)** = correlation plots between ML- and normalized Ws-distances in homological dimensions 1 and 2 when amino acid sites corresponding to indels are considered. Panels **(C)** and **(D)** = correlation plots between ML-distances and normalized Ws-distances in homological dimensions 1 and 2 when amino acid sites corresponding to indels are omitted. Each plot contains 136 points, each of them corresponding to a pair of homologous proteins. For each pair of proteins, the ML-distance computed from the ML-tree (x-axis) is compared with the Ws-distance computed between the structures predicted by Alphafold2 using the AC filtration on PC(Cα) (y-axis). For each panel, the orange line is the 95% confidence regions in which the true regression line should belong. The solid light blue lines are the 70% interval within which future individual data points or observations should fall. The dashed dark blue lines are the 95% interval within which a future individual data point or observation should fall. The black line corresponds to the regression line (y=ax+b). Point colors correspond to the density values according to the density scale. The wide dispersion of the points around the regression line observed in both homological dimensions is due to the presence of outliers, i.e. protein pairs with abnormal PH-distances compared to ML-distances. Close examination of the multiple alignment of the P58502 protein family revealed that these outliers correspond to protein pairs involving the sequence of *Thermococcus cleftensis*, which contains a large deletion between positions 1 to 29 of the multiple alignment (Figure S18).

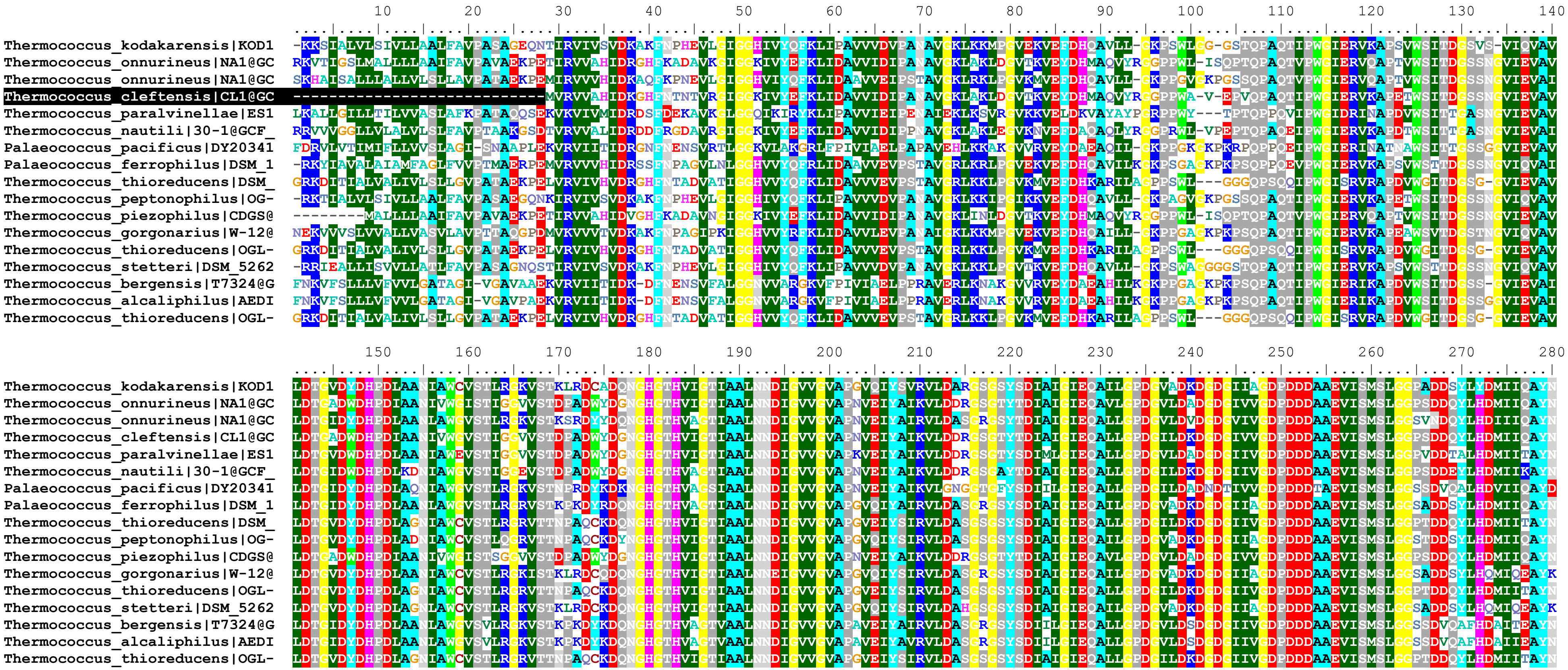
Thermococcus_kodakarensis|KOD1
Thermococcus_onnurineus|NA1@GC
Thermococcus_onnurineus|NA1@GC
Thermococcus_cleftensis|CL1@GC
Thermococcus_paralvinellae|ES1
Thermococcus_nautili|30-1@GCF_
Palaeococcus_pacificus|DY20341
Palaeococcus_ferrophilus|DSM_1
Thermococcus_thioreducens|DSM_
Thermococcus_peptonophilus|OG-
Thermococcus_piezophilus|CDGS@
Thermococcus_gorgonarius|W-12@
Thermococcus_thioreducens|OGL-
Thermococcus_stetteri|DSM_5262
Thermococcus_bergensis|T7324@G
Thermococcus_alcaliphilus|AEDI
Thermococcus_thioreducens|OGL-

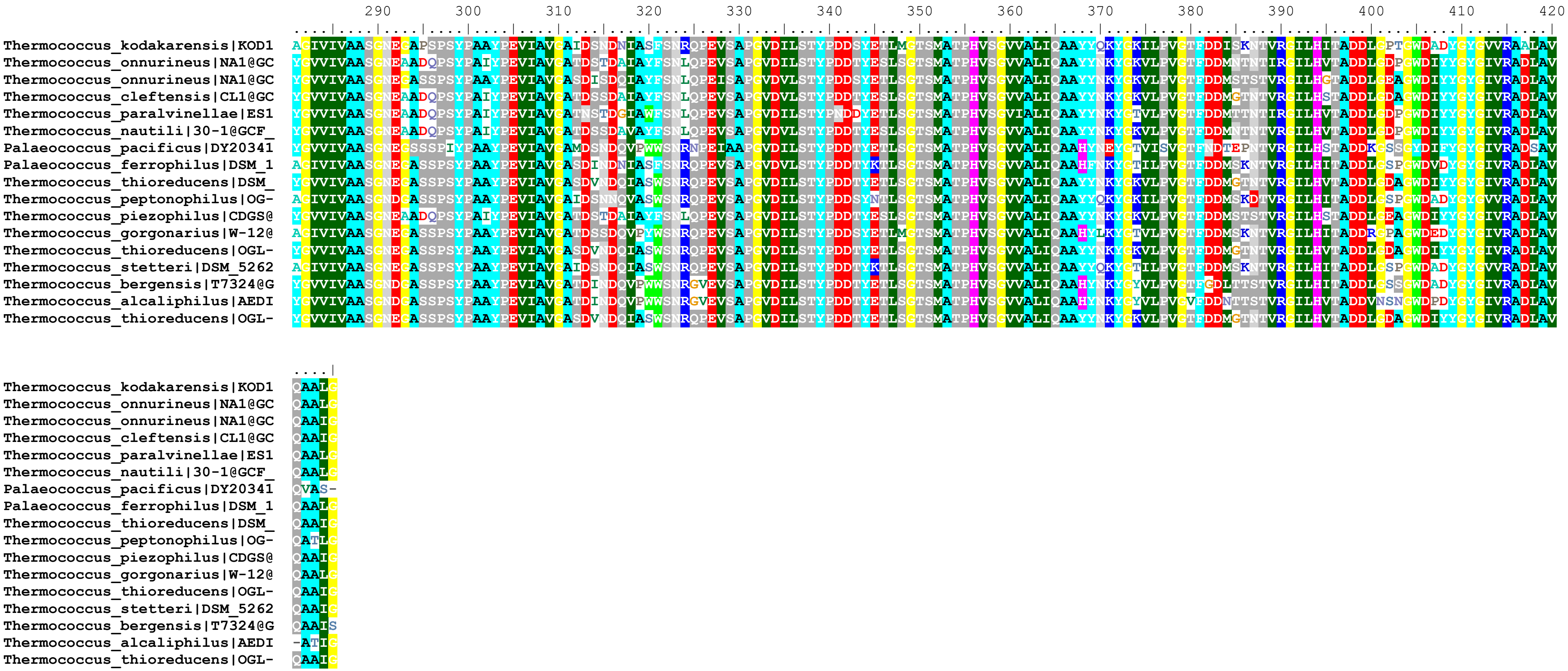

Figure S18. Trimmed multiple alignment of the P58502 protein family. A deletion is present between position 1 and 29 (in black) in the sequence of *Thermococcus cleftensis*.

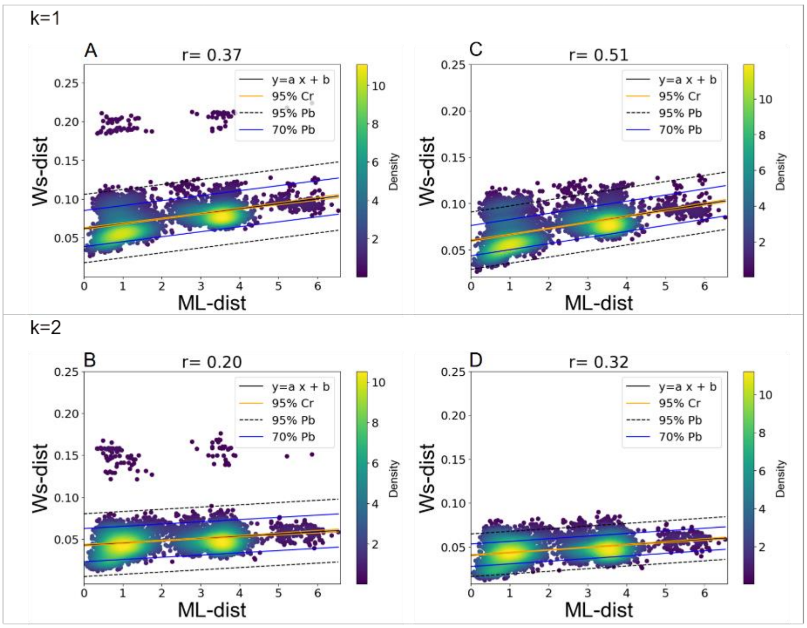

Figure S19. Correlation plots between ML-distances and normalized Ws-distances in homological dimensions 1 and 2 for the Q9HXX5 protein family. This *Pseudomonadales* protein family contains 98 homologous protein sequences. Panels **(A)** and **(B)** = correlation plots between ML- and normalized Ws-distances in homological dimensions 1 and 2 when amino acid sites corresponding to indels are considered. Panels **(C)** and **(D)** = correlation plots between ML-distances and normalized Ws-distances in homological dimensions 1 and 2 when amino acid sites corresponding to indels are omitted. Each plot contains 4,153 points, each of them corresponding to a pair of homologous proteins. For each pair of proteins, the ML-distance computed from the ML-tree (x-axis) is compared with the Ws-distance computed between the structures predicted by Alphafold2 using the AC filtration on PC(Cα) (y-axis). For each panel, the orange line is the 95% confidence regions in which the true regression line should belong. The solid light blue lines are the 70% interval within which future individual data points or observations should fall. The dashed dark blue lines are the 95% interval within which a future individual data point or observation should fall. The black line corresponds to the regression line (y=ax+b). Point colors correspond to the density values according to the density scale. The wide dispersion of the points around the regression line observed in both homological dimensions is due to the presence of outliers, i.e. protein pairs with abnormal PH-distances compared to ML-distances. Close examination of the multiple alignment of the Q9HXX5 protein family revealed that these outliers correspond to protein pairs involving the sequences of *Pseudomonas indica*, *Pseudomonas akapageensis*, *Pseudomonas guryensis*, *Pseudomonas panipatensis Pseudomonas benzenivorans*, and *Halopseudomonas sabulinigris*, which contain a deletion between positions 1 to 10 of the multiple alignment (Figure S20).

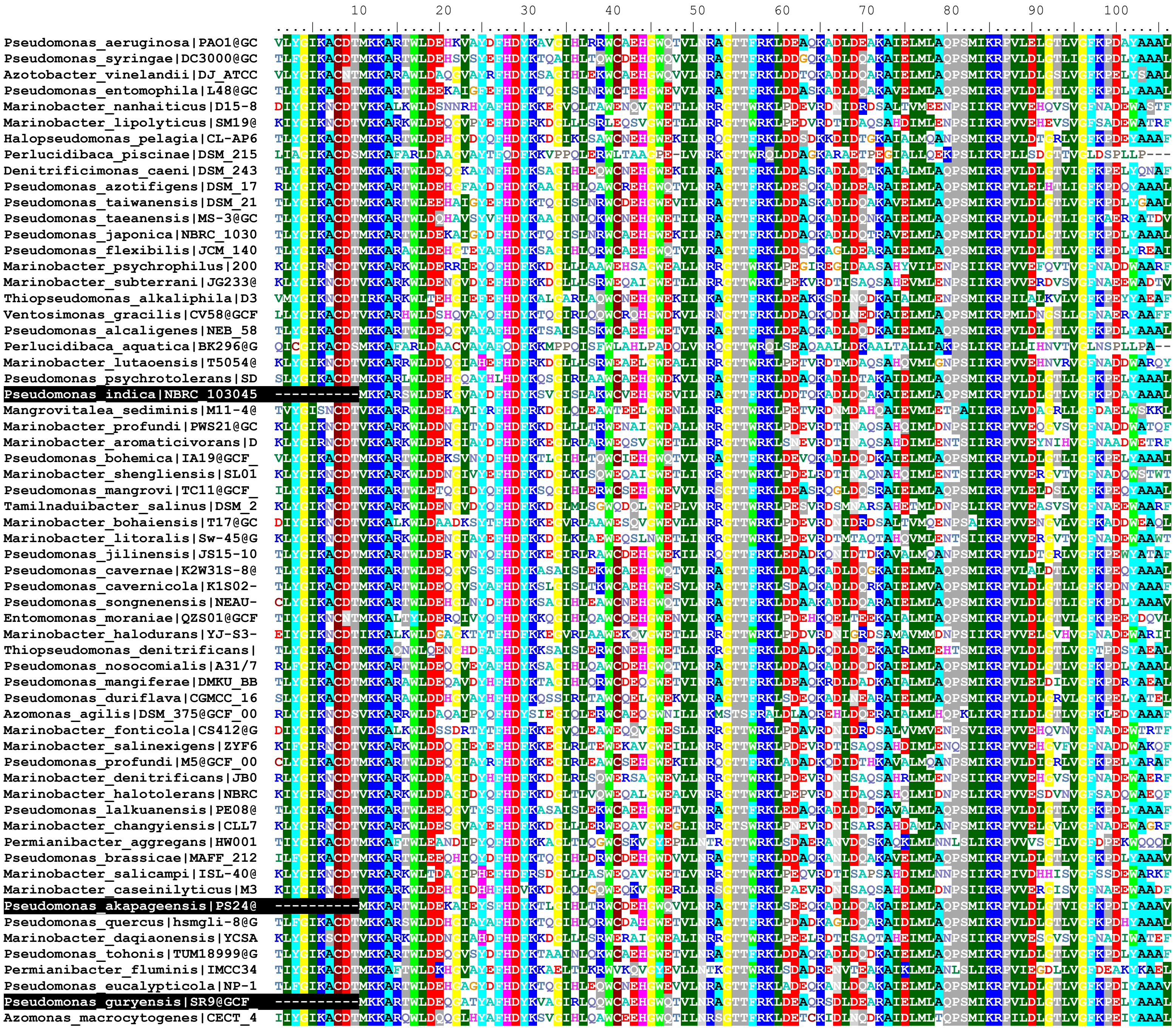
10 20 30 40 50 60 70 80 90 100
Pseudomonas_aeruginosa|PAO1@GC
Pseudomonas_syringae|DC3000@GC
Azotobacter_vinelandii|DJ_ATCC
Pseudomonas_entomophila|L48@GC
Marinobacter_nanhaiticus|D15-8
Marinobacter_lipolyticus|SM19@
Halopseudomonas_pelagia|CL-AP6
Perlucidibaca_piscinae|DSM_215
Denitrificimonas_caeni|DSM_243
Pseudomonas_azotifigens|DSM_17
Pseudomonas_taiwanensis|DSM_21
Pseudomonas_taeanensis|MS-3@GC
Pseudomonas_japonica|NBRC_1030
Pseudomonas_flexibilis|JCM_140
Marinobacter_psychrophilus|200
Marinobacter_subterrani|JG233@
Thiopseudomonas_alkaliphila|D3
Ventosimonas_gracilis|CV58@GCF
Pseudomonas_alcaligenes|NEB_58
Perlucidibaca_aquatica|BK296@G
Marinobacter_lutaoensis|T5054@
Pseudomonas_psychrotolerans|SD
Pseudomonas_indica|NBRC_103045
Mangrovitalea_sediminis|M11-4@
Marinobacter_profundi|PWS21@GC
Marinobacter_aromaticivorans|D
Pseudomonas_bohemica|IA19@GCF_
Marinobacter_shengliensis|SL01
Pseudomonas_mangrovi|TC11@GCF_
Tamilnaduibacter_salinus|DSM_2
Marinobacter_bohaiensis|T17@GC
Marinobacter_litoralis|Sw-45@G
Pseudomonas_jilinensis|JS15-10
Pseudomonas_cavernae|K2W31S-8@
Pseudomonas_cavernicola|K1S02-
Pseudomonas_songnenensis|NEAU-
Entomomonas_moraniae|QZS01@GCF
Marinobacter_halodurans|YJ-S3-
Thiopseudomonas_denitrificans|
Pseudomonas_nosocomialis|A31/7
Pseudomonas_mangiferae|DMKU_BB
Pseudomonas_duriflava|CGMCC_16
Azomonas_agilis|DSM_375@GCF_00
Marinobacter_fonticola|CS412@G
Marinobacter_salinexigens|ZYF6
Pseudomonas_profundi|M5@GCF_00
Marinobacter_denitrificans|JB0
Marinobacter_halotolerans|NBRC
Pseudomonas_lalkuanensis|PE08@
Marinobacter_changyiensis|CLL7
Permianibacter_aggregans|HW001
Pseudomonas_brassicae|MAFF_212
Marinobacter_salicampi|ISL-40@
Marinobacter_caseinilyticus|M3
Pseudomonas_akapageensis|PS24@
Pseudomonas_quercus|hsmgli-8@G
Marinobacter_daqiaonensis|YCSA
Pseudomonas_tohonis|TUM18999@G
Permianibacter_fluminis|IMCC34
Pseudomonas_eucalypticola|NP-1
Pseudomonas_guryensis|SR9@GCF_
Azomonas_macrocytogenes|CECT_4

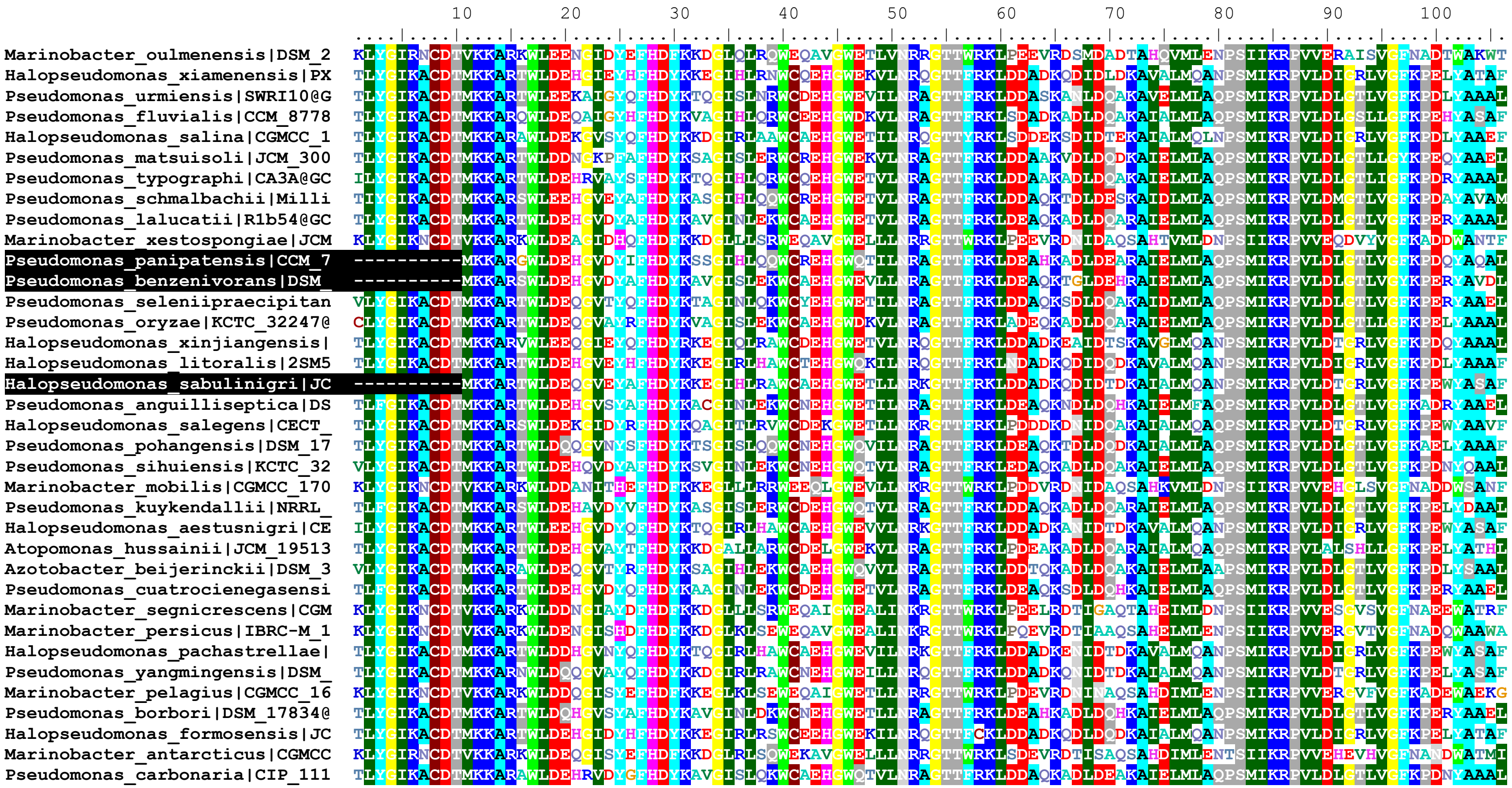

Figure S20. Trimmed multiple alignment of the Q9HXX5 protein family. A deletion is present between position 1 and 10 (in black) in the sequences of *Pseudomonas indica*, *Pseudomonas akapageensis*, *Pseudomonas guryensis*, *Pseudomonas panipatensis Pseudomonas benzenivorans*, and *Halopseudomonas sabulinigris*.

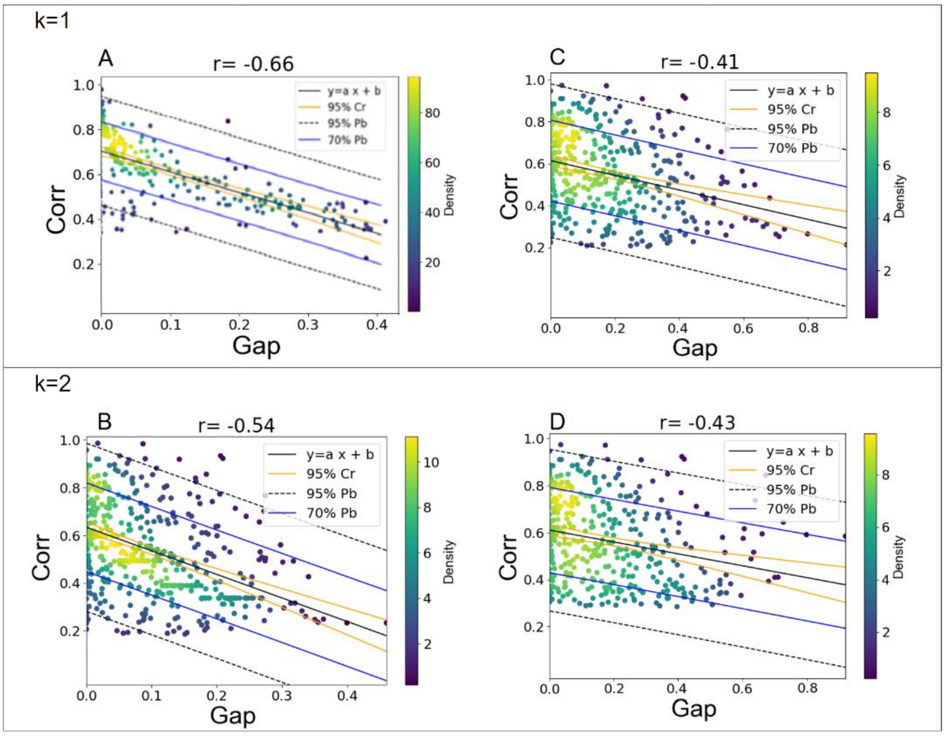

Figure S21. Effect of indels on correlations between ML-distances and normalized WS-distances. Each plot contains 518 points, each corresponding to a protein family. **(A)** Comparison between the amount of gaps (x-axis) and coefficient correlations between normalized Ws-distances in dimension 1 and ML-distances (y-axis) (r = -0.66, p-value = $5x10^{-4}$), **(B)** Comparison between the amount of gaps (x-axis) and coefficient correlations between normalized Ws-distances in dimension 2 and ML-distances (y-axis) (r = -0.54, p-value = $2x10^{-4}$), **(C)** Comparison between the amount of gaps (x-axis) and coefficient correlations between normalized Ws-distances in dimension 1 and p-distances (y-axis) (r = -0.41, p-value = $9x10^{-3}$), and **(D)** Comparison between the amount of gaps (x-axis) and coefficient correlations between normalized Ws-distances in dimension 2 and p-distances (y-axis) (r = -0.43, p-value = $8x10^{-4}$). The amount of gaps is computed as the number of gaps in the trimmed multiple alignments used to infer ML-trees divided by the length of the multiple alignment and the number of sequences **(A-B)** and the number of gaps in the untrimmed multiple alignments used to compute p-distances divided by the length of the multiple alignment and the number of sequences **(C-D)**. Normalized Ws-distances were computed with the AC filtration and by considering all Cα, including those corresponding to indels. For each panel, the orange line is the 95% confidence regions in which the true regression line should belong. The solid light blue lines are the 70% interval within which future individual data points or observations should fall. The dashed dark blue lines are the 95% interval within which a future individual data point or observation should fall. The black line corresponds to the regression line (y=ax+b). Point colors correspond to the density values according to the density scale.

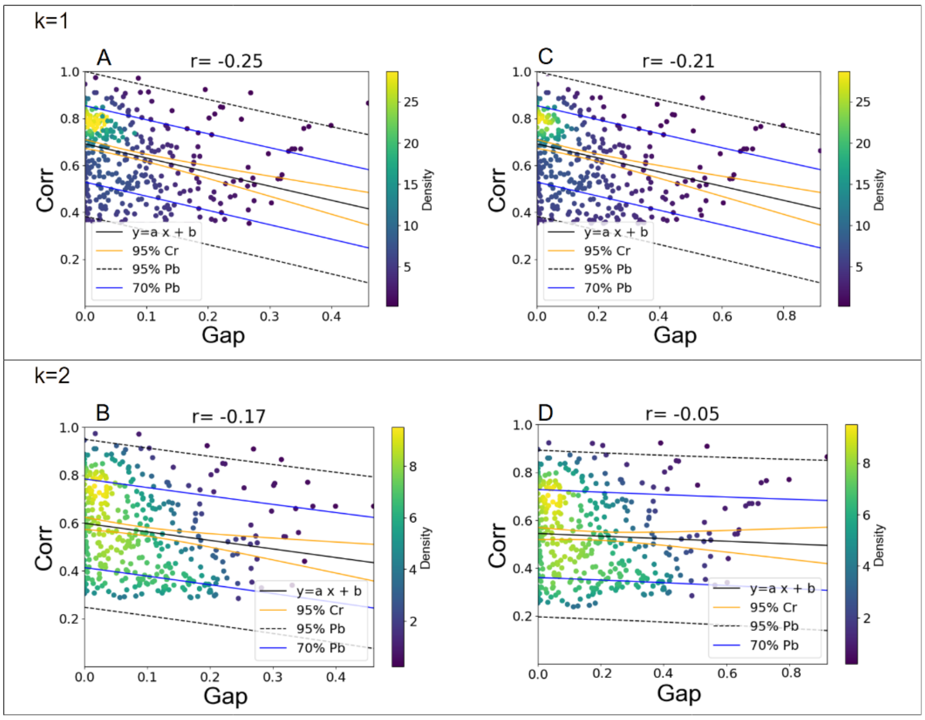

Figure S22. Effect of the removal indels on correlations between ML-distances and normalized WS-distances. Each plot contains 518 points, each corresponding to a protein family. **(A)** Comparison between the amount of gaps (x-axis) and coefficient correlations between normalized Ws-distances in dimension 1 and ML-distances (y-axis) (r = -0.25, p-value=0.5), **(B)** Comparison between the amount of gaps (x-axis) and coefficient correlations between normalized Ws-distances in dimension 2 and ML-distances (y-axis) (r = -0.17, p-value = 0.7), **(C)** Comparison between the amount of gaps (x-axis) and coefficient correlations between normalized Ws-distances in dimension 1 and p-distances (y-axis) (r = -0.21, p-value = 0.12), and **(D)** Comparison between the amount of gaps (x-axis) and coefficient correlations between normalized Ws-distances in dimension 2 and p-distances (y-axis) (r = -0.05, p-value = 0.2). The amount of gaps is computed as the number of gaps in the trimmed multiple alignments used to infer ML-trees divided by the length of the multiple alignment and the number of sequences **(A-B)** and the number of gaps in the untrimmed multiple alignments used to compute p-distances divided by the length of the multiple alignment and the number of sequences **(C-D)**. Normalized Ws-distances were computed with the AC filtration and by omitting Cα corresponding to indels. For each panel, the orange line is the 95% confidence regions in which the true regression line should belong. The solid light blue lines are the 70% interval within which future individual data points or observations should fall. The dashed dark blue lines are the 95% interval within which a future individual data point or observation should fall. The black line corresponds to the regression line (y=ax+b). Point colors correspond to the density values according to the density scale.

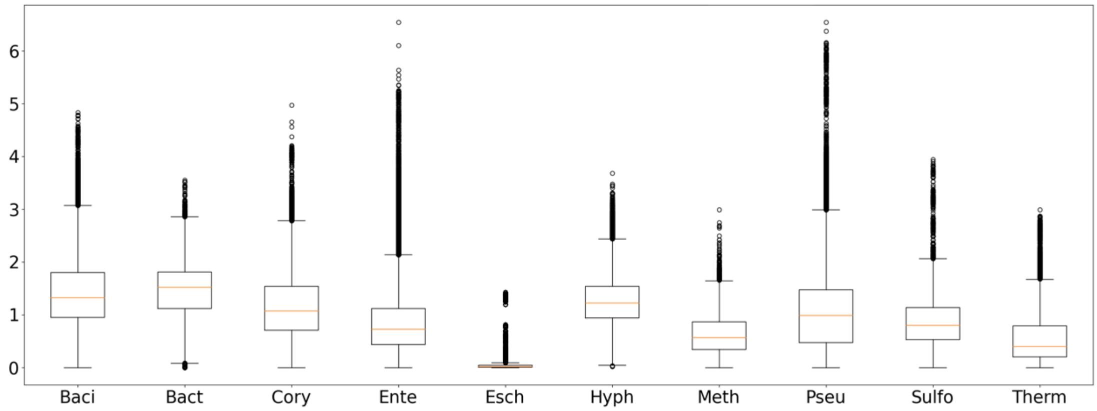

Taxa	Lower whisker	Lower quartile	Median	Upper quartile	Upper whisker
Bacillales	0.00	0.95	1.33	1.80	3.07
Bacteroidales	0.08	1.12	1.53	1.82	2.85
Corynebacteriales	0.00	0.71	1.07	1.54	2.78
Enterobacterales	0.00	0.45	0.73	1.13	2.15
Escherichia	0.00	0.01	0.02	0.04	0.10
Hyphomicrobiales	0.05	0.95	1.28	1.54	2.44
Methanococcales	0.00	0.34	0.57	0.86	1.65
Pseudomonadales	0.00	0.47	0.99	1.48	2.99
Sulfolobales	0.00	0.53	0.80	1.14	2.06
Thermococcales	0.00	0.21	0.40	0.79	1.68

Figure S23. Boxplots showing the distribution of ML-distances for each of the ten taxa analyzed. The x-axis corresponds to taxa, the y-axis to ML-distances computed for each pair of homologous protein sequences. Baci: *Bacillales*, Bact: *Bacteroidales*, Cory: *Corynebacteriales*, Ente: *Enterobacterales*, Esch: *Escherichia*, Hyph: *Hyphomicrobiales*, Meth: *Methanococcales*, Pseu: *Pseudomonadales*, Sulfo: *Sulfolobales*, and Ther: *Thermococcales*. Metrics for each distribution are summarized in the table.

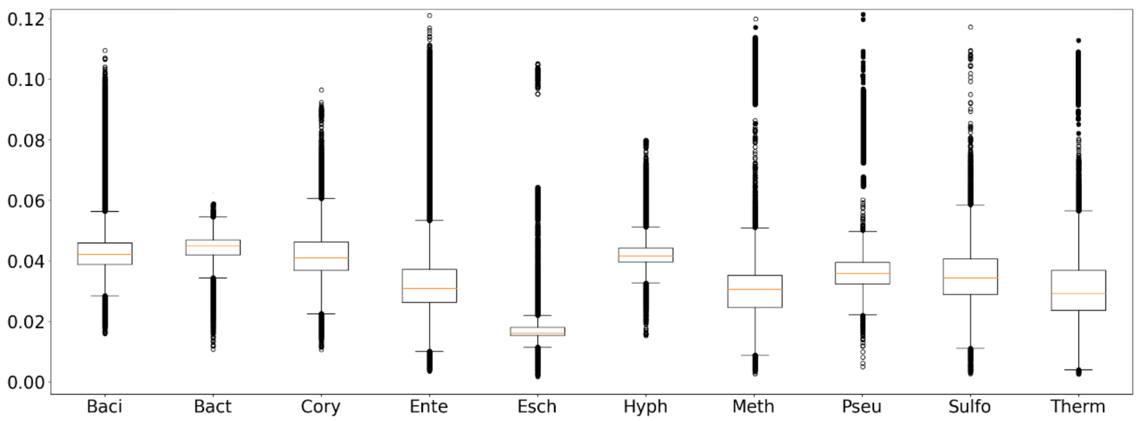

Taxa	Lower whisker	Lower quartile	Median	Upper quartile	Upper whisker
Bacillales	0.028	0.039	0.042	0.046	0.056
Bacteroidales	0.035	0.04	0.049	0.058	0.06
Corynebacteriales	0.022	0.037	0.041	0.046	0.061
Enterobacterales	0.01	0.026	0.031	0.037	0.053
Escherichia	0.011	0.015	0.016	0.018	0.022
Hyphomicrobiales	0.033	0.04	0.042	0.044	0.051
Methanococcales	0.009	0.025	0.031	0.035	0.051
Pseudomonadales	0.022	0.032	0.036	0.039	0.05
Sulfolobales	0.011	0.029	0.034	0.041	0.058
Thermococcales	0.004	0.024	0.029	0.037	0.056

Figure S24. Boxplots showing the distribution of normalized Ws-distances in homological dimension 1 for each of the ten taxa analyzed. The x-axis corresponds to taxa, the y-axis to Ws1-distances calculated for each pair of homologous proteins by AC filtration of PC(Cα) with a CI >70% and not involved in indels. Baci: *Bacillales*, Bact: *Bacteroidales*, Cory: *Corynebacteriales*, Ente: *Enterobacterales*, Esch: *Escherichia*, Hyph: *Hyphomicrobiales*, Meth: *Methanococcales*, Pseu: *Pseudomonadales*, Sulfo: *Sulfolobales*, and Ther: *Thermococcales*. Metrics for each distribution are summarized in the table.

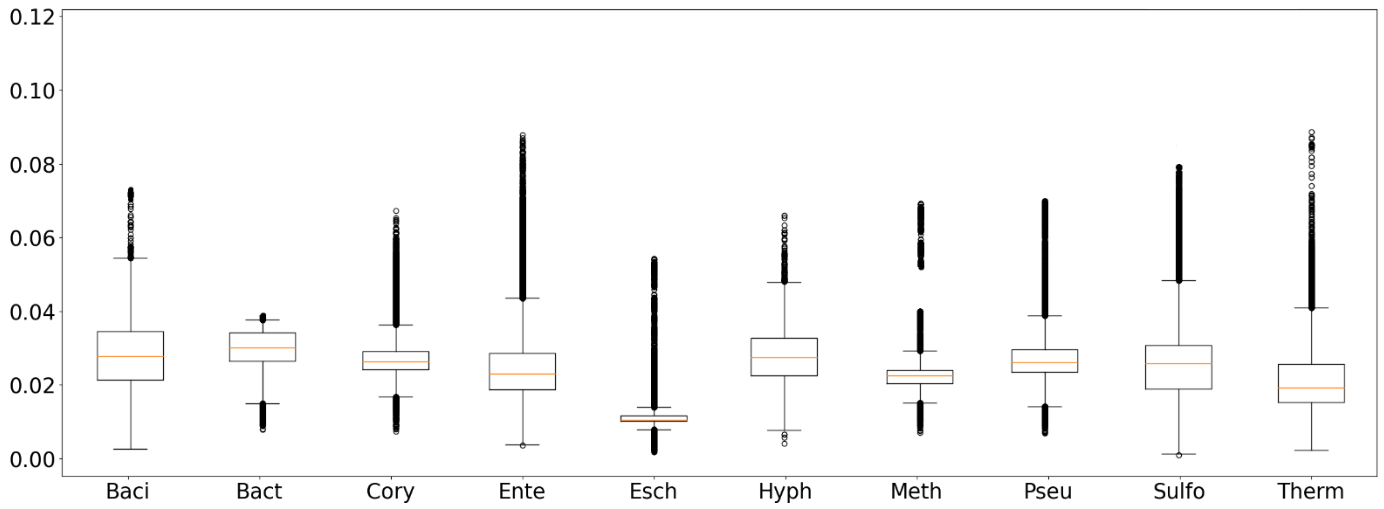

Taxa	Lower whisker	Lower quartile	Median	Upper quartile	Upper whisker
Bacillales	0.003	0.021	0.028	0.035	0.054
Bacteroidales	0.016	0.027	0.031	0.035	0.047
Corynebacteriales	0.017	0.024	0.026	0.029	0.036
Enterobacterales	0.004	0.019	0.023	0.029	0.044
Escherichia	0.008	0.010	0.011	0.012	0.014
Hyphomicrobiales	0.008	0.023	0.027	0.033	0.048
Methanococcales	0.015	0.020	0.023	0.024	0.029
Pseudomonadales	0.014	0.023	0.026	0.030	0.039
Sulfolobales	0.001	0.019	0.026	0.031	0.048
Thermococcales	0.002	0.015	0.019	0.026	0.041

Figure S25. Boxplots showing the distribution of normalized Ws-distances in homological dimension 2 for each of the ten taxa analyzed. The x-axis corresponds to taxa, the y-axis to Ws2-distances calculated for each pair of homologous proteins by AC filtration of PC(Cα) with a CI >70% and not involved in indels. Baci: *Bacillales*, Bact: *Bacteroidales*, Cory: *Corynebacteriales*, Ente: *Enterobacterales*, Esch: *Escherichia*, Hyph: *Hyphomicrobiales*, Meth: *Methanococcales*, Pseu: *Pseudomonadales*, Sulfo: *Sulfolobales*, and Ther: *Thermococcales*. Metrics for each distribution are summarized in the table.

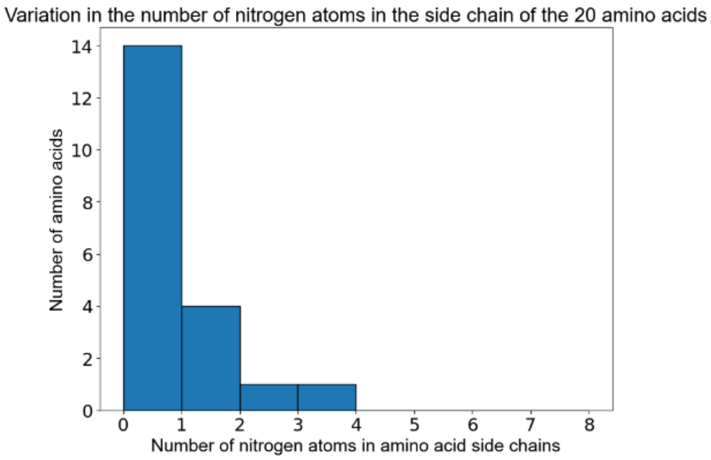

Figure S26. Histogram showing the variation in the number of nitrogen atoms in the side chain of the 20 amino acids. The x-axis corresponds to the number of nitrogen atoms in the side chains, the y-axis to the number of amino acids. The majority of amino acids (14 / 20amino acids) have no nitrogen atom in their side chains.

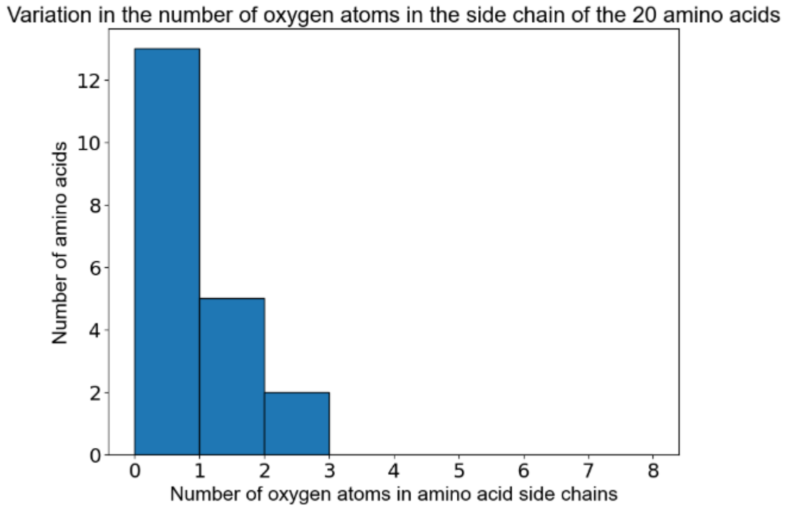

Figure S27. Histogram showing the variation in the number of oxygen atoms in the side chain of the 20 amino acids. The x-axis corresponds to the number of oxygen atoms in the side chains, the y-axis corresponds to the number of amino acids. The majority of amino acids (13 / 20 amino acids) have no oxygen atom in their side chains.

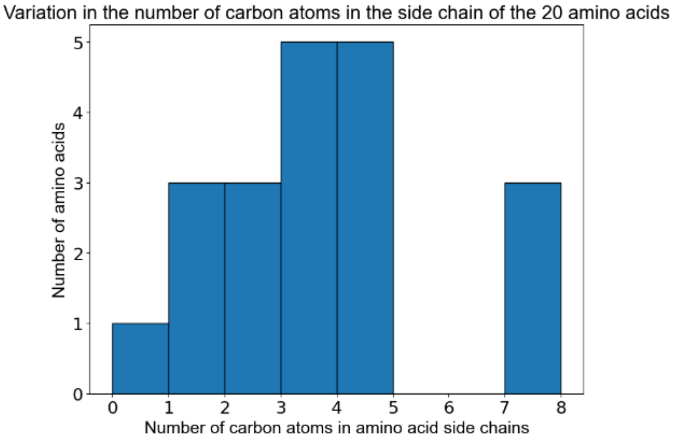

Figure S28. Histogram showing the variation in the number of carbon atoms in the side chain of 20 amino acids. The x-axis corresponds to the number of carbon atoms in the side chains, the y-axis corresponds to the number of amino acids. The number of carbons in side chains is more variable from one amino acid to another than the number of nitrogen or oxygen atoms.

Supplementary Tables

Table S1. Table showing the correlation coefficients between PH-distances and the average number of points in PC(Cα) being compared. PH-distances (Btk-distances, Ws-distances, and Ls-distances) were calculated on PC(Cα) for each of the 763,648 pairs of homologous protein structures predicted by Alphafold2. Normalized (Norm.) PH-distances were calculated by dividing PH-distances by the average number of points in the two PC(Cα) being compared. VR: Vietoris-Rips filtration, AC: Alpha complex filtration, k=1: homological dimension 1, k=2: homological dimension 2, r: correlation coefficient, p: p-value. p-values are significant when PH-distances are not normalized, while they are not significant when PH-distances are normalized.

Filtration	Btk-dists	Ws-dists	Ls-dists	Norm. Btk-dists	Norm. Ws-dists	Norm. Ls-dists
**VR / k=1**	$r = 0.39$ / $p = 0.2 \times 10^{-5}$	$r = 0.44$ / $p = 3.1 \times 10^{-5}$	$r = 0.41$ / $p = 7.7 \times 10^{-5}$	$r = -0.09$ / $p = 0.2$	$r = -0.08$ / $p = 0.3$	$r = -0.003$ / $p = 0.6$
**VR / k=2**	$r = 0.46$ / $p = 5.0 \times 10^{-4}$	$r = 0.49$ / $p = 6.0 \times 10^{-4}$	$r = 0.47$ / $p = 3.8 \times 10^{-3}$	$r = -0.10$ / $p = 0.2$	$r = -0.09$ / $p = 0.2$	$r = -0.11$ / $p = 0.1$
**AC / k=1**	$r = 0.43$ / $p = 7.3 \times 10^{-4}$	$r = 0.51$ / $p = 2.0 \times 10^{-5}$	$r = 0.47$ / $p = 5.1 \times 10^{-5}$	$r = -0.11$ / $p = 0.2$	$r = -0.06$ / $p = 0.1$	$r = -0.08$ / $p = 0.5$
**AC / k=2**	$r = 0.50$ / $p = 1.7 \times 10^{-5}$	$r = 0.61$ / $p = 1.7 \times 10^{-5}$	$r = 0.53$ / $p = 1.7 \times 10^{-5}$	$r = -0.002$ / $p = 0.9$	$r = -0.0005$ / $p = 0.1$	$r = -0.004$ / $p = 0.8$

Table S2. Table showing the correlation coefficients between EV- and normalized PH-distances computed from PC(Cα). For each of the 736,648 pairs of homologous protein structures predicted by Alphafold2, EV-distances were computed by from protein sequence alignments (p-distances) and ML trees (ML-distances), whereas PH-distances (Btk-distances, Ws-distances, and Ls-distances) were calculated by filtering PC(Cα) from Alphafold2 predictions. VR: Vietoris-Rips filtration, AC: Alpha complex filtration, k=1: homological dimension 1, k=2: homological dimension 2, r: correlation coefficient, p: p-value.

Filtration	Btk / p-dists	Btk / ML-dists	Ws / p-dists	Ws / ML-dists	Ls / p-dists	Ls / ML-dists
**VR / k=1**	$r = 0.44$ / $p = 2x10^{-5}$	$r = 0.45$ / $p = 3x10^{-3}$	$r = 0.64$ / $p = 7x10^{-3}$	$r = 0.67$ / $p = 6x10^{-3}$	$r = 0.64$ / $p = 4x10^{-5}$	$r = 0.65$ / $p = 2x10^{-4}$
**VR / k=2**	$r = 0.43$ / $p = 4x10^{-4}$	$r = 0.44$ / $p = 2x10^{-4}$	$r = 0.63$ / $p = 2x10^{-4}$	$r = 0.65$ / $p = 1x10^{-5}$	$r = 0.61$ / $p = 8x10^{-3}$	$r = 0.62$ / $p = 6x10^{-4}$
**AC / k=1**	$r = 0.48$ / $p = 1x10^{-4}$	$r = 0.48$ / $p = 7x10^{-4}$	$r = 0.70$ / $p = 3x10^{-4}$	$r = 0.72$ / $p = 5x10^{-4}$	$r = 0.65$ / $p = 4x10^{-5}$	$r = 0.66$ / $p = 1x10^{-4}$
**AC / k=2**	$r = 0.45$ / $p = 3x10^{-5}$	$r = 0.47$ / $p = 6x10^{-4}$	$r = 0.72$ / $p = 3x10^{-5}$	$r = 0.74$ / $p = 2x10^{-4}$	$r = 0.62$ / $p = 6x10^{-4}$	$r = 0.64$ / $p = 4x10^{-5}$

Table S3. List of the ten prokaryote taxonomic groups used in this work. For each of them, the number of protein families, the list of species studied, corresponding genome accession, and taxonomy are provided.

Taxonomic group	Nb of protein family	Species	Genome accession	Taxonomy
**BACILLALES**	63	Anoxybacillus_thermarum|AF04	GCF_000836725.1	Bacteria-Firmicutes-Bacilli-Bacillales-Bacillaceae-Anoxybacillus-Anoxybacillus_thermarum
		Geomicrobium_sediminis|DSM25540	GCF_016908595.1	Bacteria-Firmicutes-Bacilli-Bacillales-no_family-Geomicrobium-Geomicrobium_sediminis
		Lysinibacillus_cavernae|SYSUK30005	GCF_009724685.1	Bacteria-Firmicutes-Bacilli-Bacillales-Bacillaceae-Lysinibacillus-Lysinibacillus_cavernae
		Virgibacillus_doumboii|Marseille_Q1616	GCF_902806455.1	Bacteria-Firmicutes-Bacilli-Bacillales-Bacillaceae-Virgibacillus-Virgibacillus_doumboii
		Anaerobacillus_alkalidiazotrophicus|DSM22531	GCF_001866055.1	Bacteria-Firmicutes-Bacilli-Bacillales-Bacillaceae-Anaerobacillus-Anaerobacillus_alkalidiazotrophicus
		Salsuginibacillus_kocurii|DSM18087	GCF_000377705.1	Bacteria-Firmicutes-Bacilli-Bacillales-Bacillaceae-Salsuginibacillus-Salsuginibacillus_kocurii
		Aneurinibacillus_soli|CB4	GCF_002355375.1	Bacteria-Firmicutes-Bacilli-Bacillales-Paenibacillaceae-Aneurinibacillus-Aneurinibacillus_soli
		Exiguobacterium_algae|S126	GCF_018617875.1	Bacteria-Firmicutes-Bacilli-Bacillales-no_family-Exiguobacterium-Exiguobacterium_algae
		Exiguobacterium_flavidum|HF60	GCF_003344535.1	Bacteria-Firmicutes-Bacilli-Bacillales-no_family-Exiguobacterium-Exiguobacterium_flavidum
		Polycladomyces_abyssicola|JIR_001	GCF_018326425.1	Bacteria-Firmicutes-Bacilli-Bacillales-Thermoactinomycetaceae-Polycladomyces-Polycladomyces_abyssicola
		Desulfuribacillus_alkaliarsenatis|AHT28	GCF_001730225.1	Bacteria-Firmicutes-Bacilli-Bacillales-no_family-Desulfuribacillus-Desulfuribacillus_alkaliarsenatis
		Texcoconibacillus_texcoconensis|DSM24696	GCF_014202575.1	Bacteria-Firmicutes-Bacilli-Bacillales-Bacillaceae-Texcoconibacillus-Texcoconibacillus_texcoconensis
		Ornithinibacillus_scapharcae|TW25	GCF_000190475.1	Bacteria-Firmicutes-Bacilli-Bacillales-Bacillaceae-Ornithinibacillus-Ornithinibacillus_scapharcae
		Longirhabdus_pacifica|SCSIO06110	GCF_004115085.1	Bacteria-Firmicutes-Bacilli-Bacillales-Paenibacillaceae-Longirhabdus-Longirhabdus_pacifica
		Salipaludibacillus_agaradhaerens|DSM8721	GCF_002019735.1	Bacteria-Firmicutes-Bacilli-Bacillales-Bacillaceae-Salipaludibacillus-Salipaludibacillus_agaradhaerens
		Peribacillus_kribbensis|DSM17871	GCF_000430765.1	Bacteria-Firmicutes-Bacilli-Bacillales-Bacillaceae-Peribacillus-Peribacillus_kribbensis
		Planococcus_koreensis|DSM15895	GCF_014202615.1	Bacteria-Firmicutes-Bacilli-Bacillales-Planococcaceae-Planococcus-Planococcus_koreensis
		Caldalkalibacillus_thermarum|TA2.A1	GCF_019856495.1	Bacteria-Firmicutes-Bacilli-Bacillales-Bacillaceae-Caldalkalibacillus-Caldalkalibacillus_thermarum
		Paenibacillus_contaminans|CKOBP_6	GCF_003285015.1	Bacteria-Firmicutes-Bacilli-Bacillales-Paenibacillaceae-Paenibacillus-Paenibacillus_contaminans
		Thermicanus_aegyptius|DSM12793	GCF_000510645.1	Bacteria-Firmicutes-Bacilli-Bacillales-Bacillales_Family_X._Incertae_Sedis-Thermicanus-Thermicanus_aegyptius
		Psychrobacillus_psychrodurans|Aquil_B6	GCF_022603175.1	Bacteria-Firmicutes-Bacilli-Bacillales-Bacillaceae-Psychrobacillus-Psychrobacillus_psychrodurans
		Peribacillus_butanolivorans|PHB_7a	GCF_003410415.1	Bacteria-Firmicutes-Bacilli-Bacillales-Bacillaceae-Peribacillus-Peribacillus_butanolivorans
		Halobacillus_massiliensis|Marseille_P3554	GCF_900166625.1	Bacteria-Firmicutes-Bacilli-Bacillales-Bacillaceae-Halobacillus-Halobacillus_massiliensis
		Massilibacterium_senegalense|mt8	GCF_001375675.1	Bacteria-Firmicutes-Bacilli-Bacillales-Bacillaceae-Massilibacterium-Massilibacterium_senegalense

Macrococcus_bohemicus|19Msa422	GCF_016865215.1	Bacteria-Firmicutes-Bacilli-Bacillales-Staphylococcaceae-Macrococcus-Macrococcus_bohemicus
Cytobacillus_luteolus|YIM93174	GCF_014982515.1	Bacteria-Firmicutes-Bacilli-Bacillales-Bacillaceae-Cytobacillus-Cytobacillus_luteolus
Alkalihalobacillus_lonarensis|25nlg	GCF_900096965.1	Bacteria-Firmicutes-Bacilli-Bacillales-Bacillaceae-Alkalihalobacillus-Alkalihalobacillus_lonarensis
Alicyclobacillus_tolerans|BL_1	GCF_021739485.1	Bacteria-Firmicutes-Bacilli-Bacillales-Alicyclobacillaceae-Alicyclobacillus-Alicyclobacillus_tolerans
Salinicoccus_hispanicus|J_82	GCF_009831115.1	Bacteria-Firmicutes-Bacilli-Bacillales-Staphylococcaceae-Salinicoccus-Salinicoccus_hispanicus
Mangrovibacillus_cuniculi|R1DC41	GCF_015482585.1	Bacteria-Firmicutes-Bacilli-Bacillales-Bacillaceae-Mangrovibacillus-Mangrovibacillus_cuniculi
Paenibacillus_campinasensis|7537_G1	GCF_002272015.1	Bacteria-Firmicutes-Bacilli-Bacillales-Paenibacillaceae-Paenibacillus-Paenibacillus_campinasensis
Thermoflavimicrobium_dichotomicum|DSM44778	GCF_900114055.1	Bacteria-Firmicutes-Bacilli-Bacillales-Thermoactinomycetaceae-Thermoflavimicrobium-Thermoflavimicrobium_dichotomicum
Paenibacillus_lutrae|N10	GCF_009757775.1	Bacteria-Firmicutes-Bacilli-Bacillales-Paenibacillaceae-Paenibacillus-Paenibacillus_lutrae
Aneurinibacillus_migulanus|DSM2895	GCF_001274715.1	Bacteria-Firmicutes-Bacilli-Bacillales-Paenibacillaceae-Aneurinibacillus-Aneurinibacillus_migulanus
Lottiidibacillus_patelloidae|SA5d_4	GCF_002262935.1	Bacteria-Firmicutes-Bacilli-Bacillales-Bacillaceae-Lottiidibacillus-Lottiidibacillus_patelloidae
Brevibacillus_parabrevis|B3	GCF_022701015.1	Bacteria-Firmicutes-Bacilli-Bacillales-Paenibacillaceae-Brevibacillus-Brevibacillus_parabrevis
Heyndrickxia_sporothermodurans|SAD	GCF_003055045.1	Bacteria-Firmicutes-Bacilli-Bacillales-Bacillaceae-Heyndrickxia-Heyndrickxia_sporothermodurans
Paenibacillus_swuensis|DY6	GCF_001644605.1	Bacteria-Firmicutes-Bacilli-Bacillales-Paenibacillaceae-Paenibacillus-Paenibacillus_swuensis
Alkalihalobacillus_berkeleyi|KCTC12718	GCF_021608225.1	Bacteria-Firmicutes-Bacilli-Bacillales-Bacillaceae-Alkalihalobacillus-Alkalihalobacillus_berkeleyi
Listeria_monocytogenes|EGD_e	GCF_000196035.1	Bacteria-Firmicutes-Bacilli-Bacillales-Listeriaceae-Listeria-Listeria_monocytogenes
Shimazuella_kribbensis|DSM45090	GCF_000428065.1	Bacteria-Firmicutes-Bacilli-Bacillales-Thermoactinomycetaceae-Shimazuella-Shimazuella_kribbensis
Pullulanibacillus_camelliae|CGMCC1.15371	GCF_014642655.1	Bacteria-Firmicutes-Bacilli-Bacillales-Sporolactobacillaceae-Pullulanibacillus-Pullulanibacillus_camelliae
Melghiribacillus_thermohalophilus|DSM25894	GCF_004342905.1	Bacteria-Firmicutes-Bacilli-Bacillales-Bacillaceae-Melghiribacillus-Melghiribacillus_thermohalophilus
Alicyclobacillus_suci|VF_FSL_W10_0049	GCF_017329615.1	Bacteria-Firmicutes-Bacilli-Bacillales-Alicyclobacillaceae-Alicyclobacillus-Alicyclobacillus_suci
Brochothrix_thermosphacta|TMW2.1564	GCF_001703515.1	Bacteria-Firmicutes-Bacilli-Bacillales-Listeriaceae-Brochothrix-Brochothrix_thermosphacta
Salisediminibacterium_selenitireducens|MLS9	GCF_000093085.1	Bacteria-Firmicutes-Bacilli-Bacillales-Bacillaceae-Salisediminibacterium-Salisediminibacterium_selenitireducens
Bacillus_kexueae|KCTC33881	GCF_022809095.1	Bacteria-Firmicutes-Bacilli-Bacillales-Bacillaceae-Bacillus-Bacillus_kexueae
Terribacillus_halophilus|DSM21620	GCF_900101385.1	Bacteria-Firmicutes-Bacilli-Bacillales-Bacillaceae-Terribacillus-Terribacillus_halophilus
Effusibacillus_lacus|DSM27172	GCF_004343345.1	Bacteria-Firmicutes-Bacilli-Bacillales-Alicyclobacillaceae-Effusibacillus-Effusibacillus_lacus
Jeotgalibacillus_soli|P9	GCF_000829435.1	Bacteria-Firmicutes-Bacilli-Bacillales-Planococcaceae-Jeotgalibacillus-Jeotgalibacillus_soli
Alkalihalobacillus_nanhaiisediminis|CGMCC1.10116	GCF_007830185.1	Bacteria-Firmicutes-Bacilli-Bacillales-Bacillaceae-Alkalihalobacillus-Alkalihalobacillus_nanhaiisediminis
Metabacillus_mangrovi|KCTC33872	GCF_009711125.1	Bacteria-Firmicutes-Bacilli-Bacillales-Bacillaceae-Metabacillus-Metabacillus_mangrovi
Salinibacillus_xinjiangensis|J4	GCF_009649735.1	Bacteria-Firmicutes-Bacilli-Bacillales-Bacillaceae-Salinibacillus-Salinibacillus_xinjiangensis

	Staphylococcus_aureus_subsp._aureus|NCTC8325	GCF_000013425.1	Bacteria-Firmicutes-Bacilli-Bacillales-Staphylococcaceae-Staphylococcus-Staphylococcus_aureus_subsp._aureus
	Chengkuizengella_marina|YPA3_1_1	GCF_009910845.1	Bacteria-Firmicutes-Bacilli-Bacillales-Paenibacillaceae-Chengkuizengella-Chengkuizengella_marina
	Bacillus_carboniphilus|SaN35_3	GCF_020524035.1	Bacteria-Firmicutes-Bacilli-Bacillales-Bacillaceae-Bacillus-Bacillus_carboniphilus
	Fictibacillus_aquaticus|GDSW_R2A3	GCF_002245695.1	Bacteria-Firmicutes-Bacilli-Bacillales-Bacillaceae-Fictibacillus-Fictibacillus_aquaticus
	Alkalicoccus_saliphilus|6AG	GCF_003044065.1	Bacteria-Firmicutes-Bacilli-Bacillales-Bacillaceae-Alkalicoccus-Alkalicoccus_saliphilus
	Ammoniphilus_oxalaticus|RAOx_1	GCF_003609605.1	Bacteria-Firmicutes-Bacilli-Bacillales-Paenibacillaceae-Ammoniphilus-Ammoniphilus_oxalaticus
	Acidibacillus_sulfuroxidans|Y002	GCF_003144315.1	Bacteria-Firmicutes-Bacilli-Bacillales-no_family-Acidibacillus-Acidibacillus_sulfuroxidans
	Alicyclobacillus_vulcanalis|DSM16176	GCF_900156755.1	Bacteria-Firmicutes-Bacilli-Bacillales-Alicyclobacillaceae-Alicyclobacillus-Alicyclobacillus_vulcanalis
	Niallia_circulans|FDAARGOS_783	GCF_013267435.1	Bacteria-Firmicutes-Bacilli-Bacillales-Bacillaceae-Niallia-Niallia_circulans
	Gemella_cuniculi|DSM15828	GCF_000425665.1	Bacteria-Firmicutes-Bacilli-Bacillales-no_family-Gemella-Gemella_cuniculi
	Pontibacillus_litoralis|JSM072002	GCF_000775615.1	Bacteria-Firmicutes-Bacilli-Bacillales-Bacillaceae-Pontibacillus-Pontibacillus_litoralis
	Microaerobacter_geothermalis|DSM22679	GCF_021608135.1	Bacteria-Firmicutes-Bacilli-Bacillales-Bacillaceae-Microaerobacter-Microaerobacter_geothermalis
	Kurthia_gibsonii|B83	GCF_003660425.1	Bacteria-Firmicutes-Bacilli-Bacillales-Planococcaceae-Kurthia-Kurthia_gibsonii
	Calditerricola_satsumensis|JCM14719	GCF_014646935.1	Bacteria-Firmicutes-Bacilli-Bacillales-Bacillaceae-Calditerricola-Calditerricola_satsumensis
	Thalassobacillus_devorans|CCM7282	GCF_014635125.1	Bacteria-Firmicutes-Bacilli-Bacillales-Bacillaceae-Thalassobacillus-Thalassobacillus_devorans
	Paenibacillus_nuruki|TI45_13ar	GCF_001721045.1	Bacteria-Firmicutes-Bacilli-Bacillales-Paenibacillaceae-Paenibacillus-Paenibacillus_nuruki
	Marinococcus_halophilus|Marseille_P3459	GCF_900166605.1	Bacteria-Firmicutes-Bacilli-Bacillales-Bacillaceae-Marinococcus-Marinococcus_halophilus
	Bacillus_subtilis_subsp._subtilis|168	GCF_000009045.1	Bacteria-Firmicutes-Bacilli-Bacillales-Bacillaceae-Bacillus-Bacillus_subtilis_subsp._subtilis
	Brevibacillus_marinus|SCSIO07484	GCF_003963515.1	Bacteria-Firmicutes-Bacilli-Bacillales-Paenibacillaceae-Brevibacillus-Brevibacillus_marinus
	Terrilactibacillus_laevilacticus|SK5_6	GCF_006553035.1	Bacteria-Firmicutes-Bacilli-Bacillales-Bacillaceae-Terrilactibacillus-Terrilactibacillus_laevilacticus
	Alteribacillus_iranensis|DSM23995	GCF_900113025.1	Bacteria-Firmicutes-Bacilli-Bacillales-Bacillaceae-Alteribacillus-Alteribacillus_iranensis
	Mammaliicoccus_vitulinus|FDAARGOS_1153	GCF_016725245.1	Bacteria-Firmicutes-Bacilli-Bacillales-Staphylococcaceae-Mammaliicoccus-Mammaliicoccus_vitulinus
	Hydrogenibacillus_schlegelii|MA48	GCF_001653195.1	Bacteria-Firmicutes-Bacilli-Bacillales-Bacillales_Family_X._Incertae_Sedis-Hydrogenibacillus-Hydrogenibacillus_schlegelii
	Ammoniphilus_resinae|DSM24738	GCF_017873965.1	Bacteria-Firmicutes-Bacilli-Bacillales-Paenibacillaceae-Ammoniphilus-Ammoniphilus_resinae
	Novibacillus_thermophilus|SG_1	GCF_002005165.1	Bacteria-Firmicutes-Bacilli-Bacillales-Thermoactinomycetaceae-Novibacillus-Novibacillus_thermophilus
	Exiguobacterium_indicum|HHS31	GCF_001939065.1	Bacteria-Firmicutes-Bacilli-Bacillales-no_family-Exiguobacterium-Exiguobacterium_indicum
	Pueribacillus_theae|T8	GCF_003097615.1	Bacteria-Firmicutes-Bacilli-Bacillales-Bacillaceae-Pueribacillus-Pueribacillus_theae
	Sporolactobacillus_putidus|JCM15325	GCF_014647035.1	Bacteria-Firmicutes-Bacilli-Bacillales-Sporolactobacillaceae-Sporolactobacillus-Sporolactobacillus_putidus
	Salicibibacter_cibarius|NKC5_3	GCF_016495725.1	Bacteria-Firmicutes-Bacilli-Bacillales-Bacillaceae-Salicibibacter-Salicibibacter_cibarius
	Lederbergia_wuyishanensis|CGMCC1.12709	GCF_022900255.1	Bacteria-Firmicutes-Bacilli-Bacillales-Bacillaceae-Lederbergia-Lederbergia_wuyishanensis

		Jeotgalicoccus_schoeneichii|CCM8667	GCF_014635565.1	Bacteria-Firmicutes-Bacilli-Bacillales-Staphylococcaceae-Jeotgalicoccus-Jeotgalicoccus_schoeneichii
		Melghirimyces_profundicolus|DSM45787	GCF_003054245.1	Bacteria-Firmicutes-Bacilli-Bacillales-Thermoactinomycetaceae-Melghirimyces-Melghirimyces_profundicolus
		Kyrpidia_tusciae|DSM2912	GCF_000092905.1	Bacteria-Firmicutes-Bacilli-Bacillales-Alicyclobacillaceae-Kyrpidia-Kyrpidia_tusciae
		Baia_soyae|DSM46831	GCF_004341445.1	Bacteria-Firmicutes-Bacilli-Bacillales-Thermoactinomycetaceae-Baia-Baia_soyae
		Gracilibacillus_ureilyticus|CGMCC1.7727	GCF_900111195.1	Bacteria-Firmicutes-Bacilli-Bacillales-Bacillaceae-Gracilibacillus-Gracilibacillus_ureilyticus
		Fredinandcohnia_humi|DSM16318	GCF_001439915.1	Bacteria-Firmicutes-Bacilli-Bacillales-Bacillaceae-Fredinandcohnia-Fredinandcohnia_humi
		Alkalibacillus_aidingensis|YIM98829	GCF_014595945.1	Bacteria-Firmicutes-Bacilli-Bacillales-Bacillaceae-Alkalibacillus-Alkalibacillus_aidingensis
		Tepidibacillus_fermentans|DSM23802	GCF_004342885.1	Bacteria-Firmicutes-Bacilli-Bacillales-Bacillaceae-Tepidibacillus-Tepidibacillus_fermentans
		Tumebacillus_permanentifrigoris|DSM18773	GCF_003148565.1	Bacteria-Firmicutes-Bacilli-Bacillales-Alicyclobacillaceae-Tumebacillus-Tumebacillus_permanentifrigoris
		Abyssicoccus_albus|S31	GCF_001989575.1	Bacteria-Firmicutes-Bacilli-Bacillales-Staphylococcaceae-Abyssicoccus-Abyssicoccus_albus
		Listeria_rocourtiae|CECT7972	GCF_004363965.1	Bacteria-Firmicutes-Bacilli-Bacillales-Listeriaceae-Listeria-Listeria_rocourtiae
		Alteribacillus_bidgolensis|DSM25260	GCF_002886255.1	Bacteria-Firmicutes-Bacilli-Bacillales-Bacillaceae-Alteribacillus-Alteribacillus_bidgolensis
		Halobacillus_kuroshimensis|DSM18393	GCF_000425705.1	Bacteria-Firmicutes-Bacilli-Bacillales-Bacillaceae-Halobacillus-Halobacillus_kuroshimensis
		Caldalkalibacillus_mannanilyticus|JCM10596	GCF_000615945.1	Bacteria-Firmicutes-Bacilli-Bacillales-Bacillaceae-Caldalkalibacillus-Caldalkalibacillus_mannanilyticus
		Bacillus_alkalisoli|FJAT_45122	GCF_002797415.1	Bacteria-Firmicutes-Bacilli-Bacillales-Bacillaceae-Bacillus-Bacillus_alkalisoli
		Rubeoparvulum_massiliense|mt6	GCF_001049895.1	Bacteria-Firmicutes-Bacilli-Bacillales-Bacillaceae-Rubeoparvulum-Rubeoparvulum_massiliense
		Alicyclobacillus_mengziensis|S30H14	GCF_017298635.1	Bacteria-Firmicutes-Bacilli-Bacillales-Alicyclobacillaceae-Alicyclobacillus-Alicyclobacillus_mengziensis
**BACTEROIDALES**	31	Porphyromonas_levii|DD27	GCF_018206105.1	Bacteria-Bacteroidetes-Bacteroidia-Bacteroidales-Porphyromonadaceae-Porphyromonas-Porphyromonas_levii
		Seramator_thermalis|SYSUGA16112	GCF_004138125.1	Bacteria-Bacteroidetes-Bacteroidia-Bacteroidales-Dysgonomonadaceae-Seramator-Seramator_thermalis
		Gallalistipes_aquisgranensis|DSM108975	GCF_014982715.1	Bacteria-Bacteroidetes-Bacteroidia-Bacteroidales-Rikenellaceae-Gallalistipes-Gallalistipes_aquisgranensis
		Bacteroides_congonensis|Marseille_P3132	GCF_900130125.1	Bacteria-Bacteroidetes-Bacteroidia-Bacteroidales-Bacteroidaceae-Bacteroides-Bacteroides_congonensis
		Bacteroides_mediterraneensis|Marseille_P2644	GCF_900128455.1	Bacteria-Bacteroidetes-Bacteroidia-Bacteroidales-Bacteroidaceae-Bacteroides-Bacteroides_mediterraneensis
		Muribaculum_intestinale|YL27	GCF_001688845.2	Bacteria-Bacteroidetes-Bacteroidia-Bacteroidales-Muribaculaceae-Muribaculum-Muribaculum_intestinale
		Prevotella_koreensis|KCOM3155	GCF_003977605.1	Bacteria-Bacteroidetes-Bacteroidia-Bacteroidales-Prevotellaceae-Prevotella-Prevotella_koreensis
		Paramuribaculum_intestinale|DSM100764	GCF_003024815.1	Bacteria-Bacteroidetes-Bacteroidia-Bacteroidales-Muribaculaceae-Paramuribaculum-Paramuribaculum_intestinale
		Bacteroides_graminisolvens|DSM19988	GCF_000428125.1	Bacteria-Bacteroidetes-Bacteroidia-Bacteroidales-Bacteroidaceae-Bacteroides-Bacteroides_graminisolvens
		Lentimicrobium_saccharophilum|TBC1	GCF_001192835.1	Bacteria-Bacteroidetes-Bacteroidia-Bacteroidales-Lentimicrobiaceae-Lentimicrobium-Lentimicrobium_saccharophilum
		Coprobacter_fastidiosus|NSB1	GCF_003634345.1	Bacteria-Bacteroidetes-Bacteroidia-Bacteroidales-Barnesiellaceae-Coprobacter-Coprobacter_fastidiosus
		Bacteroides_eggerthii|FDAARGOS_1221	GCF_016894205.1	Bacteria-Bacteroidetes-Bacteroidia-Bacteroidales-Bacteroidaceae-Bacteroides-Bacteroides_eggerthii

	Prevotella_corporis|DSM18810	GCF_000430525.1	Bacteria-Bacteroidetes-Bacteroidia-Bacteroidales-Prevotellaceae-Prevotella-Prevotella_corporis
	Parabacteroides_faecis|DSM102983	GCF_014199665.1	Bacteria-Bacteroidetes-Bacteroidia-Bacteroidales-Tannerellaceae-Parabacteroides-Parabacteroides_faecis
	Porphyromonas_levii|DD27	GCF_018206105.1	Bacteria-Bacteroidetes-Bacteroidia-Bacteroidales-Porphyromonadaceae-Porphyromonas-Porphyromonas_levii
	Parabacteroides_chartae|DSM24967	GCF_900168155.1	Bacteria-Bacteroidetes-Bacteroidia-Bacteroidales-Tannerellaceae-Parabacteroides-Parabacteroides_chartae
	Phocaeicola_barnesiae|DSM18169	GCF_000374585.1	Bacteria-Bacteroidetes-Bacteroidia-Bacteroidales-Bacteroidaceae-Phocaeicola-Phocaeicola_barnesiae
	Tannerella_forsythia|92A2	GCF_000238215.1	Bacteria-Bacteroidetes-Bacteroidia-Bacteroidales-Tannerellaceae-Tannerella-Tannerella_forsythia
	Bacteroides_ndongoniae|Marseille_P3108	GCF_900108345.1	Bacteria-Bacteroidetes-Bacteroidia-Bacteroidales-Bacteroidaceae-Bacteroides-Bacteroides_ndongoniae
	Phocaeicola_salanitronis|DSM18170	GCF_000190575.1	Bacteria-Bacteroidetes-Bacteroidia-Bacteroidales-Bacteroidaceae-Phocaeicola-Phocaeicola_salanitronis
	Bacteroides_caecigallinarum|An496	GCF_016901995.1	Bacteria-Bacteroidetes-Bacteroidia-Bacteroidales-Bacteroidaceae-Bacteroides-Bacteroides_caecigallinarum
	Odoribacter_splanchnicus|FDAARGOS_1579	GCF_020736405.1	Bacteria-Bacteroidetes-Bacteroidia-Bacteroidales-Odoribacteraceae-Odoribacter-Odoribacter_splanchnicus
	Porphyromonas_gingivicanis|COT_022OH1391	GCF_000769135.1	Bacteria-Bacteroidetes-Bacteroidia-Bacteroidales-Porphyromonadaceae-Porphyromonas-Porphyromonas_gingivicanis
	Culturomica_massiliensis|Marseille_P2698	GCF_900091655.1	Bacteria-Bacteroidetes-Bacteroidia-Bacteroidales-Odoribacteraceae-Culturomica-Culturomica_massiliensis
	Duncaniella_freteri|TLL_A3	GCF_004766125.1	Bacteria-Bacteroidetes-Bacteroidia-Bacteroidales-Muribaculaceae-Duncaniella-Duncaniella_freteri
	Phocaeicola_vulgatus|MG01_10	GCF_020885855.1	Bacteria-Bacteroidetes-Bacteroidia-Bacteroidales-Bacteroidaceae-Phocaeicola-Phocaeicola_vulgatus
	Porphyromonas_canoris|COT_108OH1224	GCF_000765975.1	Bacteria-Bacteroidetes-Bacteroidia-Bacteroidales-Porphyromonadaceae-Porphyromonas-Porphyromonas_canoris
	Alistipes_provencensis|Marseille_P2431	GCF_900083545.1	Bacteria-Bacteroidetes-Bacteroidia-Bacteroidales-Rikenellaceae-Alistipes-Alistipes_provencensis
	Prevotella_pleuritidis|F0068	GCF_000468135.1	Bacteria-Bacteroidetes-Bacteroidia-Bacteroidales-Prevotellaceae-Prevotella-Prevotella_pleuritidis
	Pseudoprevotella_muciniphila|E39	GCF_003265305.2	Bacteria-Bacteroidetes-Bacteroidia-Bacteroidales-Prevotellaceae-Pseudoprevotella-Pseudoprevotella_muciniphila
	Prevotella_colorans|DSM100333	GCF_003096815.1	Bacteria-Bacteroidetes-Bacteroidia-Bacteroidales-Prevotellaceae-Prevotella-Prevotella_colorans
	Prevotella_marshii|DSM16973	GCF_000146675.1	Bacteria-Bacteroidetes-Bacteroidia-Bacteroidales-Prevotellaceae-Prevotella-Prevotella_marshii
	Williamwhitmania_taraxaci|A7P_90m	GCF_900096565.1	Bacteria-Bacteroidetes-Bacteroidia-Bacteroidales-Williamwhitmaniaceae-Williamwhitmania-Williamwhitmania_taraxaci
	Porphyromonas_cangingivalis|NCTC12856	GCF_900638305.1	Bacteria-Bacteroidetes-Bacteroidia-Bacteroidales-Porphyromonadaceae-Porphyromonas-Porphyromonas_cangingivalis
	Salinivirga_cyanobacteriivorans|L21_Spi_D4	GCF_001443605.1	Bacteria-Bacteroidetes-Bacteroidia-Bacteroidales-Salinivirgaceae-Salinivirga-Salinivirga_cyanobacteriivorans
	Prevotella_brevis|P6B11	GCF_000621825.1	Bacteria-Bacteroidetes-Bacteroidia-Bacteroidales-Prevotellaceae-Prevotella-Prevotella_brevis
	Porphyromonas_macacae|DSM20710	GCF_000379945.1	Bacteria-Bacteroidetes-Bacteroidia-Bacteroidales-Porphyromonadaceae-Porphyromonas-Porphyromonas_macacae
	Duncaniella_muris|DSM103720	GCF_003024805.1	Bacteria-Bacteroidetes-Bacteroidia-Bacteroidales-Muribaculaceae-Duncaniella-Duncaniella_muris
	Porphyromonas_loveana|DSM28520	GCF_003096695.1	Bacteria-Bacteroidetes-Bacteroidia-Bacteroidales-Porphyromonadaceae-Porphyromonas-Porphyromonas_loveana

	Bacteroides_luti|DSM26991	GCF_900128905.1	Bacteria-Bacteroidetes-Bacteroidia-Bacteroidales-Bacteroidaceae-Bacteroides-Bacteroides_luti
	Bacteroides_propionicifaciens|DSM19291	GCF_000375405.1	Bacteria-Bacteroidetes-Bacteroidia-Bacteroidales-Bacteroidaceae-Bacteroides-Bacteroides_propionicifaciens
	Sodaliphilus_pleomorphus|Oil_RF_744_WCA_WT_10	GCF_009676955.1	Bacteria-Bacteroidetes-Bacteroidia-Bacteroidales-Muribaculaceae-Sodaliphilus-Sodaliphilus_pleomorphus
	Bacteroides_coprosuis|DSM18011	GCF_000212915.1	Bacteria-Bacteroidetes-Bacteroidia-Bacteroidales-Bacteroidaceae-Bacteroides-Bacteroides_coprosuis
	Porphyromonas_asaccharolytica|DSM20707	GCF_000212375.1	Bacteria-Bacteroidetes-Bacteroidia-Bacteroidales-Porphyromonadaceae-Porphyromonas-Porphyromonas_asaccharolytica
	Prevotella_dentalis|DSM3688	GCF_000242335.1	Bacteria-Bacteroidetes-Bacteroidia-Bacteroidales-Prevotellaceae-Prevotella-Prevotella_dentalis
	Porphyromonas_bennonis|DSM23058	GCF_000375645.1	Bacteria-Bacteroidetes-Bacteroidia-Bacteroidales-Porphyromonadaceae-Porphyromonas-Porphyromonas_bennonis
	Bacteroides_ihuae|Marseille_P2824	GCF_900104585.1	Bacteria-Bacteroidetes-Bacteroidia-Bacteroidales-Bacteroidaceae-Bacteroides-Bacteroides_ihuae
	Butyricimonas_synergistica|DSM23225	GCF_000379665.1	Bacteria-Bacteroidetes-Bacteroidia-Bacteroidales-Odoribacteraceae-Butyricimonas-Butyricimonas_synergistica
	Bacteroides_caecicola|An768	GCF_016900695.1	Bacteria-Bacteroidetes-Bacteroidia-Bacteroidales-Bacteroidaceae-Bacteroides-Bacteroides_caecicola
	Lascolabacillus_massiliensis|SIT8	GCF_001282625.1	Bacteria-Bacteroidetes-Bacteroidia-Bacteroidales-Porphyromonadaceae-Lascolabacillus-Lascolabacillus_massiliensis
	Porphyromonas_catoniae|F0037	GCF_000318215.2	Bacteria-Bacteroidetes-Bacteroidia-Bacteroidales-Porphyromonadaceae-Porphyromonas-Porphyromonas_catoniae
	Bacteroides_pyogenes|KG_29	GCF_008121405.1	Bacteria-Bacteroidetes-Bacteroidia-Bacteroidales-Bacteroidaceae-Bacteroides-Bacteroides_pyogenes
	Prevotella_multiformis|F0096	GCF_018127985.1	Bacteria-Bacteroidetes-Bacteroidia-Bacteroidales-Prevotellaceae-Prevotella-Prevotella_multiformis
	Barnesiella_intestinihominis|YIT11860	GCF_000296465.1	Bacteria-Bacteroidetes-Bacteroidia-Bacteroidales-Barnesiellaceae-Barnesiella-Barnesiella_intestinihominis
	Prevotella_illustrans|A2931	GCF_017426725.1	Bacteria-Bacteroidetes-Bacteroidia-Bacteroidales-Prevotellaceae-Prevotella-Prevotella_illustrans
	Bacteroides_cellulosilyticus|CL06T03C01	GCF_018292125.1	Bacteria-Bacteroidetes-Bacteroidia-Bacteroidales-Bacteroidaceae-Bacteroides-Bacteroides_cellulosilyticus
	Perlabentimonas_gracilis|M08_MB	GCF_011174675.1	Bacteria-Bacteroidetes-Bacteroidia-Bacteroidales-Tenuifilaceae-Perlabentimonas-Perlabentimonas_gracilis
	Prevotella_bryantii|TS1_5	GCF_022024215.1	Bacteria-Bacteroidetes-Bacteroidia-Bacteroidales-Prevotellaceae-Prevotella-Prevotella_bryantii
	Alistipes_megaguti|Marseille_P5997	GCF_900604385.1	Bacteria-Bacteroidetes-Bacteroidia-Bacteroidales-Rikenellaceae-Alistipes-Alistipes_megaguti
	Alloprevotella_tannerae|ATCC51259	GCF_000159995.1	Bacteria-Bacteroidetes-Bacteroidia-Bacteroidales-Prevotellaceae-Alloprevotella-Alloprevotella_tannerae
	Microbacter_margulisiae|DSM27471	GCF_014192515.1	Bacteria-Bacteroidetes-Bacteroidia-Bacteroidales-Porphyromonadaceae-Microbacter-Microbacter_margulisiae
	Paraprevotella_clara|MGYG_HGUT_02540	GCF_902388125.1	Bacteria-Bacteroidetes-Bacteroidia-Bacteroidales-Prevotellaceae-Paraprevotella-Paraprevotella_clara
	Prevotella_multisaccharivorax|DSM17128	GCF_000218235.1	Bacteria-Bacteroidetes-Bacteroidia-Bacteroidales-Prevotellaceae-Prevotella-Prevotella_multisaccharivorax
	Dysgonomonas_macrotermitis|DSM27370	GCF_900128985.1	Bacteria-Bacteroidetes-Bacteroidia-Bacteroidales-Dysgonomonadaceae-Dysgonomonas-Dysgonomonas_macrotermitis
	Alloprevotella_rava|DSM22548	GCF_014195585.1	Bacteria-Bacteroidetes-Bacteroidia-Bacteroidales-Prevotellaceae-Alloprevotella-Alloprevotella_rava
	Porphyromonas_crevioricanis|NCTC12858	GCF_900476255.1	Bacteria-Bacteroidetes-Bacteroidia-Bacteroidales-Porphyromonadaceae-Porphyromonas-Porphyromonas_crevioricanis

Parabacteroides_pacaensis|Marseille_P4001	GCF_900292045.1	Bacteria-Bacteroidetes-Bacteroidia-Bacteroidales-Tannerellaceae-Parabacteroides-Parabacteroides_pacaensis
Prevotella_salivae|F0493	GCF_000477535.1	Bacteria-Bacteroidetes-Bacteroidia-Bacteroidales-Prevotellaceae-Prevotella-Prevotella_salivae
Porphyromonas_endodontalis|NCTC13058	GCF_900454815.1	Bacteria-Bacteroidetes-Bacteroidia-Bacteroidales-Porphyromonadaceae-Porphyromonas-Porphyromonas_endodontalis
Millionella_massiliensis|Marseille_P3215	GCF_900104655.1	Bacteria-Bacteroidetes-Bacteroidia-Bacteroidales-Rikenellaceae-Millionella-Millionella_massiliensis
Prevotellamassilia_timonensis|Marseille_P2831	GCF_900106785.1	Bacteria-Bacteroidetes-Bacteroidia-Bacteroidales-Prevotellaceae-Prevotellamassilia-Prevotellamassilia_timonensis
Balneicella_halophila|DSM28579	GCF_003096835.1	Bacteria-Bacteroidetes-Bacteroidia-Bacteroidales-Balneicellaceae-Balneicella-Balneicella_halophila
Parabacteroides_bouchesdurhonensis|Marseille_P3763	GCF_900186615.1	Bacteria-Bacteroidetes-Bacteroidia-Bacteroidales-Tannerellaceae-Parabacteroides-Parabacteroides_bouchesdurhonensis
Dysgonomonas_massiliensis|Marseille_P4356	GCF_900240225.1	Bacteria-Bacteroidetes-Bacteroidia-Bacteroidales-Dysgonomonadaceae-Dysgonomonas-Dysgonomonas_massiliensis
Proteiniphilum_saccharofermentans|M36	GCF_900095135.1	Bacteria-Bacteroidetes-Bacteroidia-Bacteroidales-Dysgonomonadaceae-Proteiniphilum-Proteiniphilum_saccharofermentans
Dysgonomonas_alginatilytica|DSM100214	GCF_003201355.1	Bacteria-Bacteroidetes-Bacteroidia-Bacteroidales-Dysgonomonadaceae-Dysgonomonas-Dysgonomonas_alginatilytica
Prevotella_nanceiensis|SCHI0011.S.12	GCF_019375915.1	Bacteria-Bacteroidetes-Bacteroidia-Bacteroidales-Prevotellaceae-Prevotella-Prevotella_nanceiensis
Paludibacter_jiangxiensis|NM7	GCF_001618385.1	Bacteria-Bacteroidetes-Bacteroidia-Bacteroidales-Paludibacteraceae-Paludibacter-Paludibacter_jiangxiensis
Alistipes_ihumii|AP11	GCF_000321205.1	Bacteria-Bacteroidetes-Bacteroidia-Bacteroidales-Rikenellaceae-Alistipes-Alistipes_ihumii
Phocaeicola_paurosaccharolyticus|JCM15092	GCF_000613805.1	Bacteria-Bacteroidetes-Bacteroidia-Bacteroidales-Bacteroidaceae-Phocaeicola-Phocaeicola_paurosaccharolyticus
Prevotella_lascolaii|khD1	GCF_900079775.1	Bacteria-Bacteroidetes-Bacteroidia-Bacteroidales-Prevotellaceae-Prevotella-Prevotella_lascolaii
Phocaeicola_abscessus|CCUG55929	GCF_000312445.1	Bacteria-Bacteroidetes-Bacteroidia-Bacteroidales-Bacteroidaceae-Phocaeicola-Phocaeicola_abscessus
Sanguibacteroides_justesenii|OUH969102	GCF_003204205.1	Bacteria-Bacteroidetes-Bacteroidia-Bacteroidales-Porphyromonadaceae-Sanguibacteroides-Sanguibacteroides_justesenii
Tannerella_serpentiformis|W11666	GCF_003033925.1	Bacteria-Bacteroidetes-Bacteroidia-Bacteroidales-Tannerellaceae-Tannerella-Tannerella_serpentiformis
Alistipes_communis|5CBH24	GCF_006542665.1	Bacteria-Bacteroidetes-Bacteroidia-Bacteroidales-Rikenellaceae-Alistipes-Alistipes_communis
Coprobacter_secundus_subsp._similis|2CBH44	GCF_015097275.1	Bacteria-Bacteroidetes-Bacteroidia-Bacteroidales-Barnesiellaceae-Coprobacter-Coprobacter_secundus_subsp._similis
Porphyromonas_somerae|CE91_St14	GCF_022845615.1	Bacteria-Bacteroidetes-Bacteroidia-Bacteroidales-Porphyromonadaceae-Porphyromonas-Porphyromonas_somerae
Acetobacteroides_hydrogenigenes|RL_C	GCF_004340205.1	Bacteria-Bacteroidetes-Bacteroidia-Bacteroidales-Rikenellaceae-Acetobacteroides-Acetobacteroides_hydrogenigenes
Dysgonomonas_mossii|DSM22836	GCF_000213575.1	Bacteria-Bacteroidetes-Bacteroidia-Bacteroidales-Dysgonomonadaceae-Dysgonomonas-Dysgonomonas_mossii
Alistipes_indistinctus|2BBH45	GCF_014163495.1	Bacteria-Bacteroidetes-Bacteroidia-Bacteroidales-Rikenellaceae-Alistipes-Alistipes_indistinctus
Porphyromonas_uenonis|DSM23387	GCF_000482365.1	Bacteria-Bacteroidetes-Bacteroidia-Bacteroidales-Porphyromonadaceae-Porphyromonas-Porphyromonas_uenonis
Prevotella_pectinovora|P5_125	GCF_000833995.1	Bacteria-Bacteroidetes-Bacteroidia-Bacteroidales-Prevotellaceae-Prevotella-Prevotella_pectinovora
Petrimonas_mucosa|ING2_E5A	GCF_900095795.1	Bacteria-Bacteroidetes-Bacteroidia-Bacteroidales-Dysgonomonadaceae-Petrimonas-Petrimonas_mucosa

		Bacteroides_neonati|MGYG_HGUT_01461	GCF_902375225.1	Bacteria-Bacteroidetes-Bacteroidia-Bacteroidales-Bacteroidaceae-Bacteroides-Bacteroides_neonati
		Prevotella_timonensis|DNF00076	GCF_002894165.1	Bacteria-Bacteroidetes-Bacteroidia-Bacteroidales-Prevotellaceae-Prevotella-Prevotella_timonensis
		Paludibacter_propionicigenes|WB4	GCF_000183135.1	Bacteria-Bacteroidetes-Bacteroidia-Bacteroidales-Paludibacteraceae-Paludibacter-Paludibacter_propionicigenes
		Prevotella_lacticifex|R5067	GCF_019973475.1	Bacteria-Bacteroidetes-Bacteroidia-Bacteroidales-Prevotellaceae-Prevotella-Prevotella_lacticifex
		Tenuifilum_thalassicum|38H_str	GCF_013265555.1	Bacteria-Bacteroidetes-Bacteroidia-Bacteroidales-Tenuifilaceae-Tenuifilum-Tenuifilum_thalassicum
		Heminiphilus_faecis|AM35	GCF_008728965.1	Bacteria-Bacteroidetes-Bacteroidia-Bacteroidales-Muribaculaceae-Heminiphilus-Heminiphilus_faecis
		Prevotella_micans|F0438	GCF_000243035.1	Bacteria-Bacteroidetes-Bacteroidia-Bacteroidales-Prevotellaceae-Prevotella-Prevotella_micans
		Prevotella_bergensis|DSM17361	GCF_000160535.1	Bacteria-Bacteroidetes-Bacteroidia-Bacteroidales-Prevotellaceae-Prevotella-Prevotella_bergensis
**COYNEBACTERIALES**	56	Corynebacterium_bovis|4826	GCF_003932295.1	Bacteria-Actinobacteria-Actinomycetia-Corynebacteriales-Corynebacteriaceae-Corynebacterium-Corynebacterium_bovis
		Mycolicibacterium_mageritense|JCM12375	GCF_010727475.1	Bacteria-Actinobacteria-Actinomycetia-Corynebacteriales-Mycobacteriaceae-Mycolicibacterium-Mycolicibacterium_mageritense
		Jongsikchunia_kroppenstedtii|NP8_5	GCF_000380485.1	Bacteria-Actinobacteria-Actinomycetia-Corynebacteriales-Gordoniaceae-Jongsikchunia-Jongsikchunia_kroppenstedtii
		Mycolicibacterium_monacense|JCM15658	GCF_010731575.1	Bacteria-Actinobacteria-Actinomycetia-Corynebacteriales-Mycobacteriaceae-Mycolicibacterium-Mycolicibacterium_monacense
		Corynebacterium_freneyi|DSM44506	GCF_017876455.1	Bacteria-Actinobacteria-Actinomycetia-Corynebacteriales-Corynebacteriaceae-Corynebacterium-Corynebacterium_freneyi
		Corynebacterium_cystitidis|NCTC11863	GCF_900187295.1	Bacteria-Actinobacteria-Actinomycetia-Corynebacteriales-Corynebacteriaceae-Corynebacterium-Corynebacterium_cystitidis
		Segniliparus_rotundus|DSM44985	GCF_000092825.1	Bacteria-Actinobacteria-Actinomycetia-Corynebacteriales-Segniliparaceae-Segniliparus-Segniliparus_rotundus
		Hoyosella_rhizosphaerae|CGMCC1.15478	GCF_014643175.1	Bacteria-Actinobacteria-Actinomycetia-Corynebacteriales-Mycobacteriaceae-Hoyosella-Hoyosella_rhizosphaerae
		Mycobacterium_marseillense|FLAC0026	GCF_002285715.1	Bacteria-Actinobacteria-Actinomycetia-Corynebacteriales-Mycobacteriaceae-Mycobacterium-Mycobacterium_marseillense
		Mycolicibacterium_phocaicum|JCM15301	GCF_010731115.1	Bacteria-Actinobacteria-Actinomycetia-Corynebacteriales-Mycobacteriaceae-Mycolicibacterium-Mycolicibacterium_phocaicum
		Corynebacterium_diphtheriae|ISS3319	GCF_002843135.1	Bacteria-Actinobacteria-Actinomycetia-Corynebacteriales-Corynebacteriaceae-Corynebacterium-Corynebacterium_diphtheriae
		Millisia_brevis|NBRC105863	GCF_001552615.1	Bacteria-Actinobacteria-Actinomycetia-Corynebacteriales-Gordoniaceae-Millisia-Millisia_brevis
		Williamsia_sterculiae|CPCC203464	GCF_900156495.1	Bacteria-Actinobacteria-Actinomycetia-Corynebacteriales-Nocardiaceae-Williamsia-Williamsia_sterculiae
		Corynebacterium_pyruviciproducens|ATCCBAA_1742	GCF_000411375.1	Bacteria-Actinobacteria-Actinomycetia-Corynebacteriales-Corynebacteriaceae-Corynebacterium-Corynebacterium_pyruviciproducens
		Dietzia_natronolimnaea|S_XJ_1	GCF_002289575.1	Bacteria-Actinobacteria-Actinomycetia-Corynebacteriales-Dietziaceae-Dietzia-Dietzia_natronolimnaea
		Mycobacterium_grossiae|DSM104744	GCF_008329645.1	Bacteria-Actinobacteria-Actinomycetia-Corynebacteriales-Mycobacteriaceae-Mycobacterium-Mycobacterium_grossiae
		Smaragdicoccus_niigatensis|DSM44881	GCF_000380645.1	Bacteria-Actinobacteria-Actinomycetia-Corynebacteriales-Nocardiaceae-Smaragdicoccus-Smaragdicoccus_niigatensis
		Corynebacterium_casei|LMGS_19264	GCF_000550785.1	Bacteria-Actinobacteria-Actinomycetia-Corynebacteriales-Corynebacteriaceae-Corynebacterium-Corynebacterium_casei
		Rhodococcus_spongiicola|LHW50502	GCF_004011835.1	Bacteria-Actinobacteria-Actinomycetia-Corynebacteriales-Nocardiaceae-Rhodococcus-Rhodococcus_spongiicola

	Corynebacterium_capitovis|DSM44611	GCF_000372085.1	Bacteria-Actinobacteria-Actinomycetia-Corynebacteriales-Corynebacteriaceae-Corynebacterium-Corynebacterium_capitovis
	Nocardia_pseudobrasiliensis|DSM44290	GCF_003350585.1	Bacteria-Actinobacteria-Actinomycetia-Corynebacteriales-Nocardiaceae-Nocardia-Nocardia_pseudobrasiliensis
	Rhodococcus_trifolii|CCM7905	GCF_014635345.1	Bacteria-Actinobacteria-Actinomycetia-Corynebacteriales-Nocardiaceae-Rhodococcus-Rhodococcus_trifolii
	Antrihabitans_stalactiti|YC2_7	GCF_012932915.1	Bacteria-Actinobacteria-Actinomycetia-Corynebacteriales-Nocardiaceae-Antrihabitans-Antrihabitans_stalactiti
	Corynebacterium_uberis|18M0132	GCF_020616335.1	Bacteria-Actinobacteria-Actinomycetia-Corynebacteriales-Corynebacteriaceae-Corynebacterium-Corynebacterium_uberis
	Corynebacterium_vitaeruminis|DSM20294	GCF_000550805.1	Bacteria-Actinobacteria-Actinomycetia-Corynebacteriales-Corynebacteriaceae-Corynebacterium-Corynebacterium_vitaeruminis
	Corynebacterium_pacaense|Marseille_P2417	GCF_900169525.1	Bacteria-Actinobacteria-Actinomycetia-Corynebacteriales-Corynebacteriaceae-Corynebacterium-Corynebacterium_pacaense
	Tomitella_biformata|AHU1821	GCF_000524475.1	Bacteria-Actinobacteria-Actinomycetia-Corynebacteriales-no_family-Tomitella-Tomitella_biformata
	Hoyosella_altamirensis|NBRC109631	GCF_001653095.1	Bacteria-Actinobacteria-Actinomycetia-Corynebacteriales-Mycobacteriaceae-Hoyosella-Hoyosella_altamirensis
	Corynebacterium_aurimucosum|DSM44827+ATCC700975	GCF_000022905.1	Bacteria-Actinobacteria-Actinomycetia-Corynebacteriales-Corynebacteriaceae-Corynebacterium-Corynebacterium_aurimucosum
	Corynebacterium_frankenforstense|ST18	GCF_001941485.1	Bacteria-Actinobacteria-Actinomycetia-Corynebacteriales-Corynebacteriaceae-Corynebacterium-Corynebacterium_frankenforstense
	Nocardia_bovistercoris|NEAU_351	GCF_015674855.1	Bacteria-Actinobacteria-Actinomycetia-Corynebacteriales-Nocardiaceae-Nocardia-Nocardia_bovistercoris
	Mycobacterium_bourgelatii|JCM30725	GCF_010723575.1	Bacteria-Actinobacteria-Actinomycetia-Corynebacteriales-Mycobacteriaceae-Mycobacterium-Mycobacterium_bourgelatii
	Corynebacterium_doosanense|CAU212T	GCF_000767055.1	Bacteria-Actinobacteria-Actinomycetia-Corynebacteriales-Corynebacteriaceae-Corynebacterium-Corynebacterium_doosanense
	Rhodococcus_marinonascens|NBRC14363	GCF_001894885.1	Bacteria-Actinobacteria-Actinomycetia-Corynebacteriales-Nocardiaceae-Rhodococcus-Rhodococcus_marinonascens
	Mycolicibacterium_septicum|PDNC012	GCF_016919345.1	Bacteria-Actinobacteria-Actinomycetia-Corynebacteriales-Mycobacteriaceae-Mycolicibacterium-Mycolicibacterium_septicum
	Gordonia_sihwensis|NBRC108236	GCF_000333035.1	Bacteria-Actinobacteria-Actinomycetia-Corynebacteriales-Gordoniaceae-Gordonia-Gordonia_sihwensis
	Corynebacterium_spheniscorum|J11+PG39	GCF_900113445.1	Bacteria-Actinobacteria-Actinomycetia-Corynebacteriales-Corynebacteriaceae-Corynebacterium-Corynebacterium_spheniscorum
	Corynebacterium_uropygiale|JCM32435	GCF_021568315.1	Bacteria-Actinobacteria-Actinomycetia-Corynebacteriales-Corynebacteriaceae-Corynebacterium-Corynebacterium_uropygiale
	Corynebacterium_gerontici|W8	GCF_003813985.1	Bacteria-Actinobacteria-Actinomycetia-Corynebacteriales-Corynebacteriaceae-Corynebacterium-Corynebacterium_gerontici
	Corynebacterium_glaucum|DSM30827	GCF_002287505.1	Bacteria-Actinobacteria-Actinomycetia-Corynebacteriales-Corynebacteriaceae-Corynebacterium-Corynebacterium_glaucum
	Mycobacterium_rufum|JCM16372	GCF_022374875.1	Bacteria-Actinobacteria-Actinomycetia-Corynebacteriales-Mycobacteriaceae-Mycobacterium-Mycobacterium_rufum
	Corynebacterium_renale|NCTC7448	GCF_900478035.1	Bacteria-Actinobacteria-Actinomycetia-Corynebacteriales-Corynebacteriaceae-Corynebacterium-Corynebacterium_renale
	Corynebacterium_mustelae|DSM45274	GCF_001020985.1	Bacteria-Actinobacteria-Actinomycetia-Corynebacteriales-Corynebacteriaceae-Corynebacterium-Corynebacterium_mustelae
	Aldersonia_kunmingensis|DSM45001	GCF_001646865.1	Bacteria-Actinobacteria-Actinomycetia-Corynebacteriales-Nocardiaceae-Aldersonia-Aldersonia_kunmingensis
	Mycolicibacterium_houstonense|typestrainATCC49403	GCF_900078665.2	Bacteria-Actinobacteria-Actinomycetia-Corynebacteriales-Mycobacteriaceae-Mycolicibacterium-Mycolicibacterium_houstonense
	Nocardia_higoensis|NBRC100133	GCF_000308595.1	Bacteria-Actinobacteria-Actinomycetia-Corynebacteriales-Nocardiaceae-Nocardia-Nocardia_higoensis

Rhodococcus_ruber|C1	GCF_016804345.1	Bacteria-Actinobacteria-Actinomycetia-Corynebacteriales-Nocardiaceae-Rhodococcus-Rhodococcus_ruber
Williamsia_marianensis|DSM44944	GCF_003096955.1	Bacteria-Actinobacteria-Actinomycetia-Corynebacteriales-Nocardiaceae-Williamsia-Williamsia_marianensis
Mycobacterium_koreense|JCM19956	GCF_010731835.1	Bacteria-Actinobacteria-Actinomycetia-Corynebacteriales-Mycobacteriaceae-Mycobacterium-Mycobacterium_koreense
Rhodococcus_kroppenstedtii|DSM44908	GCF_900111805.1	Bacteria-Actinobacteria-Actinomycetia-Corynebacteriales-Nocardiaceae-Rhodococcus-Rhodococcus_kroppenstedtii
Corynebacterium_massiliense|DSM45435	GCF_000420605.1	Bacteria-Actinobacteria-Actinomycetia-Corynebacteriales-Corynebacteriaceae-Corynebacterium-Corynebacterium_massiliense
Corynebacterium_sphenisci|DSM44792	GCF_001941505.1	Bacteria-Actinobacteria-Actinomycetia-Corynebacteriales-Corynebacteriaceae-Corynebacterium-Corynebacterium_sphenisci
Rhodococcus_coprophilus|NCTC10994	GCF_900478115.1	Bacteria-Actinobacteria-Actinomycetia-Corynebacteriales-Nocardiaceae-Rhodococcus-Rhodococcus_coprophilus
Nocardia_uniformis|NBRC13702	GCF_001613345.1	Bacteria-Actinobacteria-Actinomycetia-Corynebacteriales-Nocardiaceae-Nocardia-Nocardia_uniformis
Tsukamurella_tyrosinosolvens|PDNC001	GCF_016919465.1	Bacteria-Actinobacteria-Actinomycetia-Corynebacteriales-Tsukamurellaceae-Tsukamurella-Tsukamurella_tyrosinosolvens
Tsukamurella_paurometabola|DSM20162	GCF_000092225.1	Bacteria-Actinobacteria-Actinomycetia-Corynebacteriales-Tsukamurellaceae-Tsukamurella-Tsukamurella_paurometabola
Lawsonella_clevelandensis|X1036	GCF_001293125.1	Bacteria-Actinobacteria-Actinomycetia-Corynebacteriales-Lawsonellaceae-Lawsonella-Lawsonella_clevelandensis
Corynebacterium_argentoratense|DSM44202	GCF_000590555.1	Bacteria-Actinobacteria-Actinomycetia-Corynebacteriales-Corynebacteriaceae-Corynebacterium-Corynebacterium_argentoratense
Mycobacteroides_abscessus|GZ002	GCF_004028015.1	Bacteria-Actinobacteria-Actinomycetia-Corynebacteriales-Mycobacteriaceae-Mycobacteroides-Mycobacteroides_abscessus
Gordonia_iterans|Co17	GCF_002993285.1	Bacteria-Actinobacteria-Actinomycetia-Corynebacteriales-Gordoniaceae-Gordonia-Gordonia_iterans
Mycolicibacterium_gadium|JCM12688	GCF_010728925.1	Bacteria-Actinobacteria-Actinomycetia-Corynebacteriales-Mycobacteriaceae-Mycolicibacterium-Mycolicibacterium_gadium
Dietzia_timorensis|ID05_A0528	GCF_001659785.1	Bacteria-Actinobacteria-Actinomycetia-Corynebacteriales-Dietziaceae-Dietzia-Dietzia_timorensis
Gordonia_aichiensis|NBRC108223	GCF_000332975.1	Bacteria-Actinobacteria-Actinomycetia-Corynebacteriales-Gordoniaceae-Gordonia-Gordonia_aichiensis
Dietzia_cinnamea|NBRC102147	GCF_001571065.1	Bacteria-Actinobacteria-Actinomycetia-Corynebacteriales-Dietziaceae-Dietzia-Dietzia_cinnamea
Nocardia_aurantia|RB56	GCF_009604425.1	Bacteria-Actinobacteria-Actinomycetia-Corynebacteriales-Nocardiaceae-Nocardia-Nocardia_aurantia
Gordonia_amicalis|CEGA1	GCF_022059885.1	Bacteria-Actinobacteria-Actinomycetia-Corynebacteriales-Gordoniaceae-Gordonia-Gordonia_amicalis
Mycolicibacterium_rutilum|DSM45405	GCF_900108565.1	Bacteria-Actinobacteria-Actinomycetia-Corynebacteriales-Mycobacteriaceae-Mycolicibacterium-Mycolicibacterium_rutilum
Gordonia_hankookensis|ON_33	GCF_014673215.1	Bacteria-Actinobacteria-Actinomycetia-Corynebacteriales-Gordoniaceae-Gordonia-Gordonia_hankookensis
Mycobacterium_innocens|MK13	GCF_900566055.1	Bacteria-Actinobacteria-Actinomycetia-Corynebacteriales-Mycobacteriaceae-Mycobacterium-Mycobacterium_innocens
Corynebacterium_cyclohexanicum|ATCC51369	GCF_020886775.1	Bacteria-Actinobacteria-Actinomycetia-Corynebacteriales-Corynebacteriaceae-Corynebacterium-Corynebacterium_cyclohexanicum
Mycobacterium_shimoidei|P7336	GCF_900417275.1	Bacteria-Actinobacteria-Actinomycetia-Corynebacteriales-Mycobacteriaceae-Mycobacterium-Mycobacterium_shimoidei
Mycobacterium_lepromatosis|FJ924	GCF_000975265.2	Bacteria-Actinobacteria-Actinomycetia-Corynebacteriales-Mycobacteriaceae-Mycobacterium-Mycobacterium_lepromatosis
Corynebacterium_propinquum|FDAARGOS_1112	GCF_016728665.1	Bacteria-Actinobacteria-Actinomycetia-Corynebacteriales-Corynebacteriaceae-Corynebacterium-Corynebacterium_propinquum

	Mycolicibacterium_thermoresistibile|NCTC10409	GCF_900187065.1	Bacteria-Actinobacteria-Actinomycetia-Corynebacteriales-Mycobacteriaceae-Mycolicibacterium-Mycolicibacterium_thermoresistibile
	Mycolicibacterium_brumae|CIP1034565	GCF_002553575.1	Bacteria-Actinobacteria-Actinomycetia-Corynebacteriales-Mycobacteriaceae-Mycolicibacterium-Mycolicibacterium_brumae
	Gordonia_asplenii|TBRC11910	GCF_012933505.1	Bacteria-Actinobacteria-Actinomycetia-Corynebacteriales-Gordoniaceae-Gordonia-Gordonia_asplenii
	Rhodococcus_fascians|D188	GCF_001620305.1	Bacteria-Actinobacteria-Actinomycetia-Corynebacteriales-Nocardiaceae-Rhodococcus-Rhodococcus_fascians
	Rhodococcus_maanshanensis|NBRC100610	GCF_001894865.1	Bacteria-Actinobacteria-Actinomycetia-Corynebacteriales-Nocardiaceae-Rhodococcus-Rhodococcus_maanshanensis
	Mycobacterium_palauense|CECT8779	GCF_002592005.1	Bacteria-Actinobacteria-Actinomycetia-Corynebacteriales-Mycobacteriaceae-Mycobacterium-Mycobacterium_palauense
	Mycobacteroides_chelonae|CCUG47445	GCF_001632805.1	Bacteria-Actinobacteria-Actinomycetia-Corynebacteriales-Mycobacteriaceae-Mycobacteroides-Mycobacteroides_chelonae
	Mycobacterium_heraklionense|Davo	GCF_001021505.1	Bacteria-Actinobacteria-Actinomycetia-Corynebacteriales-Mycobacteriaceae-Mycobacterium-Mycobacterium_heraklionense
	Corynebacterium_urogenitale|LMM_1652	GCF_009026825.1	Bacteria-Actinobacteria-Actinomycetia-Corynebacteriales-Corynebacteriaceae-Corynebacterium-Corynebacterium_urogenitale
	Corynebacterium_glutamicum|SCgG2	GCF_000404185.1	Bacteria-Actinobacteria-Actinomycetia-Corynebacteriales-Corynebacteriaceae-Corynebacterium-Corynebacterium_glutamicum
	Corynebacterium_variabile|NBRC15286	GCF_006539825.1	Bacteria-Actinobacteria-Actinomycetia-Corynebacteriales-Corynebacteriaceae-Corynebacterium-Corynebacterium_variabile
	Williamsia_limnetica|DSM45521	GCF_003217475.1	Bacteria-Actinobacteria-Actinomycetia-Corynebacteriales-Nocardiaceae-Williamsia-Williamsia_limnetica
	Gordonia_crocea|NBRC107697	GCF_009932435.1	Bacteria-Actinobacteria-Actinomycetia-Corynebacteriales-Gordoniaceae-Gordonia-Gordonia_crocea
	Corynebacterium_uterequi|DSM45634	GCF_001021065.1	Bacteria-Actinobacteria-Actinomycetia-Corynebacteriales-Corynebacteriaceae-Corynebacterium-Corynebacterium_uterequi
	Segniliparus_rugosus|ATCCBAA_974	GCF_000185725.2	Bacteria-Actinobacteria-Actinomycetia-Corynebacteriales-Segniliparaceae-Segniliparus-Segniliparus_rugosus
	Mycobacterium_bohemicum|DSM44277	GCF_002102025.1	Bacteria-Actinobacteria-Actinomycetia-Corynebacteriales-Mycobacteriaceae-Mycobacterium-Mycobacterium_bohemicum
	Mycolicibacterium_anyangense|JCM30275	GCF_010731855.1	Bacteria-Actinobacteria-Actinomycetia-Corynebacteriales-Mycobacteriaceae-Mycolicibacterium-Mycolicibacterium_anyangense
	Mycobacterium_leprae|MRHRU_235_G	GCF_003253775.1	Bacteria-Actinobacteria-Actinomycetia-Corynebacteriales-Mycobacteriaceae-Mycobacterium-Mycobacterium_leprae
	Williamsia_herbipolensis|ARP1	GCF_000964005.1	Bacteria-Actinobacteria-Actinomycetia-Corynebacteriales-Nocardiaceae-Williamsia-Williamsia_herbipolensis
	Tomitella_gaofuii|HY172	GCF_014126825.1	Bacteria-Actinobacteria-Actinomycetia-Corynebacteriales-no_family-Tomitella-Tomitella_gaofuii
	Corynebacterium_qintianiae|MC1420	GCF_011038645.2	Bacteria-Actinobacteria-Actinomycetia-Corynebacteriales-Corynebacteriaceae-Corynebacterium-Corynebacterium_qintianiae
	Gordonia_soli|NBRC108243	GCF_000334455.1	Bacteria-Actinobacteria-Actinomycetia-Corynebacteriales-Gordoniaceae-Gordonia-Gordonia_soli
	Fodinicola_acaciae|GKU173	GCF_010993745.1	Bacteria-Actinobacteria-Actinomycetia-Corynebacteriales-no_family-Fodinicola-Fodinicola_acaciae
	Skermania_piniformis|DSM43998	GCF_019285775.1	Bacteria-Actinobacteria-Actinomycetia-Corynebacteriales-Gordoniaceae-Skermania-Skermania_piniformis
	Corynebacterium_durum|F0235	GCF_000318135.1	Bacteria-Actinobacteria-Actinomycetia-Corynebacteriales-Corynebacteriaceae-Corynebacterium-Corynebacterium_durum
	Tsukamurella_conjunctivitidis|HKU72	GCF_007858475.1	Bacteria-Actinobacteria-Actinomycetia-Corynebacteriales-Tsukamurellaceae-Tsukamurella-Tsukamurella_conjunctivitidis
	Gordonia_araii|NBRC100433	GCF_000241265.1	Bacteria-Actinobacteria-Actinomycetia-Corynebacteriales-Gordoniaceae-Gordonia-Gordonia_araii

**ENTEROBACTERALES**	105	Budvicia_diplopodorum|D9	GCF_009800925.1	Bacteria-Proteobacteria-Gammaproteobacteria-Enterobacterales-Budviciaceae-Budvicia-Budvicia_diplopodorum
		Limnobaculum_parvum|HYN0051	GCF_003096015.2	Bacteria-Proteobacteria-Gammaproteobacteria-Enterobacterales-Budviciaceae-Limnobaculum-Limnobaculum_parvum
		Siccibacter_turicensis|	GCF_004168465.1	Bacteria-Proteobacteria-Gammaproteobacteria-Enterobacterales-Enterobacteriaceae-Siccibacter-Siccibacter_turicensis
		Moranella_endobia|PCVAL	GCF_000364725.1	Bacteria-Proteobacteria-Gammaproteobacteria-Enterobacterales-Enterobacteriaceae-cMoranella-Moranella_endobia
		Raoultella_terrigena|JH01	GCF_012029655.1	Bacteria-Proteobacteria-Gammaproteobacteria-Enterobacterales-Enterobacteriaceae-Raoultella-Raoultella_terrigena
		Profftia_tarda|PROFFT	GCF_904848675.1	Bacteria-Proteobacteria-Gammaproteobacteria-Enterobacterales-Enterobacteriaceae-cProfftia-Profftia_tarda
		Leclercia_pneumoniae|49125	GCF_017348915.1	Bacteria-Proteobacteria-Gammaproteobacteria-Enterobacterales-Enterobacteriaceae-Leclercia-Leclercia_pneumoniae
		Mikella_endobia|MEPMAR	GCF_900048045.1	Bacteria-Proteobacteria-Gammaproteobacteria-Enterobacterales-Enterobacteriaceae-cMikella-Mikella_endobia
		Enterobacter_huaxiensis|090008	GCF_003594935.2	Bacteria-Proteobacteria-Gammaproteobacteria-Enterobacterales-Enterobacteriaceae-Enterobacter-Enterobacter_huaxiensis
		Pantoea_cypripedii|WS12375	GCF_017875755.1	Bacteria-Proteobacteria-Gammaproteobacteria-Enterobacterales-Erwiniaceae-Pantoea-Pantoea_cypripedii
		Edwardsiella_piscicida|18EpOKYJ	GCF_021733145.1	Bacteria-Proteobacteria-Gammaproteobacteria-Enterobacterales-Hafniaceae-Edwardsiella-Edwardsiella_piscicida
		Edaphovirga_cremea|DSM105170	GCF_003332275.1	Bacteria-Proteobacteria-Gammaproteobacteria-Enterobacterales-Enterobacteriaceae-Edaphovirga-Edaphovirga_cremea
		Kosakonia_cowanii|FBS223	GCF_004089895.1	Bacteria-Proteobacteria-Gammaproteobacteria-Enterobacterales-Enterobacteriaceae-Kosakonia-Kosakonia_cowanii
		Salmonella_enterica_subsp._enterica|LT2	GCF_000006945.2	Bacteria-Proteobacteria-Gammaproteobacteria-Enterobacterales-Enterobacteriaceae-Salmonella-Salmonella_enterica_subsp._enterica
		Arsenophonus_nasoniae|FIN	GCF_004768525.1	Bacteria-Proteobacteria-Gammaproteobacteria-Enterobacterales-Morganellaceae-Arsenophonus-Arsenophonus_nasoniae
		Hafnia_psychrotolerans|CGMCC1.12806	GCF_014639435.1	Bacteria-Proteobacteria-Gammaproteobacteria-Enterobacterales-Hafniaceae-Hafnia-Hafnia_psychrotolerans
		Pectobacterium_atrosepticum|21A	GCF_000740965.1	Bacteria-Proteobacteria-Gammaproteobacteria-Enterobacterales-Pectobacteriaceae-Pectobacterium-Pectobacterium_atrosepticum
		Pectobacterium_betavasculorum|NCPPB2795	GCF_000749845.1	Bacteria-Proteobacteria-Gammaproteobacteria-Enterobacterales-Pectobacteriaceae-Pectobacterium-Pectobacterium_betavasculorum
		Rosenbergiella_australiborealis|CdVSA20.1	GCF_018494035.1	Bacteria-Proteobacteria-Gammaproteobacteria-Enterobacterales-Erwiniaceae-Rosenbergiella-Rosenbergiella_australiborealis
		Lelliottia_amnigena|FDAARGOS_1445	GCF_019355955.1	Bacteria-Proteobacteria-Gammaproteobacteria-Enterobacterales-Enterobacteriaceae-Lelliottia-Lelliottia_amnigena
		Thorsellia_anophelis|DSM18579	GCF_900111395.1	Bacteria-Proteobacteria-Gammaproteobacteria-Enterobacterales-Thorselliaceae-Thorsellia-Thorsellia_anophelis
		Kluyvera_georgiana|WCH1410	GCF_001682915.1	Bacteria-Proteobacteria-Gammaproteobacteria-Enterobacterales-Enterobacteriaceae-Kluyvera-Kluyvera_georgiana
		Erwinia_haradaeae|ErCicurvipes	GCF_900698925.1	Bacteria-Proteobacteria-Gammaproteobacteria-Enterobacterales-Erwiniaceae-Erwinia-Erwinia_haradaeae
		Dickeya_parazeae|Ech586	GCF_000025065.1	Bacteria-Proteobacteria-Gammaproteobacteria-Enterobacterales-Pectobacteriaceae-Dickeya-Dickeya_parazeae
		Cronobacter_condimenti|LMG26250	GCF_001277255.1	Bacteria-Proteobacteria-Gammaproteobacteria-Enterobacterales-Enterobacteriaceae-Cronobacter-Cronobacter_condimenti
		Acerihabitans_arboris|SAP_6	GCF_010131535.1	Bacteria-Proteobacteria-Gammaproteobacteria-Enterobacterales-Pectobacteriaceae-Acerihabitans-Acerihabitans_arboris
		Enterobacteriaceae_sp.|Hcub_S	GCF_000287355.1	Bacteria-Proteobacteria-Gammaproteobacteria-Enterobacterales-Enterobacteriaceae-no_genus-Enterobacteriaceae_sp.

	Fukatsuia_symbiotica|5D	GCF_003122425.1	Bacteria-Proteobacteria-Gammaproteobacteria-Enterobacterales-Yersiniaceae-cFukatsuia-Fukatsuia_symbiotica
	Hamiltonella_defensa|T5A	GCF_000021705.1	Bacteria-Proteobacteria-Gammaproteobacteria-Enterobacterales-Enterobacteriaceae-cHamiltonella-Hamiltonella_defensa
	cBlochmannia_sp.|675	GCF_000973505.1	Bacteria-Proteobacteria-Gammaproteobacteria-Enterobacterales-Enterobacteriaceae-cBlochmannia-cBlochmannia_sp.
	Biostraticola_tofi|DSM19580	GCF_004343195.1	Bacteria-Proteobacteria-Gammaproteobacteria-Enterobacterales-Bruguierivoracaceae-Biostraticola-Biostraticola_tofi
	Mixta_theicola|QC88_366	GCF_002895925.1	Bacteria-Proteobacteria-Gammaproteobacteria-Enterobacterales-Erwiniaceae-Mixta-Mixta_theicola
	Serratia_microhaemolytica|ZS_11	GCF_004011885.1	Bacteria-Proteobacteria-Gammaproteobacteria-Enterobacterales-Yersiniaceae-Serratia-Serratia_microhaemolytica
	Arsenophonus_lipoptenae|CB	GCF_001534665.1	Bacteria-Proteobacteria-Gammaproteobacteria-Enterobacterales-Morganellaceae-Arsenophonus-Arsenophonus_lipoptenae
	Serratia_symbiotica|24.1	GCF_009831665.3	Bacteria-Proteobacteria-Gammaproteobacteria-Enterobacterales-Yersiniaceae-Serratia-Serratia_symbiotica
	Wigglesworthia_glossinidia|WGM	GCF_000247565.1	Bacteria-Proteobacteria-Gammaproteobacteria-Enterobacterales-Erwiniaceae-Wigglesworthia-Wigglesworthia_glossinidia
	Doolittlea_endobia|DEMHIR	GCF_900039485.1	Bacteria-Proteobacteria-Gammaproteobacteria-Enterobacterales-Enterobacteriaceae-cDoolittlea-Doolittlea_endobia
	cBlochmannia_sp.|CNIEBac	GCF_009827135.1	Bacteria-Proteobacteria-Gammaproteobacteria-Enterobacterales-Enterobacteriaceae-cBlochmannia-cBlochmannia_sp.
	Chania_multitudinisentens|RB_25	GCF_000520015.2	Bacteria-Proteobacteria-Gammaproteobacteria-Enterobacterales-Yersiniaceae-Chania-Chania_multitudinisentens
	Riesia_pediculicola|USDA	GCF_000093065.1	Bacteria-Proteobacteria-Gammaproteobacteria-Enterobacterales-Enterobacteriaceae-cRiesia-Riesia_pediculicola
	Obesumbacterium_proteus|LE8	GCF_000980985.1	Bacteria-Proteobacteria-Gammaproteobacteria-Enterobacterales-Hafniaceae-Obesumbacterium-Obesumbacterium_proteus
	Pseudocitrobacter_faecalis|CCM8478	GCF_014653055.1	Bacteria-Proteobacteria-Gammaproteobacteria-Enterobacterales-Enterobacteriaceae-Pseudocitrobacter-Pseudocitrobacter_faecalis
	Photorhabdus_tasmaniensis|T327	GCF_011189505.1	Bacteria-Proteobacteria-Gammaproteobacteria-Enterobacterales-Morganellaceae-Photorhabdus-Photorhabdus_tasmaniensis
	Mixta_mediterraneensis|Marseille_Q2057	GCF_014946725.1	Bacteria-Proteobacteria-Gammaproteobacteria-Enterobacterales-Erwiniaceae-Mixta-Mixta_mediterraneensis
	Providencia_burhodogranariea|DSM19968	GCF_000314855.2	Bacteria-Proteobacteria-Gammaproteobacteria-Enterobacterales-Morganellaceae-Providencia-Providencia_burhodogranariea
	Tatumella_saanichensis|NML06_3099	GCF_000439375.1	Bacteria-Proteobacteria-Gammaproteobacteria-Enterobacterales-Erwiniaceae-Tatumella-Tatumella_saanichensis
	Sodalis_sp.|HBA	GCF_900161835.1	Bacteria-Proteobacteria-Gammaproteobacteria-Enterobacterales-Bruguierivoracaceae-Sodalis-Sodalis_sp.
	Kalamiella_piersonii|IIIF1SW_P2	GCF_003612015.1	Bacteria-Proteobacteria-Gammaproteobacteria-Enterobacterales-Erwiniaceae-Kalamiella-Kalamiella_piersonii
	Erwinia_beijingensis|JZB2120001	GCF_022647505.1	Bacteria-Proteobacteria-Gammaproteobacteria-Enterobacterales-Erwiniaceae-Erwinia-Erwinia_beijingensis
	Riesia_pediculischaeffi|PTSK	GCF_002073895.1	Bacteria-Proteobacteria-Gammaproteobacteria-Enterobacterales-Enterobacteriaceae-cRiesia-Riesia_pediculischaeffi
	Rahnella_ecdela|FRB231	GCF_019049625.1	Bacteria-Proteobacteria-Gammaproteobacteria-Enterobacterales-Yersiniaceae-Rahnella-Rahnella_ecdela
	Buchnera_aphidicola|BpBaizongiapistaciae	GCF_000007725.1	Bacteria-Proteobacteria-Gammaproteobacteria-Enterobacterales-Erwiniaceae-Buchnera-Buchnera_aphidicola
	Lonsdalea_britannica|477	GCF_003515985.1	Bacteria-Proteobacteria-Gammaproteobacteria-Enterobacterales-Pectobacteriaceae-Lonsdalea-Lonsdalea_britannica
	Gullanella_endobia|GEFVIR	GCF_900048035.1	Bacteria-Proteobacteria-Gammaproteobacteria-Enterobacterales-Enterobacteriaceae-cGullanella-Gullanella_endobia

	Franconibacter_helveticus|CS_23	GCF_003207695.1	Bacteria-Proteobacteria-Gammaproteobacteria-Enterobacterales-Enterobacteriaceae-Franconibacter-Franconibacter_helveticus
	Pragia_fontium|NCTC12284	GCF_900638655.1	Bacteria-Proteobacteria-Gammaproteobacteria-Enterobacterales-Budviciaceae-Pragia-Pragia_fontium
	Sodalis_glossinidius|morsitans	GCF_000010085.1	Bacteria-Proteobacteria-Gammaproteobacteria-Enterobacterales-Bruguierivoracaceae-Sodalis-Sodalis_glossinidius
	Moellerella_wisconsensis|W65	GCF_022592395.1	Bacteria-Proteobacteria-Gammaproteobacteria-Enterobacterales-Morganellaceae-Moellerella-Moellerella_wisconsensis
	Phytobacter_massiliensis|MGYG_HGUT_01426	GCF_902374985.1	Bacteria-Proteobacteria-Gammaproteobacteria-Enterobacterales-Enterobacteriaceae-Phytobacter-Phytobacter_massiliensis
	Brenneria_goodwinii|FRB141	GCF_002291445.1	Bacteria-Proteobacteria-Gammaproteobacteria-Enterobacterales-Pectobacteriaceae-Brenneria-Brenneria_goodwinii
	Escherichia_coli|K_12substr.MG1655	GCF_000005845.2	Bacteria-Proteobacteria-Gammaproteobacteria-Enterobacterales-Enterobacteriaceae-Escherichia-Escherichia_coli
	Pluralibacter_gergoviae|FDAARGOS_186	GCF_003019925.1	Bacteria-Proteobacteria-Gammaproteobacteria-Enterobacterales-Enterobacteriaceae-Pluralibacter-Pluralibacter_gergoviae
	Superficieibacter_electus|BP_1	GCF_002915575.1	Bacteria-Proteobacteria-Gammaproteobacteria-Enterobacterales-Enterobacteriaceae-Superficieibacter-Superficieibacter_electus
	Morganella_morganii|MGYG_HGUT_02512	GCF_902387845.1	Bacteria-Proteobacteria-Gammaproteobacteria-Enterobacterales-Morganellaceae-Morganella-Morganella_morganii
	Rouxiella_silvae|213	GCF_002093625.1	Bacteria-Proteobacteria-Gammaproteobacteria-Enterobacterales-Yersiniaceae-Rouxiella-Rouxiella_silvae
	Citrobacter_murliniae|P080CCL	GCF_004801125.1	Bacteria-Proteobacteria-Gammaproteobacteria-Enterobacterales-Enterobacteriaceae-Citrobacter-Citrobacter_murliniae
	Rahnella_laticis|SAP_17	GCF_015644585.1	Bacteria-Proteobacteria-Gammaproteobacteria-Enterobacterales-Yersiniaceae-Rahnella-Rahnella_laticis
	Samsonia_erythrinae|DSM16730	GCF_004342665.1	Bacteria-Proteobacteria-Gammaproteobacteria-Enterobacterales-Pectobacteriaceae-Samsonia-Samsonia_erythrinae
	Buttiauxella_warmboldiae|CCUG35512	GCF_003818135.1	Bacteria-Proteobacteria-Gammaproteobacteria-Enterobacterales-Enterobacteriaceae-Buttiauxella-Buttiauxella_warmboldiae
	Yersinia_pestis|A1122	GCF_000222975.1	Bacteria-Proteobacteria-Gammaproteobacteria-Enterobacterales-Yersiniaceae-Yersinia-Yersinia_pestis
	Leminorella_grimontii|ATCC33999	GCF_000735425.1	Bacteria-Proteobacteria-Gammaproteobacteria-Enterobacterales-Budviciaceae-Leminorella-Leminorella_grimontii
	Regiella_insecticola|Tut	GCF_013373955.1	Bacteria-Proteobacteria-Gammaproteobacteria-Enterobacterales-Enterobacteriaceae-cRegiella-Regiella_insecticola
	Pantoea_edessiphila|SoEO	GCF_002933345.1	Bacteria-Proteobacteria-Gammaproteobacteria-Enterobacterales-Erwiniaceae-Pantoea-Pantoea_edessiphila
	Enterobacteriaceae_sp.|TRABTM	GCF_900090215.1	Bacteria-Proteobacteria-Gammaproteobacteria-Enterobacterales-Enterobacteriaceae-no_genus-Enterobacteriaceae_sp.
	Izhakiella_capsodis|N6PO6	GCF_900115045.1	Bacteria-Proteobacteria-Gammaproteobacteria-Enterobacterales-Erwiniaceae-Izhakiella-Izhakiella_capsodis
	Enterobacteriaceae_sp.|Ceuc_S	GCF_000287335.1	Bacteria-Proteobacteria-Gammaproteobacteria-Enterobacterales-Enterobacteriaceae-no_genus-Enterobacteriaceae_sp.
	Mangrovibacter_phragmitis|MP23	GCF_001655675.1	Bacteria-Proteobacteria-Gammaproteobacteria-Enterobacterales-Enterobacteriaceae-Mangrovibacter-Mangrovibacter_phragmitis
	Annandia_adelgestsuga|ENA	GCF_003956045.1	Bacteria-Proteobacteria-Gammaproteobacteria-Enterobacterales-Enterobacteriaceae-cAnnandia-Annandia_adelgestsuga
	Yokenella_regensburgei|DSM5079	GCF_003634235.1	Bacteria-Proteobacteria-Gammaproteobacteria-Enterobacterales-Enterobacteriaceae-Yokenella-Yokenella_regensburgei
	Hoaglandella_endobia|HETPER	GCF_900044015.1	Bacteria-Proteobacteria-Gammaproteobacteria-Enterobacterales-Enterobacteriaceae-cHoaglandella-Hoaglandella_endobia
	Plesiomonas_shigelloides|7A	GCF_020991025.1	Bacteria-Proteobacteria-Gammaproteobacteria-Enterobacterales-Enterobacteriaceae-Plesiomonas-Plesiomonas_shigelloides

		Entomohabitans_teleogrylli|SCU_B244	GCF_001484765.1	Bacteria-Proteobacteria-Gammaproteobacteria-Enterobacterales-Enterobacteriaceae-Entomohabitans-Entomohabitans_teleogrylli
		cBlochmannia_sp.|757	GCF_000973545.1	Bacteria-Proteobacteria-Gammaproteobacteria-Enterobacterales-Enterobacteriaceae-cBlochmannia-cBlochmannia_sp.
		Pantoea_carbekii|US	GCF_000971765.1	Bacteria-Proteobacteria-Gammaproteobacteria-Enterobacterales-Erwiniaceae-Pantoea-Pantoea_carbekii
		Ishikawaella_capsulata|Mpkobe	GCF_000828515.1	Bacteria-Proteobacteria-Gammaproteobacteria-Enterobacterales-Enterobacteriaceae-cIshikawaella-Ishikawaella_capsulata
		Musicola_paradisiaca|Ech703	GCF_000023545.1	Bacteria-Proteobacteria-Gammaproteobacteria-Enterobacterales-Pectobacteriaceae-Musicola-Musicola_paradisiaca
		Citrobacter_koseri|ATCCBAA_895	GCF_000018045.1	Bacteria-Proteobacteria-Gammaproteobacteria-Enterobacterales-Enterobacteriaceae-Citrobacter-Citrobacter_koseri
		Atlantibacter_subterranea|ZYL01	GCF_007570865.1	Bacteria-Proteobacteria-Gammaproteobacteria-Enterobacterales-Enterobacteriaceae-Atlantibacter-Atlantibacter_subterranea
		Phytobacter_diazotrophicus|UAEU22	GCF_012923785.1	Bacteria-Proteobacteria-Gammaproteobacteria-Enterobacterales-Enterobacteriaceae-Phytobacter-Phytobacter_diazotrophicus
		Schneideria_nysicola|NR	GCF_019923565.1	Bacteria-Proteobacteria-Gammaproteobacteria-Enterobacterales-Enterobacteriaceae-cSchneideria-Schneideria_nysicola
		Profftia_lariciata|Ad13_081	GCF_020541265.1	Bacteria-Proteobacteria-Gammaproteobacteria-Enterobacterales-Enterobacteriaceae-cProfftia-Profftia_lariciata
		Shimwellia_blattae|DSM4481	GCF_000262305.1	Bacteria-Proteobacteria-Gammaproteobacteria-Enterobacterales-Enterobacteriaceae-Shimwellia-Shimwellia_blattae
		Nissabacter_archeti|JGM97	GCF_018257325.1	Bacteria-Proteobacteria-Gammaproteobacteria-Enterobacterales-Yersiniaceae-Nissabacter-Nissabacter_archeti
		Xenorhabdus_poinarii|G6	GCF_000968175.1	Bacteria-Proteobacteria-Gammaproteobacteria-Enterobacterales-Morganellaceae-Xenorhabdus-Xenorhabdus_poinarii
		Klebsiella_quasipneumoniae|KqPF26	GCF_016415705.1	Bacteria-Proteobacteria-Gammaproteobacteria-Enterobacterales-Enterobacteriaceae-Klebsiella-Klebsiella_quasipneumoniae
		Affinibrenneria_salicis|L3_3HA	GCF_008710095.1	Bacteria-Proteobacteria-Gammaproteobacteria-Enterobacterales-Pectobacteriaceae-Affinibrenneria-Affinibrenneria_salicis
		Cedecea_davisae|DSM4568	GCF_000412335.2	Bacteria-Proteobacteria-Gammaproteobacteria-Enterobacterales-Enterobacteriaceae-Cedecea-Cedecea_davisae
		Proteus_alimentorum|08MAS0041	GCF_002607735.1	Bacteria-Proteobacteria-Gammaproteobacteria-Enterobacterales-Morganellaceae-Proteus-Proteus_alimentorum
		Hafnia_paralvei|AVS0177	GCF_020150375.1	Bacteria-Proteobacteria-Gammaproteobacteria-Enterobacterales-Hafniaceae-Hafnia-Hafnia_paralvei
		Jinshanibacter_allomyrinae|BWR_B9	GCF_016649425.1	Bacteria-Proteobacteria-Gammaproteobacteria-Enterobacterales-Budviciaceae-Jinshanibacter-Jinshanibacter_allomyrinae
**ESCHERICHIA**	85	Escherichia_fergusonii|FDAARGOS_1499	GCF_020097475.1	Bacteria-Proteobacteria-Gammaproteobacteria-Enterobacterales-Enterobacteriaceae-Escherichia-Escherichia_fergusonii
		Escherichia_coli|ATCC11775	GCF_003697165.2	Bacteria-Proteobacteria-Gammaproteobacteria-Enterobacterales-Enterobacteriaceae-Escherichia-Escherichia_coli
		Shigella_dysenteriae|BCW_4872	GCF_001932995.2	Bacteria-Proteobacteria-Gammaproteobacteria-Enterobacterales-Enterobacteriaceae-Shigella-Shigella_dysenteriae
		Shigella_sonnei|ATCC29930	GCF_002950395.1	Bacteria-Proteobacteria-Gammaproteobacteria-Enterobacterales-Enterobacteriaceae-Shigella-Shigella_sonnei
		Escherichia_whittamii|C2_3	GCF_020283705.1	Bacteria-Proteobacteria-Gammaproteobacteria-Enterobacterales-Enterobacteriaceae-Escherichia-Escherichia_whittamii
		Escherichia_marmotae|HT073016	GCF_000807695.1	Bacteria-Proteobacteria-Gammaproteobacteria-Enterobacterales-Enterobacteriaceae-Escherichia-Escherichia_marmotae
		Shigella_dysenteriae|NCTC4837	GCF_900457215.1	Bacteria-Proteobacteria-Gammaproteobacteria-Enterobacterales-Enterobacteriaceae-Shigella-Shigella_dysenteriae
		Escherichia_coli|DSM30083	GCF_024519395.1	Bacteria-Proteobacteria-Gammaproteobacteria-Enterobacterales-Enterobacteriaceae-Escherichia-Escherichia_coli

		Escherichia_albertii|DSM17582	GCF_022833075.1	Bacteria-Proteobacteria-Gammaproteobacteria-Enterobacterales-Enterobacteriaceae-Escherichia-Escherichia_albertii
		Escherichia_coli|K_12substr.MG1655	GCF_000005845.2	Bacteria-Proteobacteria-Gammaproteobacteria-Enterobacterales-Enterobacteriaceae-Escherichia-Escherichia_coli
		Escherichia_marmotae|H1_003_0086_C_F	GCF_902709585.1	Bacteria-Proteobacteria-Gammaproteobacteria-Enterobacterales-Enterobacteriaceae-Escherichia-Escherichia_marmotae
		Shigella_boydii|600384	GCF_001905915.1	Bacteria-Proteobacteria-Gammaproteobacteria-Enterobacterales-Enterobacteriaceae-Shigella-Shigella_boydii
		Escherichia_albertii|NBRC107761	GCF_000759775.1	Bacteria-Proteobacteria-Gammaproteobacteria-Enterobacterales-Enterobacteriaceae-Escherichia-Escherichia_albertii
		Escherichia_fergusonii|RHB19_C05	GCF_013892435.1	Bacteria-Proteobacteria-Gammaproteobacteria-Enterobacterales-Enterobacteriaceae-Escherichia-Escherichia_fergusonii
		Shigella_flexneri|301	GCF_000006925.2	Bacteria-Proteobacteria-Gammaproteobacteria-Enterobacterales-Enterobacteriaceae-Shigella-Shigella_flexneri
		Escherichia_coli|Sakaisubstr.RIMD0509952	GCF_000008865.2	Bacteria-Proteobacteria-Gammaproteobacteria-Enterobacterales-Enterobacteriaceae-Escherichia-Escherichia_coli
		Shigella_dysenteriae|ATCC13313	GCF_002949675.1	Bacteria-Proteobacteria-Gammaproteobacteria-Enterobacterales-Enterobacteriaceae-Shigella-Shigella_dysenteriae
		Escherichia_fergusonii|ATCC35469T	GCF_000026225.1	Bacteria-Proteobacteria-Gammaproteobacteria-Enterobacterales-Enterobacteriaceae-Escherichia-Escherichia_fergusonii
		Escherichia_albertii|Sample167	GCF_016904755.1	Bacteria-Proteobacteria-Gammaproteobacteria-Enterobacterales-Enterobacteriaceae-Escherichia-Escherichia_albertii
		Escherichia_marmotae|HT073016	GCF_002900365.1	Bacteria-Proteobacteria-Gammaproteobacteria-Enterobacterales-Enterobacteriaceae-Escherichia-Escherichia_marmotae
		Escherichia_whittamii|Sa2BVA5	GCF_014836715.1	Bacteria-Proteobacteria-Gammaproteobacteria-Enterobacterales-Enterobacteriaceae-Escherichia-Escherichia_whittamii
		Shigella_boydii|NCTC12985	GCF_900457095.1	Bacteria-Proteobacteria-Gammaproteobacteria-Enterobacterales-Enterobacteriaceae-Shigella-Shigella_boydii
		Shigella_flexneri|ATCC29903	GCF_002950215.1	Bacteria-Proteobacteria-Gammaproteobacteria-Enterobacterales-Enterobacteriaceae-Shigella-Shigella_flexneri
		Shigella_boydii|DMBSH130	GCF_003572535.1	Bacteria-Proteobacteria-Gammaproteobacteria-Enterobacterales-Enterobacteriaceae-Shigella-Shigella_boydii
		Shigella_sonnei|SE6_1	GCF_013374815.1	Bacteria-Proteobacteria-Gammaproteobacteria-Enterobacterales-Enterobacteriaceae-Shigella-Shigella_sonnei
		Shigella_boydii|FDAARGOS_1139	GCF_016726285.1	Bacteria-Proteobacteria-Gammaproteobacteria-Enterobacterales-Enterobacteriaceae-Shigella-Shigella_boydii
**HYPHOMICROBIALES**	21	Gellertiella_hungarica|DSM29853	GCF_014197145.1	Bacteria-Proteobacteria-Alphaproteobacteria-Hyphomicrobiales-Rhizobiaceae-Gellertiella-Gellertiella_hungarica
		Oceaniradius_stylonematis|StC1	GCF_003149475.2	Bacteria-Proteobacteria-Alphaproteobacteria-Hyphomicrobiales-Phyllobacteriaceae-Oceaniradius-Oceaniradius_stylonematis
		Aquamicrobium_defluvii|DSM11603	GCF_004363725.1	Bacteria-Proteobacteria-Alphaproteobacteria-Hyphomicrobiales-Phyllobacteriaceae-Aquamicrobium-Aquamicrobium_defluvii
		Pinisolibacter_aquiterrae|MA2_2	GCF_020859625.1	Bacteria-Proteobacteria-Alphaproteobacteria-Hyphomicrobiales-Ancalomicrobiaceae-Pinisolibacter-Pinisolibacter_aquiterrae
		Agrobacterium_tumefaciens|12D1	GCF_003667905.1	Bacteria-Proteobacteria-Alphaproteobacteria-Hyphomicrobiales-Rhizobiaceae-Agrobacterium-Agrobacterium_tumefaciens
		Roseibium_litorale|4C16A	GCF_014842915.1	Bacteria-Proteobacteria-Alphaproteobacteria-Hyphomicrobiales-Stappiaceae-Roseibium-Roseibium_litorale
		Oharaeibacter_diazotrophicus|DSM102969	GCF_004362745.1	Bacteria-Proteobacteria-Alphaproteobacteria-Hyphomicrobiales-Pleomorphomonadaceae-Oharaeibacter-Oharaeibacter_diazotrophicus
		Tepidamorphus_gemmatus|DSM19345	GCF_004346195.1	Bacteria-Proteobacteria-Alphaproteobacteria-Hyphomicrobiales-Tepidamorphaceae-Tepidamorphus-Tepidamorphus_gemmatus
		Methylopila_capsulata|DSM6130	GCF_016907975.1	Bacteria-Proteobacteria-Alphaproteobacteria-Hyphomicrobiales-Methylocystaceae-Methylopila-Methylopila_capsulata

Dichotomicrobium_thermohalophilum|DSM5002	GCF_003550175.1	Bacteria-Proteobacteria-Alphaproteobacteria-Hyphomicrobiales-Hyphomicrobiaceae-Dichotomicrobium-Dichotomicrobium_thermohalophilum
Martelella_alba|BGMRC2036	GCF_006516955.1	Bacteria-Proteobacteria-Alphaproteobacteria-Hyphomicrobiales-Aurantimonadaceae-Martelella-Martelella_alba
Consotaella_salsifontis|USBA369	GCF_900167365.1	Bacteria-Proteobacteria-Alphaproteobacteria-Hyphomicrobiales-Aurantimonadaceae-Consotaella-Consotaella_salsifontis
Aureimonas_leprariae|YIM132180	GCF_008802405.1	Bacteria-Proteobacteria-Alphaproteobacteria-Hyphomicrobiales-Aurantimonadaceae-Aureimonas-Aureimonas_leprariae
Methylobacterium_oryzihabitans|TER_1	GCF_004004555.2	Bacteria-Proteobacteria-Alphaproteobacteria-Hyphomicrobiales-Methylobacteriaceae-Methylobacterium-Methylobacterium_oryzihabitans
Phreatobacter_cathodiphilus|S_12	GCF_003008515.1	Bacteria-Proteobacteria-Alphaproteobacteria-Hyphomicrobiales-Phreatobacteraceae-Phreatobacter-Phreatobacter_cathodiphilus
Hyphomicrobium_facile|DSM1565	GCF_900116175.1	Bacteria-Proteobacteria-Alphaproteobacteria-Hyphomicrobiales-Hyphomicrobiaceae-Hyphomicrobium-Hyphomicrobium_facile
Aquamicrobium_aerolatum|DSM21857	GCF_900113935.1	Bacteria-Proteobacteria-Alphaproteobacteria-Hyphomicrobiales-Phyllobacteriaceae-Aquamicrobium-Aquamicrobium_aerolatum
Enterovirga_rhinocerotis|DSM25903	GCF_004363955.1	Bacteria-Proteobacteria-Alphaproteobacteria-Hyphomicrobiales-Methylobacteriaceae-Enterovirga-Enterovirga_rhinocerotis
Filomicrobium_insigne|CGMCC1.6497	GCF_900104305.1	Bacteria-Proteobacteria-Alphaproteobacteria-Hyphomicrobiales-Hyphomicrobiaceae-Filomicrobium-Filomicrobium_insigne
Oricola_thermophila|MEBiC13590	GCF_013358405.1	Bacteria-Proteobacteria-Alphaproteobacteria-Hyphomicrobiales-Phyllobacteriaceae-Oricola-Oricola_thermophila
Beijerinckia_indica_subsp._indica|ATCC9039	GCF_000019845.1	Bacteria-Proteobacteria-Alphaproteobacteria-Hyphomicrobiales-Beijerinckiaceae-Beijerinckia-Beijerinckia_indica_subsp._indica
Prosthecomicrobium_pneumaticum|DSM16268	GCF_014199915.1	Bacteria-Proteobacteria-Alphaproteobacteria-Hyphomicrobiales-Hyphomicrobiaceae-Prosthecomicrobium-Prosthecomicrobium_pneumaticum
Microvirga_massiliensis|JC119	GCF_001006805.1	Bacteria-Proteobacteria-Alphaproteobacteria-Hyphomicrobiales-Methylobacteriaceae-Microvirga-Microvirga_massiliensis
Rhodobium_orientis|DSM11290	GCF_014197785.1	Bacteria-Proteobacteria-Alphaproteobacteria-Hyphomicrobiales-Rhodobiaceae-Rhodobium-Rhodobium_orientis
Pelagibacterium_montanilacus|CCL18	GCF_003992665.1	Bacteria-Proteobacteria-Alphaproteobacteria-Hyphomicrobiales-Devosiaceae-Pelagibacterium-Pelagibacterium_montanilacus
Tepidicaulis_marinus|MA2	GCF_000739695.1	Bacteria-Proteobacteria-Alphaproteobacteria-Hyphomicrobiales-Parvibaculaceae-Tepidicaulis-Tepidicaulis_marinus
Allorhizobium_undicola|ATCC700741	GCF_000621665.1	Bacteria-Proteobacteria-Alphaproteobacteria-Hyphomicrobiales-Rhizobiaceae-Allorhizobium-Allorhizobium_undicola
Fodinicurvata_sediminis|DSM21159	GCF_000420625.1	Bacteria-Proteobacteria-Alphaproteobacteria-Hyphomicrobiales-Rhodovibrionaceae-Fodinicurvata-Fodinicurvata_sediminis
Labrys_okinawensis|RP1T	GCF_002982075.1	Bacteria-Proteobacteria-Alphaproteobacteria-Hyphomicrobiales-Xanthobacteraceae-Labrys-Labrys_okinawensis
Aminobacter_aganoensis|DSM7051	GCF_014206975.1	Bacteria-Proteobacteria-Alphaproteobacteria-Hyphomicrobiales-Phyllobacteriaceae-Aminobacter-Aminobacter_aganoensis
Devosia_limi|DSM17137	GCF_900128975.1	Bacteria-Proteobacteria-Alphaproteobacteria-Hyphomicrobiales-Devosiaceae-Devosia-Devosia_limi
Limoniibacter_endophyticus|KCTC42097	GCF_014652835.1	Bacteria-Proteobacteria-Alphaproteobacteria-Hyphomicrobiales-Hyphomicrobiaceae-Limoniibacter-Limoniibacter_endophyticus
Rhodopseudomonas_palustris|RCB100	GCF_016584445.1	Bacteria-Proteobacteria-Alphaproteobacteria-Hyphomicrobiales-Nitrobacteraceae-Rhodopseudomonas-Rhodopseudomonas_palustris
Rhizobium_rosettiformans|W3	GCF_004912135.1	Bacteria-Proteobacteria-Alphaproteobacteria-Hyphomicrobiales-Rhizobiaceae-Rhizobium-Rhizobium_rosettiformans
Chelatococcus_composti|DSM101465	GCF_018398355.1	Bacteria-Proteobacteria-Alphaproteobacteria-Hyphomicrobiales-Chelatococcaceae-Chelatococcus-Chelatococcus_composti
Nitratireductor_aestuarii|CGMCC1.15320	GCF_014641695.1	Bacteria-Proteobacteria-Alphaproteobacteria-Hyphomicrobiales-Phyllobacteriaceae-Nitratireductor-Nitratireductor_aestuarii

Rhodovibrio_salinarum|DSM9154	GCF_000515255.1	Bacteria-Proteobacteria-Alphaproteobacteria-Hyphomicrobiales-Rhodovibrionaceae-Rhodovibrio-Rhodovibrio_salinarum
Hoeflea_olei|JC234	GCF_001703635.1	Bacteria-Proteobacteria-Alphaproteobacteria-Hyphomicrobiales-Phyllobacteriaceae-Hoeflea-Hoeflea_olei
Stappia_taiwanensis|CCM7757	GCF_014635285.1	Bacteria-Proteobacteria-Alphaproteobacteria-Hyphomicrobiales-Stappiaceae-Stappia-Stappia_taiwanensis
Nitratireductor_aquimarinus|TN35_13	GCF_017255295.1	Bacteria-Proteobacteria-Alphaproteobacteria-Hyphomicrobiales-Phyllobacteriaceae-Nitratireductor-Nitratireductor_aquimarinus
Limimonas_halophila|DSM25584	GCF_900100655.1	Bacteria-Proteobacteria-Alphaproteobacteria-Hyphomicrobiales-Rhodovibrionaceae-Limimonas-Limimonas_halophila
Methyloligella_halotolerans|VKMB_2706	GCF_001708935.1	Bacteria-Proteobacteria-Alphaproteobacteria-Hyphomicrobiales-Hyphomicrobiaceae-Methyloligella-Methyloligella_halotolerans
Methyloceanibacter_superfactus|R_67175	GCF_001723305.1	Bacteria-Proteobacteria-Alphaproteobacteria-Hyphomicrobiales-Hyphomicrobiaceae-Methyloceanibacter-Methyloceanibacter_superfactus
Alsobacter_soli|SH9	GCF_003004785.1	Bacteria-Proteobacteria-Alphaproteobacteria-Hyphomicrobiales-Alsobacteraceae-Alsobacter-Alsobacter_soli
Arsenicitalea_aurantiaca|42_50	GCF_003994485.1	Bacteria-Proteobacteria-Alphaproteobacteria-Hyphomicrobiales-Devosiaceae-Arsenicitalea-Arsenicitalea_aurantiaca
Hansschlegelia_zhihuaiae|S93	GCF_004103825.1	Bacteria-Proteobacteria-Alphaproteobacteria-Hyphomicrobiales-Methylocystaceae-Hansschlegelia-Hansschlegelia_zhihuaiae
Kaustia_mangrovi|R1DC25	GCF_015482775.1	Bacteria-Proteobacteria-Alphaproteobacteria-Hyphomicrobiales-Parvibaculaceae-Kaustia-Kaustia_mangrovi
Pararhizobium_mangrovi|BGMRC6574	GCF_006516965.1	Bacteria-Proteobacteria-Alphaproteobacteria-Hyphomicrobiales-Rhizobiaceae-Pararhizobium-Pararhizobium_mangrovi
Pleomorphomonas_koreensis|DSM23070	GCF_000425185.1	Bacteria-Proteobacteria-Alphaproteobacteria-Hyphomicrobiales-Pleomorphomonadaceae-Pleomorphomonas-Pleomorphomonas_koreensis
Parvibaculum_indicum|DSM25305	GCF_011762095.1	Bacteria-Proteobacteria-Alphaproteobacteria-Hyphomicrobiales-Parvibaculaceae-Parvibaculum-Parvibaculum_indicum
Limibacillus_halophilus|CECT8803	GCF_014191775.1	Bacteria-Proteobacteria-Alphaproteobacteria-Hyphomicrobiales-Rhodovibrionaceae-Limibacillus-Limibacillus_halophilus
Rhodoplanes_elegans|DSM11907	GCF_016653355.1	Bacteria-Proteobacteria-Alphaproteobacteria-Hyphomicrobiales-Hyphomicrobiaceae-Rhodoplanes-Rhodoplanes_elegans
Methylocystis_heyeri|H2	GCF_004802635.2	Bacteria-Proteobacteria-Alphaproteobacteria-Hyphomicrobiales-Methylocystaceae-Methylocystis-Methylocystis_heyeri
Propylenella_binzhouense|L72	GCF_009866965.1	Bacteria-Proteobacteria-Alphaproteobacteria-Hyphomicrobiales-Propylenellaceae-Propylenella-Propylenella_binzhouense
Zhengella_mangrovi|X9_2_2	GCF_002727065.1	Bacteria-Proteobacteria-Alphaproteobacteria-Hyphomicrobiales-Phyllobacteriaceae-Zhengella-Zhengella_mangrovi
Hongsoonwoonella_zoysiae|SY4_7	GCF_013266695.1	Bacteria-Proteobacteria-Alphaproteobacteria-Hyphomicrobiales-Stappiaceae-Hongsoonwoonella-Hongsoonwoonella_zoysiae
Pelagibius_marinus|NBU2595	GCF_014925385.1	Bacteria-Proteobacteria-Alphaproteobacteria-Hyphomicrobiales-Rhodovibrionaceae-Pelagibius-Pelagibius_marinus
Roseiarcus_fermentans|DSM24875	GCF_003315135.1	Bacteria-Proteobacteria-Alphaproteobacteria-Hyphomicrobiales-Roseiarcaceae-Roseiarcus-Roseiarcus_fermentans
Salinarimonas_soli|BN140002	GCF_008386575.1	Bacteria-Proteobacteria-Alphaproteobacteria-Hyphomicrobiales-Salinarimonadaceae-Salinarimonas-Salinarimonas_soli
Pseudovibrio_hongkongensis|UST20140214_015B	GCF_001561995.1	Bacteria-Proteobacteria-Alphaproteobacteria-Hyphomicrobiales-Stappiaceae-Pseudovibrio-Pseudovibrio_hongkongensis
Ancylobacter_polymorphus|ZM13	GCF_022836935.1	Bacteria-Proteobacteria-Alphaproteobacteria-Hyphomicrobiales-Xanthobacteraceae-Ancylobacter-Ancylobacter_polymorphus
Azorhizobium_doebereinerae|UFLA1_100	GCF_000473085.1	Bacteria-Proteobacteria-Alphaproteobacteria-Hyphomicrobiales-Xanthobacteraceae-Azorhizobium-Azorhizobium_doebereinerae
Acuticoccus_sediminis|PTG4_2	GCF_003258595.1	Bacteria-Proteobacteria-Alphaproteobacteria-Hyphomicrobiales-Amorphaceae-Acuticoccus-Acuticoccus_sediminis

Amorphus_coralli|DSM19760	GCF_000374525.1	Bacteria-Proteobacteria-Alphaproteobacteria-Hyphomicrobiales-Amorphaceae-Amorphus-Amorphus_coralli
Vineibacter_terrae|CC_CFT640	GCF_008039615.1	Bacteria-Proteobacteria-Alphaproteobacteria-Hyphomicrobiales-no_family-Vineibacter-Vineibacter_terrae
Methylovirgula_ligni|BW863	GCF_004135935.1	Bacteria-Proteobacteria-Alphaproteobacteria-Hyphomicrobiales-Beijerinckiaceae-Methylovirgula-Methylovirgula_ligni
Caenibius_tardaugens|NBRC16725	GCF_003860345.1	Bacteria-Proteobacteria-Alphaproteobacteria-Hyphomicrobiales-Hyphomicrobiaceae-Caenibius-Caenibius_tardaugens
Xanthobacter_tagetidis|DSM11105	GCF_014206845.1	Bacteria-Proteobacteria-Alphaproteobacteria-Hyphomicrobiales-Xanthobacteraceae-Xanthobacter-Xanthobacter_tagetidis
Terrihabitans_soli|IZ6	GCF_014191545.1	Bacteria-Proteobacteria-Alphaproteobacteria-Hyphomicrobiales-no_family-Terrihabitans-Terrihabitans_soli
Afifella_aestuarii|JA968	GCF_004023665.1	Bacteria-Proteobacteria-Alphaproteobacteria-Hyphomicrobiales-Afifellaceae-Afifella-Afifella_aestuarii
Phyllobacterium_sophorae|CCBAU03422	GCF_003010965.1	Bacteria-Proteobacteria-Alphaproteobacteria-Hyphomicrobiales-Phyllobacteriaceae-Phyllobacterium-Phyllobacterium_sophorae
Blastochloris_tepida|GI	GCF_003966715.1	Bacteria-Proteobacteria-Alphaproteobacteria-Hyphomicrobiales-Blastochloridaceae-Blastochloris-Blastochloris_tepida
Sinorhizobium_meliloti|2011	GCF_000346065.1	Bacteria-Proteobacteria-Alphaproteobacteria-Hyphomicrobiales-Rhizobiaceae-Sinorhizobium-Sinorhizobium_meliloti
Paenochrobactrum_gallinarii|DSM22336	GCF_014205685.1	Bacteria-Proteobacteria-Alphaproteobacteria-Hyphomicrobiales-Brucellaceae-Paenochrobactrum-Paenochrobactrum_gallinarii
Brucella_tritici|TA93	GCF_008932285.1	Bacteria-Proteobacteria-Alphaproteobacteria-Hyphomicrobiales-Brucellaceae-Brucella-Brucella_tritici
Pyruvatibacter_mobilis|CGMCC1.15125	GCF_014640905.1	Bacteria-Proteobacteria-Alphaproteobacteria-Hyphomicrobiales-Parvibaculaceae-Pyruvatibacter-Pyruvatibacter_mobilis
Aquabacter_cavernae|Sn_9_2	GCF_003993795.1	Bacteria-Proteobacteria-Alphaproteobacteria-Hyphomicrobiales-Hyphomicrobiaceae-Aquabacter-Aquabacter_cavernae
Bartonella_florencae|R4	GCF_000312525.1	Bacteria-Proteobacteria-Alphaproteobacteria-Hyphomicrobiales-Bartonellaceae-Bartonella-Bartonella_florencae
Shinella_fusca|DSM21319	GCF_014203155.1	Bacteria-Proteobacteria-Alphaproteobacteria-Hyphomicrobiales-Rhizobiaceae-Shinella-Shinella_fusca
Rhodoblastus_sphagnicola|DSM16996	GCF_002937075.1	Bacteria-Proteobacteria-Alphaproteobacteria-Hyphomicrobiales-Beijerinckiaceae-Rhodoblastus-Rhodoblastus_sphagnicola
Cucumibacter_marinus|DSM18995	GCF_000429865.1	Bacteria-Proteobacteria-Alphaproteobacteria-Hyphomicrobiales-Devosiaceae-Cucumibacter-Cucumibacter_marinus
Afipia_felis|NCTC12499	GCF_900445155.1	Bacteria-Proteobacteria-Alphaproteobacteria-Hyphomicrobiales-Nitrobacteraceae-Afipia-Afipia_felis
Hartmannibacter_diazotrophicus|E19T	GCF_900231165.1	Bacteria-Proteobacteria-Alphaproteobacteria-Hyphomicrobiales-Pleomorphomonadaceae-Hartmannibacter-Hartmannibacter_diazotrophicus
Rhodoligotrophos_defluvii|lm1	GCF_005281615.1	Bacteria-Proteobacteria-Alphaproteobacteria-Hyphomicrobiales-Parvibaculaceae-Rhodoligotrophos-Rhodoligotrophos_defluvii
Rhabdaerophilum_calidifontis|SYSUG02060	GCF_008641065.1	Bacteria-Proteobacteria-Alphaproteobacteria-Hyphomicrobiales-Rhabdaerophilaceae-Rhabdaerophilum-Rhabdaerophilum_calidifontis
Fulvimarina_manganoxydans|CGMCC1.10972	GCF_900176465.1	Bacteria-Proteobacteria-Alphaproteobacteria-Hyphomicrobiales-Aurantimonadaceae-Fulvimarina-Fulvimarina_manganoxydans
Liberibacter_crescens|BT_0	GCF_001543305.1	Bacteria-Proteobacteria-Alphaproteobacteria-Hyphomicrobiales-Rhizobiaceae-Liberibacter-Liberibacter_crescens
Cohaesibacter_intestini|YE_B6	GCF_003324485.1	Bacteria-Proteobacteria-Alphaproteobacteria-Hyphomicrobiales-Cohaesibacteraceae-Cohaesibacter-Cohaesibacter_intestini
Salinarimonas_ramus|CGMCC1.9161	GCF_014645695.1	Bacteria-Proteobacteria-Alphaproteobacteria-Hyphomicrobiales-Salinarimonadaceae-Salinarimonas-Salinarimonas_ramus
Liberibacter_solanacearum|CLso_ZC1	GCF_000183665.1	Bacteria-Proteobacteria-Alphaproteobacteria-Hyphomicrobiales-Rhizobiaceae-Liberibacter-Liberibacter_solanacearum

		Mesorhizobium_temperatum|SDW018	GCF_002284575.1	Bacteria-Proteobacteria-Alphaproteobacteria-Hyphomicrobiales-Phyllobacteriaceae-Mesorhizobium-Mesorhizobium_temperatum
		Pseudoxanthobacter_soli|DSM19599	GCF_900148505.1	Bacteria-Proteobacteria-Alphaproteobacteria-Hyphomicrobiales-Pseudoxanthobacteraceae-Pseudoxanthobacter-Pseudoxanthobacter_soli
		Rhodomicrobium_lacus|JA980	GCF_003992725.1	Bacteria-Proteobacteria-Alphaproteobacteria-Hyphomicrobiales-Hyphomicrobiaceae-Rhodomicrobium-Rhodomicrobium_lacus
		Bosea_robiniae|DSM26672	GCF_900102525.1	Bacteria-Proteobacteria-Alphaproteobacteria-Hyphomicrobiales-Boseaceae-Bosea-Bosea_robiniae
		Chthonobacter_rhizosphaerae|yh7_1	GCF_013839525.1	Bacteria-Proteobacteria-Alphaproteobacteria-Hyphomicrobiales-Pleomorphomonadaceae-Chthonobacter-Chthonobacter_rhizosphaerae
		Bradyrhizobium_erythrophlei|GAS1201	GCF_900142985.1	Bacteria-Proteobacteria-Alphaproteobacteria-Hyphomicrobiales-Nitrobacteraceae-Bradyrhizobium-Bradyrhizobium_erythrophlei
		Lutibaculum_baratangense|AMV1	GCF_000496075.1	Bacteria-Proteobacteria-Alphaproteobacteria-Hyphomicrobiales-Tepidamorphaceae-Lutibaculum-Lutibaculum_baratangense
		Aestuariivirga_litoralis|KCTC52945	GCF_003234965.1	Bacteria-Proteobacteria-Alphaproteobacteria-Hyphomicrobiales-Aestuariivirgaceae-Aestuariivirga-Aestuariivirga_litoralis
		Tistlia_consotensis|DSM21585	GCF_900188055.1	Bacteria-Proteobacteria-Alphaproteobacteria-Hyphomicrobiales-Rhodovibrionaceae-Tistlia-Tistlia_consotensis
		Reyranella_soli|NBRC108950	GCF_007992495.1	Bacteria-Proteobacteria-Alphaproteobacteria-Hyphomicrobiales-Reyranellaceae-Reyranella-Reyranella_soli
**METHANOCOCCALES**	27	Methanothermococcus_okinawensis|IH1	GCF_000179575.2	Archaea-Euryarchaeota-Methanococci-Methanococcales-Methanococcaceae-Methanothermococcus-Methanothermococcus_okinawensis
		Methanocaldococcus_villosus|KIN24_T80	GCF_000371805.1	Archaea-Euryarchaeota-Methanococci-Methanococcales-Methanocaldococcaceae-Methanocaldococcus-Methanocaldococcus_villosus
		Methanotorris_igneus|Kol5	GCF_000214415.1	Archaea-Euryarchaeota-Methanococci-Methanococcales-Methanocaldococcaceae-Methanotorris-Methanotorris_igneus
		Methanocaldococcus_vulcanius|M7	GCF_000024625.1	Archaea-Euryarchaeota-Methanococci-Methanococcales-Methanocaldococcaceae-Methanocaldococcus-Methanocaldococcus_vulcanius
		Methanococcus_vannielii|SB	GCF_000017165.1	Archaea-Euryarchaeota-Methanococci-Methanococcales-Methanococcaceae-Methanococcus-Methanococcus_vannielii
		Methanotorris_formicicus|Mc_S_70	GCF_000243455.1	Archaea-Euryarchaeota-Methanococci-Methanococcales-Methanocaldococcaceae-Methanotorris-Methanotorris_formicicus
		Methanococcus_voltae|C4	GCF_017875565.1	Archaea-Euryarchaeota-Methanococci-Methanococcales-Methanococcaceae-Methanococcus-Methanococcus_voltae
		Methanocaldococcus_villosus|KIN24_T80	GCF_000363885.1	Archaea-Euryarchaeota-Methanococci-Methanococcales-Methanocaldococcaceae-Methanocaldococcus-Methanocaldococcus_villosus
		Methanocaldococcus_fervens|AG86	GCF_000023985.1	Archaea-Euryarchaeota-Methanococci-Methanococcales-Methanocaldococcaceae-Methanocaldococcus-Methanocaldococcus_fervens
		Methanocaldococcus_jannaschii|DSM2661	GCF_000091665.1	Archaea-Euryarchaeota-Methanococci-Methanococcales-Methanocaldococcaceae-Methanocaldococcus-Methanocaldococcus_jannaschii
		Methanocaldococcus_bathoardescens|JH146	GCF_000739065.1	Archaea-Euryarchaeota-Methanococci-Methanococcales-Methanocaldococcaceae-Methanocaldococcus-Methanocaldococcus_bathoardescens
		Methanococcus_aeolicus|Nankai_3	GCF_000017185.1	Archaea-Euryarchaeota-Methanococci-Methanococcales-Methanococcaceae-Methanococcus-Methanococcus_aeolicus
		Methanococcus_maripaludis|DSM2067	GCF_002945325.1	Archaea-Euryarchaeota-Methanococci-Methanococcales-Methanococcaceae-Methanococcus-Methanococcus_maripaludis
		Methanothermococcus_thermolithotrophicus|DSM2095	GCF_000376965.1	Archaea-Euryarchaeota-Methanococci-Methanococcales-Methanococcaceae-Methanothermococcus-Methanothermococcus_thermolithotrophicus
		Methanocaldococcus_infernus|ME	GCF_000092305.1	Archaea-Euryarchaeota-Methanococci-Methanococcales-Methanocaldococcaceae-Methanocaldococcus-Methanocaldococcus_infernus
		Methanofervidicoccus_abyssi|HHB	GCF_004310395.1	Archaea-Euryarchaeota-Methanococci-Methanococcales-no_family-Methanofervidicoccus-Methanofervidicoccus_abyssi
**PSEUDOMONADALES**	39	Pseudomonas_urmiensis|SWRI10	GCF_014268815.2	Bacteria-Proteobacteria-Gammaproteobacteria-Pseudomonadales-Pseudomonadaceae-Pseudomonas-Pseudomonas_urmiensis

Marinobacter_daqiaonensis|YCSA40	GCF_012070355.1	Bacteria-Proteobacteria-Gammaproteobacteria-Pseudomonadales-Marinobacteraceae-Marinobacter-Marinobacter_daqiaonensis
Tamilnaduibacter_salinus|DSM28688	GCF_003096655.1	Bacteria-Proteobacteria-Gammaproteobacteria-Pseudomonadales-Marinobacteraceae-Tamilnaduibacter-Tamilnaduibacter_salinus
Pseudomonas_sihuiensis|KCTC32246	GCF_900106015.1	Bacteria-Proteobacteria-Gammaproteobacteria-Pseudomonadales-Pseudomonadaceae-Pseudomonas-Pseudomonas_sihuiensis
Permianibacter_fluminis|IMCC34836	GCF_013179735.1	Bacteria-Proteobacteria-Gammaproteobacteria-Pseudomonadales-Pseudomonadaceae-Permianibacter-Permianibacter_fluminis
Pseudomonas_duriflava|CGMCC1.6858	GCF_007830155.1	Bacteria-Proteobacteria-Gammaproteobacteria-Pseudomonadales-Pseudomonadaceae-Pseudomonas-Pseudomonas_duriflava
Denitrificimonas_caeni|DSM24390	GCF_000421765.1	Bacteria-Proteobacteria-Gammaproteobacteria-Pseudomonadales-Pseudomonadaceae-Denitrificimonas-Denitrificimonas_caeni
Marinobacter_litoralis|Sw_45	GCF_003336705.1	Bacteria-Proteobacteria-Gammaproteobacteria-Pseudomonadales-Marinobacteraceae-Marinobacter-Marinobacter_litoralis
Marinobacter_aromaticivorans|D15_8P	GCF_002806975.1	Bacteria-Proteobacteria-Gammaproteobacteria-Pseudomonadales-Marinobacteraceae-Marinobacter-Marinobacter_aromaticivorans
Azotobacter_vinelandii|DJ+ATCCBAA_1303	GCF_000021045.1	Bacteria-Proteobacteria-Gammaproteobacteria-Pseudomonadales-Pseudomonadaceae-Azotobacter-Azotobacter_vinelandii
Pseudomonas_pohangensis|DSM17875	GCF_900105995.1	Bacteria-Proteobacteria-Gammaproteobacteria-Pseudomonadales-Pseudomonadaceae-Pseudomonas-Pseudomonas_pohangensis
Marinobacter_pelagius|CGMCC1.6775	GCF_900114925.1	Bacteria-Proteobacteria-Gammaproteobacteria-Pseudomonadales-Marinobacteraceae-Marinobacter-Marinobacter_pelagius
Marinobacter_changyiensis|CLL7_20	GCF_009193265.1	Bacteria-Proteobacteria-Gammaproteobacteria-Pseudomonadales-Marinobacteraceae-Marinobacter-Marinobacter_changyiensis
Pseudomonas_matsuisoli|JCM30078	GCF_014647635.1	Bacteria-Proteobacteria-Gammaproteobacteria-Pseudomonadales-Pseudomonadaceae-Pseudomonas-Pseudomonas_matsuisoli
Marinobacter_shengliensis|SL013A34A2	GCF_003007715.1	Bacteria-Proteobacteria-Gammaproteobacteria-Pseudomonadales-Marinobacteraceae-Marinobacter-Marinobacter_shengliensis
Marinobacter_salicampi|ISL_40	GCF_011074795.1	Bacteria-Proteobacteria-Gammaproteobacteria-Pseudomonadales-Marinobacteraceae-Marinobacter-Marinobacter_salicampi
Marinobacter_caseinilyticus|M3_13	GCF_011074955.1	Bacteria-Proteobacteria-Gammaproteobacteria-Pseudomonadales-Marinobacteraceae-Marinobacter-Marinobacter_caseinilyticus
Thiopseudomonas_alkaliphila|D3318	GCF_001267255.1	Bacteria-Proteobacteria-Gammaproteobacteria-Pseudomonadales-Pseudomonadaceae-Thiopseudomonas-Thiopseudomonas_alkaliphila
Pseudomonas_tohonis|TUM18999	GCF_012767755.2	Bacteria-Proteobacteria-Gammaproteobacteria-Pseudomonadales-Pseudomonadaceae-Pseudomonas-Pseudomonas_tohonis
Pseudomonas_akapageensis|PS24	GCF_011355085.1	Bacteria-Proteobacteria-Gammaproteobacteria-Pseudomonadales-Pseudomonadaceae-Pseudomonas-Pseudomonas_akapageensis
Marinobacter_halodurans|YJ_S3_2	GCF_004327985.1	Bacteria-Proteobacteria-Gammaproteobacteria-Pseudomonadales-Marinobacteraceae-Marinobacter-Marinobacter_halodurans
Azotobacter_beijerinckii|DSM378	GCF_900110885.1	Bacteria-Proteobacteria-Gammaproteobacteria-Pseudomonadales-Pseudomonadaceae-Azotobacter-Azotobacter_beijerinckii
Perlucidibaca_piscinae|DSM21586	GCF_000420045.1	Bacteria-Proteobacteria-Gammaproteobacteria-Pseudomonadales-Perlucidibacaceae-Perlucidibaca-Perlucidibaca_piscinae
Marinobacter_antarcticus|CGMCC1.10835	GCF_900142385.1	Bacteria-Proteobacteria-Gammaproteobacteria-Pseudomonadales-Marinobacteraceae-Marinobacter-Marinobacter_antarcticus
Pseudomonas_schmalbachii|Milli4	GCF_017589465.1	Bacteria-Proteobacteria-Gammaproteobacteria-Pseudomonadales-Pseudomonadaceae-Pseudomonas-Pseudomonas_schmalbachii
Pseudomonas_guryensis|SR9	GCF_014164785.1	Bacteria-Proteobacteria-Gammaproteobacteria-Pseudomonadales-Pseudomonadaceae-Pseudomonas-Pseudomonas_guryensis
Pseudomonas_cavernae|K2W31S_8	GCF_003595175.1	Bacteria-Proteobacteria-Gammaproteobacteria-Pseudomonadales-Pseudomonadaceae-Pseudomonas-Pseudomonas_cavernae
Marinobacter_psychrophilus|20041	GCF_001043175.1	Bacteria-Proteobacteria-Gammaproteobacteria-Pseudomonadales-Marinobacteraceae-Marinobacter-Marinobacter_psychrophilus

Pseudomonas_syringae_group_genomosp._3|DC3000	GCF_000007805.1	Bacteria-Proteobacteria-Gammaproteobacteria-Pseudomonadales-Pseudomonadaceae-Pseudomonas-Pseudomonas_syringae_group_genomosp._3
Marinobacter_xestospongiae|JCM17469	GCF_023156385.1	Bacteria-Proteobacteria-Gammaproteobacteria-Pseudomonadales-Marinobacteraceae-Marinobacter-Marinobacter_xestospongiae
Pseudomonas_eucalypticola|NP_1	GCF_013374995.1	Bacteria-Proteobacteria-Gammaproteobacteria-Pseudomonadales-Pseudomonadaceae-Pseudomonas-Pseudomonas_eucalypticola
Pseudomonas_mangrovi|TC11	GCF_003052585.1	Bacteria-Proteobacteria-Gammaproteobacteria-Pseudomonadales-Pseudomonadaceae-Pseudomonas-Pseudomonas_mangrovi
Marinobacter_nanhaiticus|D15_8W	GCF_000364845.1	Bacteria-Proteobacteria-Gammaproteobacteria-Pseudomonadales-Marinobacteraceae-Marinobacter-Marinobacter_nanhaiticus
Pseudomonas_lalucatii|R1b54	GCF_018398425.1	Bacteria-Proteobacteria-Gammaproteobacteria-Pseudomonadales-Pseudomonadaceae-Pseudomonas-Pseudomonas_lalucatii
Atopomonas_hussainii|JCM19513	GCF_900109735.1	Bacteria-Proteobacteria-Gammaproteobacteria-Pseudomonadales-Pseudomonadaceae-Atopomonas-Atopomonas_hussainii
Perlucidibaca_aquatica|BK296	GCF_001676655.1	Bacteria-Proteobacteria-Gammaproteobacteria-Pseudomonadales-Perlucidibacaceae-Perlucidibaca-Perlucidibaca_aquatica
Halopseudomonas_salina|CGMCC1.12482	GCF_014637955.1	Bacteria-Proteobacteria-Gammaproteobacteria-Pseudomonadales-Pseudomonadaceae-Halopseudomonas-Halopseudomonas_salina
Pseudomonas_jilinensis|JS15_10A1	GCF_003586265.1	Bacteria-Proteobacteria-Gammaproteobacteria-Pseudomonadales-Pseudomonadaceae-Pseudomonas-Pseudomonas_jilinensis
Pseudomonas_mangiferae|DMKUBBB3_04	GCF_007109405.1	Bacteria-Proteobacteria-Gammaproteobacteria-Pseudomonadales-Pseudomonadaceae-Pseudomonas-Pseudomonas_mangiferae
Pseudomonas_borbori|DSM17834	GCF_900115555.1	Bacteria-Proteobacteria-Gammaproteobacteria-Pseudomonadales-Pseudomonadaceae-Pseudomonas-Pseudomonas_borbori
Azomonas_agilis|DSM375	GCF_007830255.1	Bacteria-Proteobacteria-Gammaproteobacteria-Pseudomonadales-Pseudomonadaceae-Azomonas-Azomonas_agilis
Pseudomonas_oryzae|KCTC32247	GCF_900104805.1	Bacteria-Proteobacteria-Gammaproteobacteria-Pseudomonadales-Pseudomonadaceae-Pseudomonas-Pseudomonas_oryzae
Pseudomonas_carbonaria|CIP111764	GCF_904061905.1	Bacteria-Proteobacteria-Gammaproteobacteria-Pseudomonadales-Pseudomonadaceae-Pseudomonas-Pseudomonas_carbonaria
Permianibacter_aggregans|HW001	GCF_009756665.1	Bacteria-Proteobacteria-Gammaproteobacteria-Pseudomonadales-Pseudomonadaceae-Permianibacter-Permianibacter_aggregans
Pseudomonas_flexibilis|JCM14085	GCF_000806415.1	Bacteria-Proteobacteria-Gammaproteobacteria-Pseudomonadales-Pseudomonadaceae-Pseudomonas-Pseudomonas_flexibilis
Pseudomonas_panipatensis|CCM7469	GCF_900099785.1	Bacteria-Proteobacteria-Gammaproteobacteria-Pseudomonadales-Pseudomonadaceae-Pseudomonas-Pseudomonas_panipatensis
Pseudomonas_cavernicola|K1S02_6	GCF_003596405.1	Bacteria-Proteobacteria-Gammaproteobacteria-Pseudomonadales-Pseudomonadaceae-Pseudomonas-Pseudomonas_cavernicola
Marinobacter_denitrificans|JB02H27	GCF_008795955.1	Bacteria-Proteobacteria-Gammaproteobacteria-Pseudomonadales-Marinobacteraceae-Marinobacter-Marinobacter_denitrificans
Pseudomonas_benzenivorans|DSM8628	GCF_900100495.1	Bacteria-Proteobacteria-Gammaproteobacteria-Pseudomonadales-Pseudomonadaceae-Pseudomonas-Pseudomonas_benzenivorans
Pseudomonas_azotifigens|DSM17556	GCF_000425625.1	Bacteria-Proteobacteria-Gammaproteobacteria-Pseudomonadales-Pseudomonadaceae-Pseudomonas-Pseudomonas_azotifigens
Marinobacter_zhanjiangensis|KCTC22280	GCF_014651935.1	Bacteria-Proteobacteria-Gammaproteobacteria-Pseudomonadales-Marinobacteraceae-Marinobacter-Marinobacter_zhanjiangensis
Pseudomonas_aeruginosa|PAO1	GCF_000006765.1	Bacteria-Proteobacteria-Gammaproteobacteria-Pseudomonadales-Pseudomonadaceae-Pseudomonas-Pseudomonas_aeruginosa
Pseudomonas_nosocomialis|A3170	GCF_005876855.1	Bacteria-Proteobacteria-Gammaproteobacteria-Pseudomonadales-Pseudomonadaceae-Pseudomonas-Pseudomonas_nosocomialis
Ventosimonas_gracilis|CV58	GCF_001580025.1	Bacteria-Proteobacteria-Gammaproteobacteria-Pseudomonadales-Ventosimonadaceae-Ventosimonas-Ventosimonas_gracilis
Marinobacter_halotolerans|NBRC110910	GCF_008795985.1	Bacteria-Proteobacteria-Gammaproteobacteria-Pseudomonadales-Marinobacteraceae-Marinobacter-Marinobacter_halotolerans

Pseudomonas_lalkuanensis|PE08	GCF_008807375.1	Bacteria-Proteobacteria-Gammaproteobacteria-Pseudomonadales-Pseudomonadaceae-Pseudomonas-Pseudomonas_lalkuanensis
Azomonas_macrocytogenes|CECT4462	GCF_014191985.1	Bacteria-Proteobacteria-Gammaproteobacteria-Pseudomonadales-Pseudomonadaceae-Azomonas-Azomonas_macrocytogenes
Pseudomonas_typographi|CA3A	GCF_014694385.1	Bacteria-Proteobacteria-Gammaproteobacteria-Pseudomonadales-Pseudomonadaceae-Pseudomonas-Pseudomonas_typographi
Marinobacter_subterrani|JG233	GCF_001045555.1	Bacteria-Proteobacteria-Gammaproteobacteria-Pseudomonadales-Marinobacteraceae-Marinobacter-Marinobacter_subterrani
Pseudomonas_quercus|hsmgli_8	GCF_012033695.1	Bacteria-Proteobacteria-Gammaproteobacteria-Pseudomonadales-Pseudomonadaceae-Pseudomonas-Pseudomonas_quercus
Marinobacter_piscensis|Abdou3	GCF_007671655.1	Bacteria-Proteobacteria-Gammaproteobacteria-Pseudomonadales-Marinobacteraceae-Marinobacter-Marinobacter_piscensis
Pseudomonas_kuykendallii|NRRLB_59562	GCF_900106975.1	Bacteria-Proteobacteria-Gammaproteobacteria-Pseudomonadales-Pseudomonadaceae-Pseudomonas-Pseudomonas_kuykendallii
Marinobacter_mobilis|CGMCC1.7059	GCF_900106945.1	Bacteria-Proteobacteria-Gammaproteobacteria-Pseudomonadales-Marinobacteraceae-Marinobacter-Marinobacter_mobilis
Pseudomonas_songnenensis|NEAU_ST5_5	GCF_003696315.1	Bacteria-Proteobacteria-Gammaproteobacteria-Pseudomonadales-Pseudomonadaceae-Pseudomonas-Pseudomonas_songnenensis
Halopseudomonas_aestusnigri|CECT8317	GCF_900108005.1	Bacteria-Proteobacteria-Gammaproteobacteria-Pseudomonadales-Pseudomonadaceae-Halopseudomonas-Halopseudomonas_aestusnigri
Mangrovitalea_sediminis|M11_4	GCF_002514725.1	Bacteria-Proteobacteria-Gammaproteobacteria-Pseudomonadales-Marinobacteraceae-Mangrovitalea-Mangrovitalea_sediminis
Halopseudomonas_pelagia|CL_AP6	GCF_000410875.1	Bacteria-Proteobacteria-Gammaproteobacteria-Pseudomonadales-Pseudomonadaceae-Halopseudomonas-Halopseudomonas_pelagia
Pseudomonas_brassicae|MAFF212427	GCF_010671725.1	Bacteria-Proteobacteria-Gammaproteobacteria-Pseudomonadales-Pseudomonadaceae-Pseudomonas-Pseudomonas_brassicae
Pseudomonas_entomophila|L48	GCF_000026105.1	Bacteria-Proteobacteria-Gammaproteobacteria-Pseudomonadales-Pseudomonadaceae-Pseudomonas-Pseudomonas_entomophila
Pseudomonas_indica|NBRC103045	GCF_002091635.1	Bacteria-Proteobacteria-Gammaproteobacteria-Pseudomonadales-Pseudomonadaceae-Pseudomonas-Pseudomonas_indica
Pseudomonas_profundi|M5	GCF_008638305.1	Bacteria-Proteobacteria-Gammaproteobacteria-Pseudomonadales-Pseudomonadaceae-Pseudomonas-Pseudomonas_profundi
Halopseudomonas_sabulinigri|JCM14963	GCF_900105255.1	Bacteria-Proteobacteria-Gammaproteobacteria-Pseudomonadales-Pseudomonadaceae-Halopseudomonas-Halopseudomonas_sabulinigri
Entomomonas_moraniae|QZS01	GCF_003991975.1	Bacteria-Proteobacteria-Gammaproteobacteria-Pseudomonadales-Pseudomonadaceae-Entomomonas-Entomomonas_moraniae
Halopseudomonas_pachastrellae|JCM12285	GCF_900114765.1	Bacteria-Proteobacteria-Gammaproteobacteria-Pseudomonadales-Pseudomonadaceae-Halopseudomonas-Halopseudomonas_pachastrellae
Halopseudomonas_litoralis|2SM5	GCF_900105005.1	Bacteria-Proteobacteria-Gammaproteobacteria-Pseudomonadales-Pseudomonadaceae-Halopseudomonas-Halopseudomonas_litoralis
Pseudomonas_psychrotolerans|SDS18	GCF_001991015.1	Bacteria-Proteobacteria-Gammaproteobacteria-Pseudomonadales-Pseudomonadaceae-Pseudomonas-Pseudomonas_psychrotolerans
Pseudomonas_taeanensis|MS_3	GCF_000498575.2	Bacteria-Proteobacteria-Gammaproteobacteria-Pseudomonadales-Pseudomonadaceae-Pseudomonas-Pseudomonas_taeanensis
Pseudomonas_fluvialis|CCM8778	GCF_014635625.1	Bacteria-Proteobacteria-Gammaproteobacteria-Pseudomonadales-Pseudomonadaceae-Pseudomonas-Pseudomonas_fluvialis
Pseudomonas_bohemica|IA19	GCF_002934685.1	Bacteria-Proteobacteria-Gammaproteobacteria-Pseudomonadales-Pseudomonadaceae-Pseudomonas-Pseudomonas_bohemica
Pseudomonas_anguilliseptica|DSM12111	GCF_900105355.1	Bacteria-Proteobacteria-Gammaproteobacteria-Pseudomonadales-Pseudomonadaceae-Pseudomonas-Pseudomonas_anguilliseptica
Marinobacter_lutaoensis|T5054	GCF_001981305.1	Bacteria-Proteobacteria-Gammaproteobacteria-Pseudomonadales-Marinobacteraceae-Marinobacter-Marinobacter_lutaoensis
Thiopseudomonas_denitrificans|DSM28679	GCF_004363035.1	Bacteria-Proteobacteria-Gammaproteobacteria-Pseudomonadales-Pseudomonadaceae-Thiopseudomonas-Thiopseudomonas_denitrificans

		Marinobacter_bohaiensis|T17	GCF_003258515.1	Bacteria-Proteobacteria-Gammaproteobacteria-Pseudomonadales-Marinobacteraceae-Marinobacter-Marinobacter_bohaiensis
		Pseudomonas_alcaligenes|NEB585	GCF_001597285.1	Bacteria-Proteobacteria-Gammaproteobacteria-Pseudomonadales-Pseudomonadaceae-Pseudomonas-Pseudomonas_alcaligenes
		Pseudomonas_taiwanensis|DSM21245	GCF_000425785.1	Bacteria-Proteobacteria-Gammaproteobacteria-Pseudomonadales-Pseudomonadaceae-Pseudomonas-Pseudomonas_taiwanensis
		Marinobacter_lipolyticus|SM19	GCF_000397065.2	Bacteria-Proteobacteria-Gammaproteobacteria-Pseudomonadales-Marinobacteraceae-Marinobacter-Marinobacter_lipolyticus
		Marinobacter_fonticola|CS1212	GCF_008122265.1	Bacteria-Proteobacteria-Gammaproteobacteria-Pseudomonadales-Marinobacteraceae-Marinobacter-Marinobacter_fonticola
		Halopseudomonas_xinjiangensis|NRRLB_51270	GCF_900104945.1	Bacteria-Proteobacteria-Gammaproteobacteria-Pseudomonadales-Pseudomonadaceae-Halopseudomonas-Halopseudomonas_xinjiangensis
		Halopseudomonas_formosensis|JCM18415	GCF_900115905.1	Bacteria-Proteobacteria-Gammaproteobacteria-Pseudomonadales-Pseudomonadaceae-Halopseudomonas-Halopseudomonas_formosensis
		Marinobacter_persicus|IBRC_M10445	GCF_900114155.1	Bacteria-Proteobacteria-Gammaproteobacteria-Pseudomonadales-Marinobacteraceae-Marinobacter-Marinobacter_persicus
		Marinobacter_segnicrescens|CGMCC1.6489	GCF_900111555.1	Bacteria-Proteobacteria-Gammaproteobacteria-Pseudomonadales-Marinobacteraceae-Marinobacter-Marinobacter_segnicrescens
		Pseudomonas_yangmingensis|DSM24213	GCF_900114825.1	Bacteria-Proteobacteria-Gammaproteobacteria-Pseudomonadales-Pseudomonadaceae-Pseudomonas-Pseudomonas_yangmingensis
		Marinobacter_profundi|PWS21	GCF_002744715.1	Bacteria-Proteobacteria-Gammaproteobacteria-Pseudomonadales-Marinobacteraceae-Marinobacter-Marinobacter_profundi
		Halopseudomonas_xiamenensis|PX1	GCF_014219065.1	Bacteria-Proteobacteria-Gammaproteobacteria-Pseudomonadales-Pseudomonadaceae-Halopseudomonas-Halopseudomonas_xiamenensis
		Pseudomonas_cuatrocienegasensis|CIP109853	GCF_900110925.1	Bacteria-Proteobacteria-Gammaproteobacteria-Pseudomonadales-Pseudomonadaceae-Pseudomonas-Pseudomonas_cuatrocienegasensis
		Marinobacter_oulmenensis|DSM22359	GCF_014201735.1	Bacteria-Proteobacteria-Gammaproteobacteria-Pseudomonadales-Marinobacteraceae-Marinobacter-Marinobacter_oulmenensis
		Halopseudomonas_salegens|CECT8338	GCF_900105655.1	Bacteria-Proteobacteria-Gammaproteobacteria-Pseudomonadales-Pseudomonadaceae-Halopseudomonas-Halopseudomonas_salegens
		Pseudomonas_seleniipraecipitans|LMG25475	GCF_900102335.1	Bacteria-Proteobacteria-Gammaproteobacteria-Pseudomonadales-Pseudomonadaceae-Pseudomonas-Pseudomonas_seleniipraecipitans
		Pseudomonas_japonica|NBRC103040	GCF_000730585.1	Bacteria-Proteobacteria-Gammaproteobacteria-Pseudomonadales-Pseudomonadaceae-Pseudomonas-Pseudomonas_japonica
		Marinobacter_salinexigens|ZYF650	GCF_008370345.1	Bacteria-Proteobacteria-Gammaproteobacteria-Pseudomonadales-Marinobacteraceae-Marinobacter-Marinobacter_salinexigens
**SULFOLOBALES**	27	Acidianus_ambivalens|DSM3772	GCF_009428885.1	Archaea-Crenarchaeota-Thermoprotei-Sulfolobales-Sulfolobaceae-Acidianus-Acidianus_ambivalens
		Acidianus_ambivalens|LEI10	GCF_009729015.1	Archaea-Crenarchaeota-Thermoprotei-Sulfolobales-Sulfolobaceae-Acidianus-Acidianus_ambivalens
		Acidianus_brierleyi|DSM1651	GCF_003201835.2	Archaea-Crenarchaeota-Thermoprotei-Sulfolobales-Sulfolobaceae-Acidianus-Acidianus_brierleyi
		Acidianus_copahuensis|ALE1	GCF_000632495.1	Archaea-Crenarchaeota-Thermoprotei-Sulfolobales-Sulfolobaceae-Acidianus-Acidianus_copahuensis
		Acidianus_hospitalis|W1	GCF_000213215.1	Archaea-Crenarchaeota-Thermoprotei-Sulfolobales-Sulfolobaceae-Acidianus-Acidianus_hospitalis
		Acidianus_infernus|DSM3191	GCF_009729545.1	Archaea-Crenarchaeota-Thermoprotei-Sulfolobales-Sulfolobaceae-Acidianus-Acidianus_infernus
		Acidianus_manzaensis|YN_25	GCF_002116695.1	Archaea-Crenarchaeota-Thermoprotei-Sulfolobales-Sulfolobaceae-Acidianus-Acidianus_manzaensis
		Acidianus_sulfidivorans|JP7	GCF_003201765.2	Archaea-Crenarchaeota-Thermoprotei-Sulfolobales-Sulfolobaceae-Acidianus-Acidianus_sulfidivorans
		Metallosphaera_cuprina|Ar_4	GCF_000204925.1	Archaea-Crenarchaeota-Thermoprotei-Sulfolobales-Sulfolobaceae-Metallosphaera-Metallosphaera_cuprina

		Metallosphaera_hakonensis|HO1_1	GCF_003201675.2	Archaea-Crenarchaeota-Thermoprotei-Sulfolobales-Sulfolobaceae-Metallosphaera-Metallosphaera_hakonensis
		Metallosphaera_hakonensis|JCM8857	GCF_001315825.1	Archaea-Crenarchaeota-Thermoprotei-Sulfolobales-Sulfolobaceae-Metallosphaera-Metallosphaera_hakonensis
		Metallosphaera_javensis|AS_7	Metallosphaera_	Archaea-Crenarchaeota-Thermoprotei-Sulfolobales-Sulfolobaceae-Metallosphaera-Metallosphaera_javensis
		Metallosphaera_prunae|Ron12II	GCF_005222525.1	Archaea-Crenarchaeota-Thermoprotei-Sulfolobales-Sulfolobaceae-Metallosphaera-Metallosphaera_prunae
		Metallosphaera_tengchongensis|Ric_A	GCF_013343295.1	Archaea-Crenarchaeota-Thermoprotei-Sulfolobales-Sulfolobaceae-Metallosphaera-Metallosphaera_tengchongensis
		Metallosphaera_yellowstonensis|MK1	GCF_000243315.1	Archaea-Crenarchaeota-Thermoprotei-Sulfolobales-Sulfolobaceae-Metallosphaera-Metallosphaera_yellowstonensis
		Saccharolobus_caldissimus|JCM32116	GCF_020886315.1	Archaea-Crenarchaeota-Thermoprotei-Sulfolobales-Sulfolobaceae-Saccharolobus-Saccharolobus_caldissimus
		Saccharolobus_shibatae|B12	GCF_019175345.1	Archaea-Crenarchaeota-Thermoprotei-Sulfolobales-Sulfolobaceae-Saccharolobus-Saccharolobus_shibatae
		Saccharolobus_solfataricus|P1	GCF_900079115.1	Archaea-Crenarchaeota-Thermoprotei-Sulfolobales-Sulfolobaceae-Saccharolobus-Saccharolobus_solfataricus
		Saccharolobus_solfataricus|SULM	GCF_003852155.1	Archaea-Crenarchaeota-Thermoprotei-Sulfolobales-Sulfolobaceae-Saccharolobus-Saccharolobus_solfataricus
		Stygiolobus_azoricus|FC6	GCF_009729035.1	Archaea-Crenarchaeota-Thermoprotei-Sulfolobales-Sulfolobaceae-Stygiolobus-Stygiolobus_azoricus
		Stygiolobus_sp.|KN_1	GCF_019704315.1	Archaea-Crenarchaeota-Thermoprotei-Sulfolobales-Sulfolobaceae-Stygiolobus-Stygiolobus_sp.
		Sulfodiicoccus_acidiphilus|HS_1	GCF_003967175.1	Archaea-Crenarchaeota-Thermoprotei-Sulfolobales-Sulfolobaceae-Sulfodiicoccus-Sulfodiicoccus_acidiphilus
		Sulfodiicoccus_acidiphilus|JCM31740	GCF_014648235.1	Archaea-Crenarchaeota-Thermoprotei-Sulfolobales-Sulfolobaceae-Sulfodiicoccus-Sulfodiicoccus_acidiphilus
		Sulfolobus_acidocaldarius|DG1	GCF_002215565.1	Archaea-Crenarchaeota-Thermoprotei-Sulfolobales-Sulfolobaceae-Sulfolobus-Sulfolobus_acidocaldarius
		Sulfolobus_acidocaldarius|DSM639	GCF_000012285.1	Archaea-Crenarchaeota-Thermoprotei-Sulfolobales-Sulfolobaceae-Sulfolobus-Sulfolobus_acidocaldarius
		Sulfolobus_islandicus|L.D.8.5	GCF_000024305.1	Archaea-Crenarchaeota-Thermoprotei-Sulfolobales-Sulfolobaceae-Sulfolobus-Sulfolobus_islandicus
		Sulfuracidifex_metallicus|DSM6482	GCF_009729515.1	Archaea-Crenarchaeota-Thermoprotei-Sulfolobales-Sulfolobaceae-Sulfuracidifex-Sulfuracidifex_metallicus
		Sulfuracidifex_metallicus|JCM9184	GCF_001316045.1	Archaea-Crenarchaeota-Thermoprotei-Sulfolobales-Sulfolobaceae-Sulfuracidifex-Sulfuracidifex_metallicus
		Sulfuracidifex_tepidarius|IC_006	GCF_008326425.1	Archaea-Crenarchaeota-Thermoprotei-Sulfolobales-Sulfolobaceae-Sulfuracidifex-Sulfuracidifex_tepidarius
		Sulfuracidifex_tepidarius|IC_007	GCF_008326385.1	Archaea-Crenarchaeota-Thermoprotei-Sulfolobales-Sulfolobaceae-Sulfuracidifex-Sulfuracidifex_tepidarius
		Sulfuracidifex_tepidarius|JCM16833	GCF_001316085.1	Archaea-Crenarchaeota-Thermoprotei-Sulfolobales-Sulfolobaceae-Sulfuracidifex-Sulfuracidifex_tepidarius
		Sulfurisphaera_ohwakuensis|DSM12421	GCF_014202105.1	Archaea-Crenarchaeota-Thermoprotei-Sulfolobales-Sulfolobaceae-Sulfurisphaera-Sulfurisphaera_ohwakuensis
		Sulfurisphaera_ohwakuensis|TA_1	GCF_009729055.1	Archaea-Crenarchaeota-Thermoprotei-Sulfolobales-Sulfolobaceae-Sulfurisphaera-Sulfurisphaera_ohwakuensis
		Sulfurisphaera_tokodaii|7	GCF_000011205.1	Archaea-Crenarchaeota-Thermoprotei-Sulfolobales-Sulfolobaceae-Sulfurisphaera-Sulfurisphaera_tokodaii
**THERMOCOCCALES**	65	Thermococcus_barophilus|MP	GCF_000151105.2	Archaea-Euryarchaeota-Thermococci-Thermococcales-Thermococcaceae-Thermococcus-Thermococcus_barophilus
		Thermococcus_thioreducens|OGL_20P	GCF_900109425.1	Archaea-Euryarchaeota-Thermococci-Thermococcales-Thermococcaceae-Thermococcus-Thermococcus_thioreducens

Thermococcus_stetteri|DSM5262	GCF_017873335.1	Archaea-Euryarchaeota-Thermococci-Thermococcales-Thermococcaceae-Thermococcus-Thermococcus_stetteri
Pyrococcus_furiosus|DSM3638	GCF_000007305.1	Archaea-Euryarchaeota-Thermococci-Thermococcales-Thermococcaceae-Pyrococcus-Pyrococcus_furiosus
Thermococcus_pacificus|P_4	GCF_002214485.1	Archaea-Euryarchaeota-Thermococci-Thermococcales-Thermococcaceae-Thermococcus-Thermococcus_pacificus
Thermococcus_thioreducens|OGL_20P	GCF_002214545.1	Archaea-Euryarchaeota-Thermococci-Thermococcales-Thermococcaceae-Thermococcus-Thermococcus_thioreducens
Thermococcus_guaymasensis|DSM11113	GCF_000816105.1	Archaea-Euryarchaeota-Thermococci-Thermococcales-Thermococcaceae-Thermococcus-Thermococcus_guaymasensis
Thermococcus_piezophilus|CDGS	GCF_001647085.1	Archaea-Euryarchaeota-Thermococci-Thermococcales-Thermococcaceae-Thermococcus-Thermococcus_piezophilus
Thermococcus_sibiricus|MM739	GCF_000022545.1	Archaea-Euryarchaeota-Thermococci-Thermococcales-Thermococcaceae-Thermococcus-Thermococcus_sibiricus
Pyrococcus_yayanosii|CH1	GCF_000215995.1	Archaea-Euryarchaeota-Thermococci-Thermococcales-Thermococcaceae-Pyrococcus-Pyrococcus_yayanosii
Thermococcus_litoralis|DSM5473	GCF_000246985.2	Archaea-Euryarchaeota-Thermococci-Thermococcales-Thermococcaceae-Thermococcus-Thermococcus_litoralis
Thermococcus_aciditolerans|SY113	GCF_008152015.1	Archaea-Euryarchaeota-Thermococci-Thermococcales-Thermococcaceae-Thermococcus-Thermococcus_aciditolerans
Thermococcus_onnurineus|NA1	GCF_000018365.1	Archaea-Euryarchaeota-Thermococci-Thermococcales-Thermococcaceae-Thermococcus-Thermococcus_onnurineus
Thermococcus_peptonophilus|OG_1	GCF_001592435.1	Archaea-Euryarchaeota-Thermococci-Thermococcales-Thermococcaceae-Thermococcus-Thermococcus_peptonophilus
Thermococcus_aggregans|TY	GCF_024022995.1	Archaea-Euryarchaeota-Thermococci-Thermococcales-Thermococcaceae-Thermococcus-Thermococcus_aggregans
Palaeococcus_ferrophilus|DSM13482	GCF_000966265.1	Archaea-Euryarchaeota-Thermococci-Thermococcales-Thermococcaceae-Palaeococcus-Palaeococcus_ferrophilus
Palaeococcus_pacificus|DY20341	GCF_000725425.1	Archaea-Euryarchaeota-Thermococci-Thermococcales-Thermococcaceae-Palaeococcus-Palaeococcus_pacificus
Pyrococcus_furiosus|DSM3638	GCF_008245085.1	Archaea-Euryarchaeota-Thermococci-Thermococcales-Thermococcaceae-Pyrococcus-Pyrococcus_furiosus
Thermococcus_celericrescens|DSM17994	GCF_001484195.1	Archaea-Euryarchaeota-Thermococci-Thermococcales-Thermococcaceae-Thermococcus-Thermococcus_celericrescens
Pyrococcus_kukulkanii|NCB100	GCF_001577775.1	Archaea-Euryarchaeota-Thermococci-Thermococcales-Thermococcaceae-Pyrococcus-Pyrococcus_kukulkanii
Thermococcus_thioreducens|DSM14981	GCF_001418715.1	Archaea-Euryarchaeota-Thermococci-Thermococcales-Thermococcaceae-Thermococcus-Thermococcus_thioreducens
Pyrococcus_horikoshii|OT3	GCF_000011105.1	Archaea-Euryarchaeota-Thermococci-Thermococcales-Thermococcaceae-Pyrococcus-Pyrococcus_horikoshii
Thermococcus_chitonophagus|GC74	GCF_002214605.1	Archaea-Euryarchaeota-Thermococci-Thermococcales-Thermococcaceae-Thermococcus-Thermococcus_chitonophagus
Thermococcus_eurythermalis|A501	GCF_000769655.1	Archaea-Euryarchaeota-Thermococci-Thermococcales-Thermococcaceae-Thermococcus-Thermococcus_eurythermalis
Thermococcus_profundus|DT5432	GCF_002214585.1	Archaea-Euryarchaeota-Thermococci-Thermococcales-Thermococcaceae-Thermococcus-Thermococcus_profundus
Thermococcus_siculi|RG_20	GCF_002214505.1	Archaea-Euryarchaeota-Thermococci-Thermococcales-Thermococcaceae-Thermococcus-Thermococcus_siculi
Pyrococcus_abyssi|Orsay	GCF_000195935.2	Archaea-Euryarchaeota-Thermococci-Thermococcales-Thermococcaceae-Pyrococcus-Pyrococcus_abyssi
Thermococcus_radiotolerans|EJ2	GCF_002214565.1	Archaea-Euryarchaeota-Thermococci-Thermococcales-Thermococcaceae-Thermococcus-Thermococcus_radiotolerans
Thermococcus_celer|Vu13	GCF_002214365.1	Archaea-Euryarchaeota-Thermococci-Thermococcales-Thermococcaceae-Thermococcus-Thermococcus_celer

Thermococcus_henrietii|EXT12c	GCF_900198835.1	Archaea-Euryarchaeota-Thermococci-Thermococcales-Thermococcaceae-Thermococcus-Thermococcus_henrietii
Thermococcus_chitonophagus|1	GCF_900012635.1	Archaea-Euryarchaeota-Thermococci-Thermococcales-Thermococcaceae-Thermococcus-Thermococcus_chitonophagus
Thermococcus_alcaliphilus|AEDII12	GCF_024054535.1	Archaea-Euryarchaeota-Thermococci-Thermococcales-Thermococcaceae-Thermococcus-Thermococcus_alcaliphilus
Thermococcus_barossii|SHCK_94	GCF_002214465.1	Archaea-Euryarchaeota-Thermococci-Thermococcales-Thermococcaceae-Thermococcus-Thermococcus_barossii
Thermococcus_nautili|30_1	GCF_000585495.1	Archaea-Euryarchaeota-Thermococci-Thermococcales-Thermococcaceae-Thermococcus-Thermococcus_nautili
Thermococcus_gammatolerans|EJ3+DSM15229	GCF_000022365.1	Archaea-Euryarchaeota-Thermococci-Thermococcales-Thermococcaceae-Thermococcus-Thermococcus_gammatolerans
Thermococcus_indicus|IOH1	GCF_006274605.1	Archaea-Euryarchaeota-Thermococci-Thermococcales-Thermococcaceae-Thermococcus-Thermococcus_indicus
Thermococcus_gorgonarius|W_12	GCF_002214385.1	Archaea-Euryarchaeota-Thermococci-Thermococcales-Thermococcaceae-Thermococcus-Thermococcus_gorgonarius
Thermococcus_kodakarensis|KOD1	GCF_000009965.1	Archaea-Euryarchaeota-Thermococci-Thermococcales-Thermococcaceae-Thermococcus-Thermococcus_kodakarensis
Thermococcus_camini|IRI35c	GCF_904067545.1	Archaea-Euryarchaeota-Thermococci-Thermococcales-Thermococcaceae-Thermococcus-Thermococcus_camini
Thermococcus_bergensis|T7324	GCF_020386975.1	Archaea-Euryarchaeota-Thermococci-Thermococcales-Thermococcaceae-Thermococcus-Thermococcus_bergensis
Thermococcus_paralvinellae|ES1	GCF_000517445.1	Archaea-Euryarchaeota-Thermococci-Thermococcales-Thermococcaceae-Thermococcus-Thermococcus_paralvinellae
Thermococcus_cleftensis|CL1	GCF_000265525.1	Archaea-Euryarchaeota-Thermococci-Thermococcales-Thermococcaceae-Thermococcus-Thermococcus_cleftensis
Thermococcus_zilligii|AN1	GCF_000258515.1	"Archaea-Euryarchaeota-Thermococci-Thermococcales-Thermococcaceae-Thermococcus-Thermococcus_zilligii

Table S4. List of the 63 protein families from *Bacillales*. For each protein family, the number of homologous sequences, the ID of the monomer used as reference from the PDB, its resolution in Å, and the source organism are provided.

Protein family ID	Number of sequences	PDB ID	Resolution (Å)	Source organism
P82599	22	1C75	0.97	*Sporosarcina pasteurii*
Q9S3L6	10	1EA7	0.93	*Lysinibacillus sphaericus*
P29600	23	1GCI	0.78	*Lederbergia lentus*
P10245	113	1IR0	1	*Bacillus thermoproteolyticus*
P0A006	98	1JF8	1.12	*Staphylococcus aureus*
P54464	82	1NG6	1.4	*Bacillus subtilis*
Q9X519	98	1O98	1.4	*Geobacillus stearothermophilus*
P0A078	40	1QXY	1.04	*Staphylococcus aureus*
P84136	19	1SFS	1.07	*Geobacillus stearothermophilus*
Q5KWF3	52	1U9C	1.35	*Geobacillus stearothermophilus*
P32396	93	2H1V	1.2	*Bacillus subtilis*
P17893	93	2P5K	1	*Bacillus subtilis*
P39759	16	2QIK	1.35	*Bacillus subtilis*
Q7A1I3	92	2W5Q	1.2	*Staphylococcus aureus*
P0A017	88	2W9H	1.48	*Staphylococcus aureus*
A0A1T3V7R2	30	2XOD	0.96	*Bacillus anthracis*
A9JQL9	21	3ACX	1.31	*Staphylococcus aureus*
O35031	31	3BR8	1.33	*Bacillus subtilis*
A0A0H3JM89	35	3EIX	1.35	*Staphylococcus aureus subsp. aureus N315*
O32218	33	3GHA	1.4	*Bacillus subtilis*
Q99UN8	98	3IM9	1.46	*Staphylococcus aureus subsp. aureus Mu50*
E3SET7	16	3PKV	1.34	*Paenibacillus polymyxa*
P82597	24	3RM3	1.2	*Bacillus sp. H-257*
P00780	43	3UNX	1.26	*Bacillus licheniformis*
P17906	43	3W43	1.22	*Bacillus subtilis subsp. subtilis str. 168*
P39579	14	4BPF	1.01	*Bacillus subtilis*
A5H1I6	6	4CD8	1.47	*Alicyclobacillus acidocaldarius*
P39155	57	4ETN	1.1	*Bacillus subtilis*
A0A6L7H5L6	67	4HDE	1.317	*Bacillus anthracis*
G8N3E1	57	4HE6	1.1	*Geobacillus thermoleovorans CCB_US3_UF5*
Q73EI2	7	4JG2	1.3	*Bacillus cereus ATCC 10987*
Q8GCA8	100	4MLV	1.455	*Priestia megaterium*
A0A0H3JTS3	36	4NNO	1.174	*Staphylococcus aureus subsp. aureus Mu50*
P16304	101	4QBG	1.37	*Bacillus subtilis subsp. subtilis str. 168*
Q8NWR0	179	4X9X	1.199	*Staphylococcus aureus subsp. aureus MW2*
O31785	57	4YZR	1.35	*Bacillus subtilis subsp. subtilis str. 168*
Q8Y5E5	20	5HQH	1.32	*Listeria monocytogenes EGD-e*
Q739M5	10	5JDA	1.401	*Bacillus cereus ATCC 10987*
P00800	29	5JVI	1.12	*Bacillus thermoproteolyticus*
Q8EP31	17	5LAU	1.35	*Oceanobacillus iheyensis HTE831*

O31815	7	5ONK	1.03	*Bacillus subtilis*
A0A2A5LBI6	31	5ONN	1.4	*Paenibacillus lautus*
P37965	112	5T9C	1.48	*Bacillus subtilis subsp. subtilis str. 168*
Q45462	48	6EYG	1.42	*Bacillus subtilis*
Q81G35	44	6FSG	1.27	*Bacillus cereus ATCC 14579*
I3DZK9	33	6FYJ	1.3	*Bacillus methanolicus*
P43133	29	6GHX	1.156	*Geobacillus stearothermophilus*
P39072	70	6GZ8	1	*Bacillus subtilis subsp. subtilis str. 168*
Q9RL81	100	6IHR	1.348	*Staphylococcus aureus*
D5DH82	12	6MU9	0.97	*Priestia megaterium DSM 319*
Q2FY41	99	6RJI	1.48	*Staphylococcus aureus subsp. aureus NCTC 8325*
P0A031	102	6RVQ	1.136	*Staphylococcus aureus*
P84138	92	6T0Y	1.2	*Geobacillus stearothermophilus*
P0A3V0	50	6TCI	1.47	*Bacillus cereus ATCC 14579*
A0A250DVN1	100	6TG6	1.3	*Geobacillus stearothermophilus*
P54427	11	6W5E	1.3	*Bacillus subtilis subsp. subtilis str. 168*
A0A6L7H2K2	66	7BKF	1.139	*Bacillus anthracis*
O32192	196	7CX5	1.07	*Bacillus subtilis subsp. subtilis str. 168*
A0A060M4R1	103	7EV5	1.44	*Alkalihalobacillus lehensis G1*
O06994	177	7LV6	1.1	*Bacillus subtilis subsp. subtilis str. 168*
A0A0D6HIR7	92	7O4M	1.3	*Staphylococcus aureus*
W8FKE7	9	7R25	0.87	*Bacillus pumilus*
Q2FZ58	92	7RM7	1.025	*Staphylococcus aureus*

Table S5. List of the 31 protein families from *Bacteroidales*. For each protein family, the number of homologous sequences, the ID of the monomer used as reference from the PDB, its resolution in Å, and the source organism are provided.

Protein family ID	Number of sequences	PDB ID	Resolution (Å)	Source organism
Q7MXD1	93	2NQW	1.30	*Porphyromonas gingivalis W83*
Q8A090	57	2RBK	1.00	*Bacteroides thetaiotaomicron VPI-5482*
A0A380Z3E8	63	3EUR	1.30	*Bacteroides fragilis NCTC 9343*
Q8A2Z3	28	3I10	1.35	*Bacteroides thetaiotaomicron VPI-5482*
Q5LGM0	24	3I4G	1.35	*Bacteroides fragilis NCTC 9343*
Q89YQ6	9	3IKW	1.30	*Bacteroides thetaiotaomicron*
A6L326	10	3JQ0	1.13	*Phocaeicola vulgatus ATCC 8482*
A6L0Y5	57	3LAX	1.43	*Phocaeicola vulgatus ATCC 8482*
A6LB22	84	3LWX	1.10	*Parabacteroides distasonis ATCC 8503*
Q8A577	24	3MCX	1.49	*Bacteroides thetaiotaomicron VPI-5482*
A5ZFJ0	8	3NO2	1.35	*Bacteroides caccae ATCC 43185*
A7M120	10	3OYV	1.25	*Bacteroides ovatus ATCC 8483*
A0A0K6BSL6	10	4FR9	1.20	*Bacteroides fragilis NCTC 9343*
Q8A4P9	82	4FUU	1.30	*Bacteroides thetaiotaomicron VPI-5482*
Q5LCG5	92	4JDU	1.47	*Bacteroides fragilis NCTC 9343*
Q5LI79	70	4JJA	1.30	*Bacteroides fragilis NCTC 9343*
A6KZT0	17	4LER	1.42	*Phocaeicola vulgatus ATCC 8482*
A7M2H0	20	4ORL	1.40	*Bacteroides ovatus ATCC 8483*
A7V5T8	42	4Q68	1.07	*Bacteroides uniformis ATCC 8492*
A5ZGP5	8	4QHW	1.35	*Bacteroides caccae ATCC 43185*
A6L3L5	7	4ZGF	1.00	*Phocaeicola vulgatus ATCC 8482*
A7LXT5	16	5E75	1.36	*Bacteroides ovatus ATCC 8483*
Q8A7C8	9	5G2U	1.43	*Bacteroides thetaiotaomicron VPI-5482*
Q89YS3	69	5G2V	1.39	*Bacteroides thetaiotaomicron VPI-5482*
Q8A921	14	5MT2	1.41	*Bacteroides thetaiotaomicron VPI-5482*
Q8A1H5	15	5MUL	1.39	*Bacteroides thetaiotaomicron VPI-5482*
Q8A1H4	19	5NOA	1.26	*Bacteroides thetaiotaomicron*
E2NI36	35	5TFQ	1.07	*Bacteroides cellulosilyticus DSM 14838*
Q8A0N4	13	6T8I	1.4	*Bacteroides thetaiotaomicron VPI-5482*
Q8A3D9	9	7BLK	1.06	*Bacteroides thetaiotaomicron VPI-5482*
U6RD63	153	7ZGM	1.43	*Phocaeicola massiliensis B84634 = Timone 84634 = DSM 17679 = JCM 13223*

Table S6. List of the 56 protein families from *Corynebacteriales*. For each protein family, the number of homologous sequences, the ID of the monomer used as reference from the PDB, its resolution in Å, and the source organism are provided.

Protein family ID	Number of sequences	PDB ID	Resolution (Å)	Source organism
P9WG65	13	1LU4	1.12	*Mycobacterium tuberculosis*
O06441	67	1LZL	1.30	*Rhodococcus sp.*
P9WGM3	66	1S5N	1.48	*Mycobacterium tuberculosis H37Rv*
P9WK83	95	1W66	1.08	*Mycobacterium tuberculosis H37Rv*
A0QRS3	35	2HW2	1.45	*Mycolicibacterium smegmatis*
P9WPY3	83	2IYV	1.35	*Mycobacterium tuberculosis H37Rv*
O07756	19	2JEK	1.38	*Mycobacterium tuberculosis H37Rv*
A0QTQ6	25	2JFR	0.83	*Mycolicibacterium smegmatis MC2 155*
P9WPY5	100	2O0B	1.15	*Mycobacterium tuberculosis*
P9WMM5	100	2Y88	1.33	*Mycobacterium tuberculosis H37Rv*
Q8NR27	43	3GJY	1.47	*Corynebacterium glutamicum ATCC 13032*
P9WPP1	134	3IVY	1.35	*Mycobacterium tuberculosis H37Rv*
O53168	27	3NE0	1.00	*Mycobacterium tuberculosis H37Rv*
P9WKD3	31	3NY4	1.22	*Mycobacterium tuberculosis*
P9WK19	100	3PKC	1.47	*Mycobacterium tuberculosis*
P9WFX7	97	3QJA	1.29	*Mycobacterium tuberculosis*
P9WPC9	99	3WDB	1.37	*Mycobacterium tuberculosis*
P9WG47	87	4G3N	1.40	*Mycobacterium tuberculosis H37Rv*
P95106	115	4HS1	0.87	*Mycobacterium tuberculosis*
O53223	112	4HU2	1.46	*Mycobacterium tuberculosis*
P65315	28	4QA8	1.10	*Mycobacterium tuberculosis variant bovis AF2122/97*
A0QYB3	14	4RS3	1.40	*Mycolicibacterium smegmatis MC2 155*
A0QWX6	94	4TM7	1.39	*Mycolicibacterium smegmatis MC2 155*
O33336	95	4U89	1.40	*Mycobacterium tuberculosis H37Rv*
A1TH50	33	4U98	1.15	*Mycolicibacterium vanbaalenii PYR-1*
P9WFQ9	99	4WPK	0.98	*Mycobacterium tuberculosis H37Rv*
A0A067HWN0	95	4WXT	1.20	*Mycobacterium avium subsp. hominissuis 3388*
G7CI90	28	4XDQ	1.35	*Mycolicibacterium thermoresistibile ATCC 19527*
K0V1M6	97	4XIJ	1.45	*Mycolicibacterium fortuitum subsp. fortuitum DSM 46621 = ATCC 6841*
A0QU01	75	4Y9I	1.50	*Mycolicibacterium smegmatis MC2 155*
P9WFV1	99	5AGR	1.30	*Mycobacterium tuberculosis H37Rv*
Q8NTB8	39	5B4Z	1.30	*Corynebacterium glutamicum ATCC 13032*
P9WKJ1	118	5DJH	1.45	*Mycobacterium tuberculosis*
P9WID9	56	5EPF	1.35	*Mycobacterium tuberculosis H37Rv*
P0A3G2	13	5FLK	0.99	*Rhodococcus rhodochrous*
A0QUZ2	98	5GGB	1.10	*Mycolicibacterium smegmatis MC2 155*
B2HN69	61	5MSO	1.20	*Mycobacterium marinum M*
P9WNX1	80	5SD5	1.20	*Mycobacterium tuberculosis H37Rv*
P9WQN8	142	5VNS	1.45	*Mycobacterium tuberculosis*
A0A0T9WNE5	25	5WP2	1.44	*Mycobacterium tuberculosis*

P9WQ13	58	5ZHZ	1.18	*Mycobacterium tuberculosis H37Rv*
A0R2K6	66	5ZRC	1.10	*Mycolicibacterium smegmatis MC2 155*
A0QP43	22	6AJP	1.33	*Mycolicibacterium smegmatis MC2 155*
P9WK17	82	6AS12	1.40	*Mycobacterium tuberculosis*
E0DH37	30	6BO0	1.20	*Corynebacterium matruchotii ATCC 14266*
O06825	48	6D4K	1.32	*Mycobacterium tuberculosis H37Rv*
O53780	17	6E5F	1.37	*Mycobacterium tuberculosis H37Rv*
A0QP47	18	6EF6	1.35	*Mycolicibacterium smegmatis MC2 155*
P96257	101	6H20	1.40	*Mycobacterium tuberculosis H37Rv*
P9WFG7	40	6R3W	1.20	*Mycobacterium tuberculosis*
P9WPP3	73	6T0H	1.18	*Mycobacterium tuberculosis H37Rv*
A0QQF4	24	6T84	1.40	*Mycolicibacterium smegmatis MC2 155*
A0PQG8	79	6UWW	0.92	*Mycobacterium ulcerans Agy99*
B1MHW4	100	6YX3	1.22	*Mycobacteroides abscessus ATCC 19977*
P9WKL5	49	7BGG	1.04	*Mycobacterium tuberculosis H37Rv*
A0A088FL33	53	7V43	1.40	*Rhodococcus coprophilus*

Table S7. List of the 102 protein families from *Enterobacterales*. For each protein family, the number of homologous sequences, the ID of the monomer used as reference from the PDB, its resolution in Å, and the source organism are provided.

Protein family ID	Number of sequences	PDB ID	Resolution (Å)	Source organism
P32173	71	1E5K	1.35	*Escherichia coli K-12*
P27838	60	1EW4	1.40	*Escherichia coli*
P36936	14	1FY2	1.20	*Salmonella enterica subsp. enterica serovar Typhimurium*
P0A6V5	73	1GMX	1.10	*Escherichia coli BL21(DE3)*
Q46822	46	1HZT	1.45	*Escherichia coli*
P0AG82	72	1IXH	0.98	*Escherichia coli*
P0AE67	62	1JBE	1.08	*Escherichia coli*
P0A7Y4	85	1JL1	1.30	*Escherichia coli*
P0AFR4	75	1K7J	1.40	*Escherichia coli*
P17802	87	1KG2	1.20	*Escherichia coli*
P0A6A8	98	1L0I	1.20	*Escherichia coli*
P62593	29	1M40	0.85	*Escherichia coli*
P0ADU5	59	1NNX	1.45	*Escherichia coli*
P0A6C1	76	1QTW	1.02	*Escherichia coli*
Q8ZPK0	50	1S6U	1.38	*Salmonella enterica subsp. enterica serovar Typhimurium str. LT2*
Q8ZKQ1	26	1TJY	1.30	*Salmonella enterica subsp. enterica serovar Typhimurium str. LT2*
P0A8M3	94	1TKE	1.46	*Escherichia coli*
P26602	70	1TT8	1.00	*Escherichia coli*
P0ADE8	95	1VLY	1.30	*Escherichia coli*
P0A6K3	100	1XEO	1.30	*Escherichia coli BL21(DE3)*
A0A384K8I9	29	1Z67	1.45	*Shigella flexneri 2a str. 2457T*
P71278	47	2ABB	1.00	*Enterobacter cloacae*
P0AEE5	49	2FVY	0.92	*Escherichia coli*
P36655	71	2FWH	0.99	*Escherichia coli*
P0AE18	98	2GG2	1.00	*Escherichia coli K-12*
Q6KD95	15	2GZS	1.40	*Escherichia coli*
P69986	91	2JLI	1.13	*Yersinia pestis*
P75966	70	2OML	1.20	*Escherichia coli*
P21338	26	2PQX	1.42	*Escherichia coli*
P0A7D1	98	2PTH	1.20	*Escherichia coli K-12*
P39325	45	2VK2	1.20	*Escherichia coli K-12*
P78067	19	2WLR	1.45	*Escherichia coli*
P14900	96	2X5O	1.46	*Escherichia coli DH5[alpha]*
P0A903	75	2YH5	1.25	*Escherichia coli K-12*
P0A6T9	71	3A7L	1.30	*Escherichia coli K-12*
P04825	74	3B34	1.30	*Escherichia coli K-12*
P08997	50	3CUZ	1.04	*Escherichia coli*
A6THR5	39	3D02	1.30	*Klebsiella pneumoniae subsp. pneumoniae MGH 78578*
P77214	22	3E6Z	1.00	*Escherichia coli*
Q8XAB8	32	3EYE	1.45	*Escherichia coli O157:H7*

P37001	56	3GP6	1.40	*Escherichia coli K-12*
P0AEC6	65	3IQT	1.40	*Escherichia coli CFT073*
Q7CQR3	43	3IR4	1.20	*Salmonella enterica subsp. enterica serovar Typhimurium str. LT2*
Q8ZPF0	21	3M07	1.40	*Salmonella enterica subsp. enterica serovar Typhimurium str. LT2*
Q8ZAR2	78	3MJF	1.47	*Yersinia pestis CO92*
B7LFS5	50	3RX9	1.35	*Escherichia coli 55989*
P67080	85	3SY1	1.47	*Escherichia coli K-12*
H9TUK5	19	4ARU	1.45	*Hafnia alvei*
Q8X5N8	60	4CDP	1.45	*Escherichia coli O157:H7*
P0AEM0	70	4DT4	1.35	*Escherichia coli K-12*
P36649	50	4E9S	1.06	*Escherichia coli K-12*
P12295	95	4EUG	1.40	*Escherichia coli B*
P0AA25	98	4HUA	1.10	*Escherichia coli K-12*
A6TB72	33	4HWM	1.38	*Klebsiella pneumoniae subsp. pneumoniae MGH 78578*
P05050	53	4JHT	1.18	*Escherichia coli K-12*
P0ABQ4	95	4KJJ	1.15	*Escherichia coli K-12*
P74881	79	4LGY	1.48	*Salmonella enterica subsp. enterica serovar Typhimurium str. LT2*
P37355	64	4MXD	1.45	*Escherichia coli K-12*
Q8ZLI9	82	4NMW	1.50	*Salmonella enterica subsp. enterica serovar Typhimurium str. LT2*
A8GFD6	9	4NZC	1.45	*Serratia proteamaculans 568*
Q8VQF4	28	4OXX	1.21	*Citrobacter braakii*
A0A0J9X1Z5	72	4PDN	1.45	*Escherichia coli KTE5*
P37344	69	4QOS	1.42	*Escherichia coli K-12*
P96062	9	4R6Y	1.22	*Salmonella enterica subsp. enterica serovar Typhimurium str. LT2*
P08191	16	4X5P	1.00	*Escherichia coli K-12*
P0AA89	19	4ZVF	1.15	*Escherichia coli K-12*
Q1R1X2	18	5COF	1.35	*Escherichia coli UTI89*
P23694	11	5D7W	1.10	*Serratia marcescens*
P0A6L0	66	5EKY	1.10	*Escherichia coli*
A0A0H2W1T3	56	5EQV	1.45	*Yersinia pestis CO92*
P26281	91	5ETP	1.05	*Escherichia coli*
A0A0R6L508	61	5GRR	1.45	*Escherichia coli*
A6T990	6	5HSG	1.30	*Klebsiella pneumoniae subsp. pneumoniae MGH 78578*
Q7A225	27	5IWH	1.10	*Proteus vulgaris*
A0A0H2V3I0	41	5LP9	0.89	*Shigella flexneri*
Q7CPI7	45	5OLT	1.45	*Salmonella enterica subsp. enterica serovar Typhimurium str. LT2*
P0ADA1	67	5TIF	0.97	*Escherichia coli*
A1JTX8	40	5ULB	1.28	*Yersinia enterocolitica subsp. enterocolitica 8081*
Q56952	47	5UXS	1.42	*Yersinia pestis*
P0ABE7	55	5YO6	1.20	*Escherichia coli*
P22259	84	6AT2	1.44	*Escherichia coli K-12*
A0A061LQM0	13	6BA9	1.40	*Escherichia coli*
C9XTL5	33	6DGA	1.19	*Cronobacter turicensis z3032*
P02911	81	6FT2	1.25	*Salmonella enterica subsp. enterica serovar Typhimurium*

P0C960	56	6GI4	1.35	*Escherichia coli*
A0A0H2Z2W8	9	6MGC	1.35	*Escherichia coli APEC O1*
Q8ZPL9	61	6N9M	1.45	*Salmonella enterica subsp. enterica serovar Typhimurium str. LT2*
A0A0M7FD65	71	6OSX	1.45	*Enterobacter cloacae*
Q8ZQT5	85	6PNV	1.35	*Salmonella enterica subsp. enterica serovar Typhimurium str. LT2-4*
P37634	73	6QE0	1.40	*Escherichia coli*
P0AC84	89	6RZ0	1.20	*Escherichia coli K-12*
P24670	16	6SFG	1.10	*Salmonella enterica subsp. enterica serovar Typhi*
P00959	94	6SPO	1.45	*Escherichia coli*
P00811	26	6T5Y	0.98	*Escherichia coli K-12*
P0A9A6	98	6UMK	1.08	*Escherichia coli*
B4EUK6	9	6Y4E	1.30	*Proteus mirabilis HI4320*
G7LSK3	8	7EHR	1.40	*Brenneria sp. EniD312*
B2VCC3	8	7OSO	1.20	*Erwinia tasmaniensis Et1/99*
P31133	62	7OYY	1.20	*Escherichia coli K-12*
P0AES12	100	7P2M	0.85	*Escherichia coli K-12*
P23869	72	7RFD	1.45	*Escherichia coli*
P02924	50	8ABP	1.02	*Escherichia coli*

Table S8. List of the 85 protein families from *Escherichia*. For each protein family, the number of homologous sequences, the ID of the monomer used as reference from the PDB, its resolution in Å, and the source organism are provided.

Protein family ID	Number of sequences	PDB ID	Resolution (Å)	Source organism
P32173	26	1E5K	1.35	*Escherichia coli K-12*
P27838	26	1EW4	1.40	*Escherichia coli*
P36936	23	1FY2	1.20	*Salmonella enterica subsp. enterica serovar Typhimurium*
P0A6V5	26	1GMX	1.10	*Escherichia coli BL21(DE3)*
Q46822	26	1HZT	1.45	*Escherichia coli*
P0AG82	26	1IXH	0.98	*Escherichia coli*
P0AE67	21	1JBE	1.08	*Escherichia coli*
P0A7Y4	26	1JL1	1.30	*Escherichia coli*
P0AFR4	26	1K7J	1.40	*Escherichia coli*
P17802	26	1KG2	1.20	*Escherichia coli*
P0ADU5	26	1NNX	1.45	*Escherichia coli*
P0A6C1	26	1QTW	1.02	*Escherichia coli*
Q8ZPK0	21	1S6U	1.38	*Salmonella enterica subsp. enterica serovar Typhimurium str. LT2*
Q8ZKQ1	9	1TJY	1.30	*Salmonella enterica subsp. enterica serovar Typhimurium str. LT2*
P0A8M3	26	1TKE	1.46	*Escherichia coli*
P26602	26	1TT8	1.00	*Escherichia coli*
P0ADE8	26	1VLY	1.30	*Escherichia coli*
P0A6K3	26	1XEO	1.30	*Escherichia coli BL21(DE3)*
A0A384K8I9	26	1Z67	1.45	*Shigella flexneri 2a str. 2457T*
P71278	25	2ABB	1.00	*Enterobacter cloacae*
P36655	25	2FWH	0.99	*Escherichia coli*
P0AE18	26	2GG2	1.00	*Escherichia coli K-12*
P75966	26	2OML	1.20	*Escherichia coli*
P21338	24	2PQX	1.42	*Escherichia coli*
P0A7D1	26	2PTH	1.20	*Escherichia coli K-12*
Q8ZRM2	26	2QED	1.45	*Salmonella enterica subsp. enterica serovar Typhimurium str. LT2*
P39325	23	2VK2	1.20	*Escherichia coli K-12*
P78067	25	2WLR	1.45	*Escherichia coli*
P14900	26	2X5O	1.46	*Escherichia coli DH5[alpha]*
P0A903	26	2YH5	1.25	*Escherichia coli K-12*
P0A6T9	26	3A7L	1.30	*Escherichia coli K-12*
P04825	26	3B34	1.30	*Escherichia coli K-12*
P08997	23	3CUZ	1.04	*Escherichia coli*
P77214	19	3E6Z	1.00	*Escherichia coli*
Q8XAB8	16	3EYE	1.45	*Escherichia coli O157:H7*
P37001	24	3GP6	1.40	*Escherichia coli K-12*
P0AEC6	25	3IQT	1.40	*Escherichia coli CFT073*
Q7CQR3	15	3IR4	1.20	*Salmonella enterica subsp. enterica serovar Typhimurium str. LT2*

Q8ZAR2	26	3MJF	1.47	*Yersinia pestis CO92*
P67080	26	3SY1	1.47	*Escherichia coli K-12*
H9TUK5	26	4ARU	1.45	*Hafnia alvei*
Q8X5N8	11	4CDP	1.45	*Escherichia coli O157:H7*
P0AEM0	27	4DT4	1.35	*Escherichia coli K-12*
P76002	22	4DXZ	1.25	*Escherichia coli K-12*
P36649	26	4E9S	1.06	*Escherichia coli K-12*
P12295	25	4EUG	1.40	*Escherichia coli B*
P0AA25	26	4HUA	1.10	*Escherichia coli K-12*
A6TB72	23	4HWM	1.38	*Klebsiella pneumoniae subsp. pneumoniae MGH 78578*
P05050	25	4JHT	1.18	*Escherichia coli K-12*
P74881	26	4LGY	1.48	*Salmonella enterica subsp. enterica serovar Typhimurium str. LT2*
P37355	26	4MXD	1.45	*Escherichia coli K-12*
Q8ZLI9	26	4NMW	1.50	*Salmonella enterica subsp. enterica serovar Typhimurium str. LT2*
A0A0J9X1Z5	26	4PDN	1.45	*Escherichia coli KTE5*
P37344	26	4QOS	1.42	*Escherichia coli K-12*
P08191	16	4X5P	1.00	*Escherichia coli K-12*
P0AA89	14	4ZVF	1.15	*Escherichia coli K-12*
P0A6L0	26	5EKY	1.10	*Escherichia coli*
A0A0H2W1T3	26	5EQV	1.45	*Yersinia pestis CO92*
P26281	26	5ETP	1.05	*Escherichia coli*
Q7A225	11	5IWH	1.10	*Proteus vulgaris*
Q8D072	26	5KWS	1.32	*Yersinia pestis CO92*
A0A0H2V3I0	15	5LP9	0.89	*Shigella flexneri*
A0A1C3NEV1	24	5MX9	1.12	*Escherichia coli*
Q7CPI7	22	5OLT	1.45	*Salmonella enterica subsp. enterica serovar Typhimurium str. LT2*
P0ADA1	23	5TIF	0.97	*Escherichia coli*
Q56952	10	5UXS	1.42	*Yersinia pestis*
P0ABE7	27	5YO6	1.20	*Escherichia coli*
P22259	26	6AT2	1.44	*Escherichia coli K-12*
P0ABQ5	26	6CW7	1.03	*Escherichia coli CFT073*
C9XTL5	19	6DGA	1.19	*Cronobacter turicensis z3032*
P02911	26	6FT2	1.25	*Salmonella enterica subsp. enterica serovar Typhimurium*
P0C960	26	6GI4	1.35	*Escherichia coli*
Q9XB24	25	6KBY	1.10	*Klebsiella pneumoniae*
P77588	7	6MAP	1.08	*Escherichia coli UTI89*
Q8ZPL9	26	6N9M	1.45	*Salmonella enterica subsp. enterica serovar Typhimurium str. LT2*
A0A0M7FD65	26	6OSX	1.45	*Enterobacter cloacae*
Q8ZQT5	26	6PNV	1.42	*Salmonella enterica subsp. enterica serovar Typhimurium str. LT2-4*
P37634	26	6QE0	1.39	*Escherichia coli*
P24670	23	6SFG	1.23	*Salmonella enterica subsp. enterica serovar Typhi*
P00959	26	6SPO	1.20	*Escherichia coli*
P0A9A6	26	6UMK	1.35	*Escherichia coli*
P31133	26	7OYY	1.36	*Escherichia coli K-12*

P0AES12	26	7P2M	1.16	*Escherichia coli K-12*
P23869	26	7RFD	1.35	*Escherichia coli*
P02924	22	8ABP	1.49	*Escherichia coli*

Table S9. List of the 21 protein families from *Hyphomicrobiales*. For each protein family, the number of homologous sequences, the ID of the monomer used as reference from the PDB, its resolution in Å, and the source organism are provided.

Protein family ID	Number of sequences	PDB ID	Resolution (Å)	Source organism
P10955	16	1EW0	1.40	*Sinorhizobium meliloti*
P30960	88	1KNG	1.14	*Bradyrhizobium japonicum*
A9CHJ4	100	1ZCE	1.30	*Agrobacterium fabrum str. C58*
Q988D3	9	3ALJ	1.48	*Mesorhizobium japonicum MAFF 303099*
Q7CX36	113	3IPC	1.30	*Agrobacterium fabrum str. C58*
Q2IR47	15	3SG0	1.20	*Rhodopseudomonas palustris HaA2*
Q92UV8	19	3SZY	1.35	*Sinorhizobium meliloti 1021*
A9CH39	30	4DJA	1.45	*Agrobacterium fabrum str. C58*
Q2IXT5	35	4F06	1.30	*Rhodopseudomonas palustris HaA2*
A9CGI0	9	4RJZ	1.17	*Agrobacterium fabrum str. C58*
B9JKX8	11	4RXT	1.35	*Agrobacterium radiobacter K84*
H7C6E5	85	4W9Z	1.30	*Bradyrhizobium diazoefficiens USDA 110*
Q89VB6	93	4WBR	1.40	*Bradyrhizobium diazoefficiens USDA 110*
C4IR59	24	4ZBG	1.25	*Brucella abortus str. 2308 A*
Q92NR7	48	5C7H	1.30	*Sinorhizobium meliloti 1021*
Q2JZQ5	22	5IBQ	1.20	*Rhizobium etli CFN 42*
A0A452CS122	98	5NFM	0.80	*Sinorhizobium meliloti 2011*
Q2YQX0	95	6CKP	1.15	*Brucella melitensis*
A0A0H3ASL8	76	6RRA	1.40	*Brucella ovis ATCC 25840*
E2RV62	14	6S32	1.40	*Bradyrhizobium elkanii*
Q2IU02	28	7R8T	1.37	*Rhodopseudomonas palustris HaA2*

Table S10. List of the 27 protein families from *Methanococcales*. For each protein family, the number of homologous sequences, the ID of the monomer used as reference from the PDB, its resolution in Å, and the source organism are provided.

Protein family ID	Number of of sequences	PDB ID	Resolution (Å)	Source organism
Q58292	16	1DUS	1.80	*Methanocaldococcus jannaschii*
Q58108	16	1FBN	1.60	*Methanocaldococcus jannaschii*
Q58663	16	1G6H	1.60	*Methanocaldococcus jannaschii*
P54066	8	1XBI	1.45	*Methanocaldococcus jannaschii*
Q58625	16	2EIF	1.80	*Methanocaldococcus jannaschii DSM 2661*
O73948	16	2I1Q	1.90	*Methanococcus voltae*
Q60257	7	2P5D	1.70	*Methanocaldococcus jannaschii*
Q57773	7	2QTD	1.70	*Methanocaldococcus jannaschii DSM 2661*
Q57816	16	2VAP	1.70	*Methanocaldococcus jannaschii DSM 2661*
Q60365	16	2VBU	1.70	*Methanocaldococcus jannaschii*
Q58643	16	2YV1	1.70	*Methanocaldococcus jannaschii DSM 2661*
Q59055	16	2YWJ	1.90	*Methanocaldococcus jannaschii DSM 2661*
Q60343	16	3AJD	1.27	*Methanocaldococcus jannaschii*
Q58586	14	3CFZ	1.70	*Methanocaldococcus jannaschii*
Q6LYF9	15	3E0E	1.60	*Methanococcus maripaludis*
Q58361	15	3GMI	1.91	*Methanocaldococcus jannaschii DSM 2661*
Q58435	16	3GRU	1.60	*Methanocaldococcus jannaschii*
Q58235	16	3PR9	1.95	*Methanocaldococcus jannaschii*
Q58105	16	3T7Z	1.70	*Methanocaldococcus jannaschii*
P81291	12	4KP1	1.80	*Methanocaldococcus jannaschii DSM 2661*
P54050	15	4LQ4	1.75	*Methanocaldococcus jannaschii DSM 2661*
Q57556	15	5HYA	1.90	*Methanocaldococcus jannaschii DSM 2661*
Q57705	16	6DJT	1.64	*Methanocaldococcus jannaschii*
Q6LYQ5	12	6EKG	1.15	*Methanococcus maripaludis*
D5VRB9	16	6IA8	1.90	*Methanocaldococcus infernus*
Q58036	13	7TOM	1.85	*Methanocaldococcus jannaschii*
Q58885	16	7UOF	1.90	*Methanocaldococcus jannaschii*

Table S11. List of the 39 protein families from *Pseudomonadales*. For each protein family, the number of homologous sequences, the ID of the monomer used as reference from the PDB, its resolution in Å, and the source organism are provided.

Protein family ID	Number of sequences	PDB ID	Resolution (Å)	Source organism
Q7SIH2	80	1ATG	1.20	*Azotobacter vinelandii*
Q9I4D4	43	1RTT	1.28	*Pseudomonas aeruginosa PAO1*
Q9HXX5	98	1RW1	1.02	*Pseudomonas aeruginosa PAO1*
Q9HX49	37	1TU9	1.20	*Pseudomonas aeruginosa PAO1*
P02973	13	1X6Z	0.78	*Pseudomonas aeruginosa*
P0A114	83	1ZI8	1.40	*Pseudomonas putida*
Q9I6D2	56	2FGO	1.32	*Pseudomonas aeruginosa*
Q9HYP4	17	2FUP	1.48	*Pseudomonas aeruginosa*
P00282	84	3FSA	0.98	*Pseudomonas aeruginosa*
Q9HUK6	30	3HV8	1.44	*Pseudomonas aeruginosa PAO1*
Q9HXE3	100	3NYC	1.06	*Pseudomonas aeruginosa*
B2Z3V8	6	3SMV	1.38	*Pseudomonas sp. AC2*
Q9I202	195	3SZV	1.45	*Pseudomonas aeruginosa*
P47205	28	3UHM	1.26	*Pseudomonas aeruginosa*
Q9I752	59	3ZHN	1.40	*Pseudomonas aeruginosa PAO1*
Q3K5N8	14	4A9V	1.10	*Pseudomonas fluorescens Pf0-1*
Q9I754	36	4B62	1.45	*Pseudomonas aeruginosa PAO1*
Q9HV14	46	4L8A	1.20	*Pseudomonas aeruginosa PAO1*
G3XD43	15	4NOA	1.25	*Pseudomonas aeruginosa PAO1*
Q9HXB1	8	4PS12	1.25	*Pseudomonas aeruginosa PAO1*
Q9I746	63	4UQX	1.20	*Pseudomonas aeruginosa PAO1*
Q3ZDM6	14	4UTH	1.25	*Pseudomonas putida*
P24735	16	4WZ4	1.05	*Pseudomonas aeruginosa PAO1*
I3UZ52	100	4XFE	1.40	*Pseudomonas putida ND6*
Q9I4L6	8	4ZHW	1.39	*Pseudomonas aeruginosa PAO1*
P0C2B2	15	4ZL8	1.40	*Pseudomonas aeruginosa PAO1*
P00324	100	5K9B	1.17	*Azotobacter vinelandii*
O69052	46	5O37	1.37	*Pseudomonas stutzeri*
B7UZI4	52	6CAX	1.25	*Pseudomonas aeruginosa*
Q9HX72	9	6EDV	1.35	*Pseudomonas aeruginosa PAO1*
G3XD46	17	6HR4	1.19	*Pseudomonas aeruginosa PAO1*
Q9X6V6	19	6I05	1.21	*Pseudomonas aeruginosa*
C3JYL7	20	6NFR	1.00	*Pseudomonas fluorescens*
A0A1H6AD45	20	6SBN	1.09	*Halopseudomonas aestusnigri*
Q88CC1	63	6W1G	1.14	*Pseudomonas putida*
Q9I3B1	74	7CFW	1.31	*Pseudomonas aeruginosa*
P00214	100	7FD1	1.30	*Azotobacter vinelandii*
A0A0H2Z9V8	61	7MQ5	1.25	*Pseudomonas aeruginosa UCBPP-PA14*
Q02PF8	74	7QU5	1.25	*Pseudomonas aeruginosa*

Table S12. List of the 29 protein families from *Sulfolobales*. For each protein family, the number of homologous sequences, the ID of the monomer used as reference from the PDB, its resolution in Å, and the source organism are provided.

Protein family ID	Number of sequences	PDB ID	Resolution (Å)	Source organism
Q53766	19	1JM1	1.11	*Sulfolobus acidocaldarius*
Q97UY8	21	1OXX	1.45	*Saccharolobus solfataricus*
Q54324	9	1RO2	1.6	*Sulfolobus islandicus*
Q974G3	13	1WOL	1.62	*Sulfurisphaera tokodaii str. 7*
F9VP91	34	2E0Q	1.49	*Sulfurisphaera tokodaii*
F9VN79	28	2EHG	1.60	*Sulfurisphaera tokodaii str. 7*
Q970S12	33	2EQA	1.80	*Sulfurisphaera tokodaii*
Q97XP0	17	2RFF	1.40	*Saccharolobus solfataricus P2*
Q980R9	34	2X7B	1.95	*Saccharolobus solfataricus P2*
F9VNL8	29	2YWN	1.60	*Sulfurisphaera tokodaii*
Q96XQ7	34	2Z0M	1.90	*Sulfurisphaera tokodaii*
Q972I2	10	3B4X	1.94	*Sulfurisphaera tokodaii*
Q97U20	33	3DDJ	1.80	*Saccharolobus solfataricus*
Q97V23	40	3F8K	1.84	*Saccharolobus solfataricus P2*
Q973H2	28	3HJE	1.90	*Sulfurisphaera tokodaii str. 7*
Q973T5	28	3W9K	1.80	*Sulfurisphaera tokodaii str. 7*
Q97ZL0	34	4OIX	1.55	*Saccharolobus solfataricus P2*
Q4JA33	31	4OPC	1.40	*Sulfolobus acidocaldarius DSM 639*
Q980A5	33	4RD4	1.30	*Saccharolobus solfataricus P2*
Q97ZS3	33	4WYH	1.95	*Saccharolobus solfataricus P2*
Q96YD0	32	4ZBX	1.70	*Sulfurisphaera tokodaii str. 7*
Q4J9H4	14	5A8I	1.75	*Sulfolobus acidocaldarius*
Q06121	33	5AN7	1.10	*Saccharolobus solfataricus*
F0NG49	16	5EWT	1.80	*Sulfolobus islandicus REY15A*
C3MWA1	34	5FAD	1.87	*Sulfolobus islandicus M.14.25*
A0A0E3GTJ4	23	5N41	1.35	*Saccharolobus solfataricus*
Q980F8	34	6IQC	1.49	*Saccharolobus solfataricus P2*
Q973C7	31	7DQT	1.13	*Sulfurisphaera tokodaii str. 7*
Q97VZ7	34	7MPD	1.05	*Saccharolobus solfataricus*

Table S13. List of the 65 protein families from *Thermococcales*. For each protein family, the number of homologous sequences, the ID of the monomer used as reference from the PDB, its resolution in Å, and the source organism are provided.

Protein family ID	Number of of sequences	PDB ID	Resolution (Å)	Source organism
O57811	43	1G8A	1.40	*Pyrococcus horikoshii*
O58727	43	1IM5	1.65	*Pyrococcus horikoshii*
O50082	43	1IXK	1.90	*Pyrococcus horikoshii*
O59300	43	1J3A	1.60	*Pyrococcus horikoshii*
Q8TZR3	42	1JG1	1.20	*Pyrococcus furiosus*
Q9V1G0	43	1KK1	1.80	*Pyrococcus abyssi*
O74023	43	1MGT	1.80	*Thermococcus kodakarensis KOD1*
Q8U440	36	1RYQ	1.38	*Pyrococcus furiosus*
Q9V2Z6	42	1UA4	1.90	*Pyrococcus furiosus*
O58558	13	1V30	1.40	*Pyrococcus horikoshii*
O57934	43	1V33	1.80	*Pyrococcus horikoshii*
O59543	43	1V77	1.80	*Pyrococcus horikoshii*
O57770	44	1VAJ	1.82	*Pyrococcus horikoshii*
O57823	42	1VGJ	1.94	*Pyrococcus horikoshii*
Q8U3C7	43	1VJK	1.51	*Pyrococcus furiosus*
P29160	44	1W41	1.70	*Thermococcus celer*
O59071	43	1WL8	1.45	*Pyrococcus horikoshii*
O59248	36	1X0T	1.60	*Pyrococcus horikoshii OT3*
Q8U2V3	20	1Y81	1.70	*Pyrococcus furiosus*
Q8U306	43	1YQT	1.90	*Pyrococcus furiosus*
Q8U3E5	39	1ZD0	1.70	*Pyrococcus furiosus*
Q9V2J8	42	2BFW	1.80	*Pyrococcus abyssi*
P56709	43	2CFM	1.80	*Pyrococcus furiosus*
O59174	43	2CYJ	1.50	*Pyrococcus horikoshii OT3*
O58107	23	2CZ9	1.50	*Pyrococcus horikoshii*
P62009	43	2CZW	1.90	*Pyrococcus horikoshii*
O58836	30	2D59	1.65	*Pyrococcus horikoshii OT3*
O58764	30	2HD9	1.35	*Pyrococcus horikoshii OT3*
Q9UXT7	43	2IVN	1.65	*Pyrococcus abyssi*
Q9UZR7	43	2JJQ	1.80	*Pyrococcus abyssi*
O58461	43	2P8T	1.80	*Pyrococcus horikoshii OT3*
Q9V0G8	43	2V7F	1.15	*Pyrococcus abyssi*
P58502	17	2Z2Z	1.87	*Thermococcus kodakarensis KOD1*
O57890	36	3CG3	1.80	*Pyrococcus horikoshii*
O58465	42	3D79	1.73	*Pyrococcus horikoshii*
Q8U051	43	3E70	1.97	*Pyrococcus furiosus*
Q8U2I8	20	3ICJ	1.95	*Pyrococcus furiosus*
Q5JDJ7	43	3N98	1.87	*Thermococcus kodakarensis*

P84142	43	3TNV	1.60	*Pyrococcus horikoshii OT3*
Q8TZE5	43	3V68	1.56	*Pyrococcus furiosus DSM 3638*
Q8U092	10	4AAJ	1.75	*Pyrococcus furiosus*
P24297	41	4AR5	1.00	*Pyrococcus furiosus*
Q9V138	43	4MNO	1.35	*Pyrococcus abyssi*
B6YTD6	44	4YGS	1.70	*Thermococcus onnurineus NA1*
Q5JID5	43	5E71	1.70	*Thermococcus kodakarensis KOD1*
O57940	42	5EQT	1.94	*Pyrococcus horikoshii*
Q8TZJ1	40	5G59	1.61	*Pyrococcus furiosus*
O59021	33	5GUA	1.50	*Pyrococcus horikoshii OT3*
O59521	43	5H7K	1.60	*Pyrococcus horikoshii OT3*
Q9V2G1	43	5HJM	1.76	*Pyrococcus abyssi GE5*
O74036	28	5J4L	1.13	*Pyrococcus furiosus DSM 3638*
O57767	40	5KTN	1.34	*Pyrococcus horikoshii OT3*
Q5JD03	42	5X0J	1.43	*Thermococcus kodakarensis KOD1*
Q8TZE0	43	5X4K	1.75	*Pyrococcus furiosus DSM 3638*
Q8X270	42	5Z59	1.70	*Thermococcus kodakarensis KOD1*
Q5JIH7	43	5ZBY	1.59	*Thermococcus kodakarensis KOD1*
P58193	43	5ZCY	1.50	*Pyrococcus horikoshii OT3*
Q5JD38	42	6EMT	1.80	*Thermococcus kodakarensis*
A0A0Q2QQ54	21	6IAH	1.75	*Thermococcus thioreducens*
F8AFT0	42	6L7Q	1.16	*Pyrococcus yayanosii CH1*
O58216	43	6Q7R	1.50	*Pyrococcus horikoshii*
Q8TZI9	40	7E2B	1.85	*Pyrococcus furiosus DSM 3638*
Q9UZY0	43	7OLB	1.10	*Pyrococcus abyssi GE5*
H3ZKG9	7	7QSS	1.56	*Thermococcus litoralis*
Q5JDQ4	43	7VNX	1.80	*Thermococcus kodakarensis*

Bibliography

1. Edelsbrunner H & Harer JL (2010) *Computational Topology: An Introduction* (American Mathematical Society, Providence, RI) pp I-XII, 1-241.
2. Carlsson G (2009) Topology and Data. *B Am Math Soc* 46(2):255-308.
3. Zomorodian A & Carlsson G (2005) Computing Persistent Homology. *Discrete Comput Geom* 33:249-274.
4. Munkres JR (1984) *Elements of algebraic topology* (Addison-Wesley Publishing Company, Menlo Park, CA) p ix+454.
5. Nanda V (2021) Computational algebraic topology lecture notes.
6. Hatcher A (2002) *Algebraic topology* (Cambridge University Press, Cambridge).
7. Barannikov SA (1994) The Framed Morse complex and its invariants. *Singularities and Bifurcations,* Advances in Soviet Mathematics, (American Mathematical Society, Providence, RI), Vol 21, pp 93-115.
8. Frosini P & Landi C (1999) Size theory as a topological tool for computer vision. *Pattern Recognition And Image Analysis* 9(4):596-603.
9. Robins V (1999) Towards computing homology from finite approximations. in *14th Summer Conference on General Topology and its Applications* (Brookville, NY), pp 503-532.
10. Edelsbrunner H, Letscher D, & Zomorodian A (2002) Topological persistence and simplification. *Discrete & Computational Geometry* 28(4):511-533.
11. Bubenik P (2015) Statistical topological data analysis using persistence landscapes. *Journal of Machine Learning Research* 16(2015):77-102.
12. Bonis T, Ovsjanikov M, Oudot S, & Chazal F (2016) Persistence-Based Pooling for Shape Pose Recognition. in *Proceedings of the 6th International Workshop on Computational Topology in Image Context - Volume 9667*, ed Springer-Verlag, pp 19-29.
13. Chazal F, Guibas JL, Oudot SY, & Skraba P (2013) Persistence-Based Clustering in Riemannian Manifolds. *Journal of the ACM* 60(6):1-38.
14. Hamilton G, Dore T, & Plumberg C (2022) Applications of Persistent Homology in Nuclear Collisions. *Phys. Rev. C* 106(6).
15. Heydenreich S, Brück B, & Harnois-Déraps J (2021) Persistent homology in cosmic shear: Constraining parameters with topological data analysis. *A&A* 648.
16. Kramár M, Goullet A, Kondic L, & Mischaikow K (2013) Persistence of force networks in compressed granular media. *Physical review. E, Statistical, nonlinear, and soft matter physics* 87(4):042207.
17. Kramár M*, et al.* (2016) Analysis of Kolmogorov flow and Rayleigh–Bénard convection using persistent homology. *Physica D: Nonlinear Phenomena* 334:82-98.
18. Duman AN & Pirim H (2018) Gene Coexpression Network Comparison via Persistent Homology. *Int J Genomics* 2018:7329576.
19. Meng Z, Anand DV, Lu Y, Wu J, & Xia K (2020) Weighted persistent homology for biomolecular data analysis. *Scientific reports* 10(1):2079.
20. Iqbal Z, Gandhi SD, Kersten JR, & Pagel PS (2007) An unusual right atrial structure in a patient with a new diastolic murmur. *J Cardiothorac Vasc Anesth* 21(1):152-154.
21. Lawson P, Sholl AB, Brown JQ, Fasy BT, & Wenk C (2019) Persistent Homology for the Quantitative Evaluation of Architectural Features in Prostate Cancer Histology. *Scientific reports* 9(1):1139.
22. Nicolau M, Levine AJ, & Carlsson G (2011) Topology based data analysis identifies a subgroup of breast cancers with a unique mutational profile and excellent survival. *Proceedings of the National Academy of Sciences of the United States of America* 108(17):7265-7270.
23. Vandaele R, Mukherjee P, Selby HM, Shah RP, & Gevaert O (2023) Topological data analysis of thoracic radiographic images shows improved radiomics-based lung tumor histology prediction. *Patterns (N Y)* 4(1):100657.
24. Li M*, et al.* (2018) Topological Data Analysis as a Morphometric Method: Using Persistent Homology to Demarcate a Leaf Morphospace. *Front Plant Sci* 9:553.
25. Li M*, et al.* (2018) The Persistent Homology Mathematical Framework Provides Enhanced Genotype-to-Phenotype Associations for Plant Morphology. *Plant Physiol* 177(4):1382-1395.
26. Xia K & Wei GW (2014) Persistent homology analysis of protein structure, flexibility, and folding. *Int J Numer Method Biomed Eng* 30(8):814-844.

27. Kovacev-Nikolic V, Bubenik P, Nikolic D, & Heo G (2016) Using persistent homology and dynamical distances to analyze protein binding. *Stat Appl Genet Mol Biol* 15(1):19-38.
28. Ichinomiya T, Obayashi I, & Hiraoka Y (2020) Protein-Folding Analysis Using Features Obtained by Persistent Homology. *Biophys J* 118(12):2926-2937.
29. Gameiro M*, et al.* (2015) A topological measurement of protein compressibility. *Japan Journal of Industrial and Applied Mathematics* 32:1-17.
30. Benjamin K*, et al.* (2023) Homology of homologous knotted proteins. *Journal of the Royal Society, Interface / the Royal Society* 20(201):20220727.
31. Otter N, Porter MA, Tillmann U, Grindrod P, & Harrington HA (2017) A roadmap for the computation of persistent homology. *EPJ Data Science* 6.
32. Bauer U (2016) Ripser).
33. Morozov D (2023) Dionysus).
34. Maria C, Boissonnat JD, Glisse M, & Yvinec M (2014) The Gudhi Library: Simplicial Complexes and Persistent Homology. *ICMS 2014*, eds Hong H & Yap C (Springer Berlin Heidelberg, Berlin, Heidelberg), pp 167-174.
35. Cohen-Steiner D, Edelsbrunner H, & Harer J (2007) Stability of Persistence Diagrams. *Discrete & Computational Geometry* 37(1):103-120.
